# Estimates of the global, regional, and national morbidity, mortality, and aetiologies of diarrhoea in 195 countries: a systematic analysis for the Global Burden of Disease Study 2016

**DOI:** 10.1016/S1473-3099(18)30362-1

**Published:** 2018-11

**Authors:** GBD 2016 Diarrhoeal Disease Collaborators, Christopher Troeger, Brigette F Blacker, Ibrahim A Khalil, Puja C Rao, Shujin Cao, Stephanie RM Zimsen, Samuel B Albertson, Jeffery D Stanaway, Aniruddha Deshpande, Zegeye Abebe, Nelson Alvis-Guzman, Azmeraw T Amare, Solomon W Asgedom, Zelalem Alamrew Anteneh, Carl Abelardo T Antonio, Olatunde Aremu, Ephrem Tsegay Asfaw, Tesfay Mehari Atey, Suleman Atique, Euripide Frinel G Arthur Avokpaho, Ashish Awasthi, Henok Tadesse Ayele, Aleksandra Barac, Mauricio L Barreto, Quique Bassat, Saba Abraham Belay, Isabela M Bensenor, Zulfiqar A Bhutta, Ali Bijani, Hailemichael Bizuneh, Carlos A Castañeda-Orjuela, Abel Fekadu Dadi, Lalit Dandona, Rakhi Dandona, Huyen Phuc Do, Manisha Dubey, Eleonora Dubljanin, Dumessa Edessa, Aman Yesuf Endries, Babak Eshrati, Tamer Farag, Garumma Tolu Feyissa, Kyle J Foreman, Mohammad H Forouzanfar, Nancy Fullman, Peter W Gething, Melkamu Dedefo Gishu, William W Godwin, Harish Chander Gugnani, Rahul Gupta, Gessessew Bugssa Hailu, Hamid Yimam Hassen, Desalegn Tsegaw Hibstu, Olayinka S Ilesanmi, Jost B Jonas, Amaha Kahsay, Gagandeep Kang, Amir Kasaeian, Yousef Saleh Khader, Ibrahim A Khalil, Ejaz Ahmad Khan, Muhammad Ali Khan, Young-Ho Khang, Niranjan Kissoon, Sonali Kochhar, Karen L Kotloff, Ai Koyanagi, G Anil Kumar, Hassan Magdy Abd El Razek, Reza Malekzadeh, Deborah Carvalho Malta, Suresh Mehata, Walter Mendoza, Desalegn Tadese Mengistu, Bereket Gebremichael Menota, Haftay Berhane Mezgebe, Fitsum Weldegebreal Mlashu, Srinivas Murthy, Gurudatta A Naik, Cuong Tat Nguyen, Trang Huyen Nguyen, Dina Nur Anggraini Ningrum, Felix Akpojene Ogbo, Andrew Toyin Olagunju, Deepak Paudel, James A Platts-Mills, Mostafa Qorbani, Anwar Rafay, Rajesh Kumar Rai, Saleem M Rana, Chhabi Lal Ranabhat, Davide Rasella, Sarah E Ray, Cesar Reis, Andre MN Renzaho, Mohammad Sadegh Rezai, George Mugambage Ruhago, Saeid Safiri, Joshua A Salomon, Juan Ramon Sanabria, Benn Sartorius, Monika Sawhney, Sadaf G Sepanlou, Mika Shigematsu, Mekonnen Sisay, Ranjani Somayaji, Chandrashekhar T Sreeramareddy, Bryan L Sykes, Getachew Redae Taffere, Roman Topor-Madry, Bach Xuan Tran, Kald Beshir Tuem, Kingsley Nnanna Ukwaja, Stein Emil Vollset, Judd L Walson, Marcia R Weaver, Kidu Gidey Weldegwergs, Andrea Werdecker, Abdulhalik Workicho, Muluken Yenesew, Biruck Desalegn Yirsaw, Naohiro Yonemoto, Maysaa El Sayed Zaki, Theo Vos, Stephen S Lim, Mohsen Naghavi, Christopher JL Murray, Ali H Mokdad, Simon I Hay, Robert C Reiner

## Abstract

**Background:**

The Global Burden of Diseases, Injuries, and Risk Factors Study (GBD) 2016 provides an up-to-date analysis of the burden of diarrhoea in 195 countries. This study assesses cases, deaths, and aetiologies in 1990–2016 and assesses how the burden of diarrhoea has changed in people of all ages.

**Methods:**

We modelled diarrhoea mortality with a Bayesian hierarchical modelling platform that evaluates a wide range of covariates and model types on the basis of vital registration and verbal autopsy data. We modelled diarrhoea incidence with a compartmental meta-regression tool that enforces an association between incidence and prevalence, and relies on scientific literature, population representative surveys, and health-care data. Diarrhoea deaths and episodes were attributed to 13 pathogens by use of a counterfactual population attributable fraction approach. Diarrhoea risk factors are also based on counterfactual estimates of risk exposure and the association between the risk and diarrhoea. Each modelled estimate accounted for uncertainty.

**Findings:**

In 2016, diarrhoea was the eighth leading cause of death among all ages (1 655 944 deaths, 95% uncertainty interval [UI] 1 244 073–2 366 552) and the fifth leading cause of death among children younger than 5 years (446 000 deaths, 390 894–504 613). Rotavirus was the leading aetiology for diarrhoea mortality among children younger than 5 years (128 515 deaths, 105 138–155 133) and among all ages (228 047 deaths, 183 526–292 737). Childhood wasting (low weight-for-height score), unsafe water, and unsafe sanitation were the leading risk factors for diarrhoea, responsible for 80·4% (95% UI 68·2–85·0), 72·1% (34·0–91·4), and 56·4% (49·3–62·7) of diarrhoea deaths in children younger than 5 years, respectively. Prevention of wasting in 1762 children (95% UI 1521–2170) could avert one death from diarrhoea.

**Interpretation:**

Substantial progress has been made globally in reducing the burden of diarrhoeal diseases, driven by decreases in several primary risk factors. However, this reduction has not been equal across locations, and burden among adults older than 70 years requires attention.

**Funding:**

Bill & Melinda Gates Foundation.

## Introduction

Diarrhoea is a global problem. In 2016, diarrhoea was the eighth leading cause of mortality, responsible for more than 1·6 million deaths.^1^ More than a quarter (26·93%) of diarrhoeal deaths occurred among children younger than 5 years, and about 90% (89·37%) of diarrhoeal deaths occurred in south Asia and sub-Saharan Africa.^1^ Evidence shows that diarrhoeal diseases disproportionately affect locations with poor access to health care, safe water, and sanitation, and low-income or marginalised populations.^2^ These observations illustrate that although challenges exist, diarrhoea mortality is largely avoidable and renewed efforts to reduce disease burden are urgently needed.

Diarrhoea is present globally among all regions and populations. However, an inequitable proportion of diarrhoea morbidity and mortality occurs in low-income countries, which have fewer resources and less robust infrastructure to manage burden than high-income countries.^3^ In recognising the magnitude of this burden, the global health community has made prevention and treatment of diarrhoeal diseases a priority. For example, in 2013, WHO and UNICEF coordinated the Diarrhoea and Pneumonia Interventions Study Group that developed the Global Action Plan for the Prevention and Control of Pneumonia and Diarrhoea and an accompanying *Lancet* Series.^4^, ^5^ This plan established goals to reduce severe incidence and deaths due to diarrhoea in children by 2025. Through promoting various effective intervention and treatment strategies, the Global Action Plan for the Prevention and Cox Pneumonia and Diarrhoea targets mortality reductions to one in 1000 and reductions in the incidence of severe diarrhoea to 75% of the country-specific levels in 2010.

**Research in context**

**Evidence before this study**

Despite substantial improvements in global health, diarrhoeal diseases have had a large health impact over the past few decades. Since 1990, diarrhoea has been ranked among the top ten causes of death and disability-adjusted life-years (DALYs) among all ages, and one of the top five causes of death and DALYs for children younger than 5 years. Multiple groups including the Global Burden of Disease, Injuries, and Risk Factors Study (GBD) have measured the burden of diarrhoeal diseases, one of the leading causes of morbidity and mortality globally. Within the past year, numerous publications have described national, regional, and global patterns of disease. The GBD 2015 study found that diarrhoeal diseases were the ninth leading cause of mortality worldwide in that year, causing about 1·31 million deaths (95% uncertainty interval [UI] 1·23–1·39) among all ages, and disproportionately affecting children younger than 5 years (fourth leading cause; 499 000 deaths, 95% UI 447 000–558 000). Furthermore, in 2015, an estimated 2·39 billion episodes (95% UI 2·30–2·50) of diarrhoeal diseases occurred and an estimated 71 590 000 episodes (66 443 000–77 206 000) were DALYS attributable to diarrhoea.

**Added value of this study**

This analysis incorporates 290 310 new mortality and 5695 new morbidity datapoints from the previous GBD cycle, and has increased the granularity of estimates by including 183 new subnational locations. This study improves on previous GBD studies by focusing on changes in diarrhoea burden from 2015 to 2016, exploring the association between case-fatality ratio and Socio-demographic Index, providing insight into burden among the youngest and oldest age groups, and focusing on quantifiable evidence for the most efficient and effective interventions to help guide strategies to target risk factors unique to each location.

**Implications of all the available evidence**

The epidemiology of diarrhoeal disease is changing. Declines in mortality, particularly among children younger than 5 years, are potentially offset by ageing populations and a growing burden in people older than 70 years. Expansion of access to the rotavirus vaccine, improvement of child growth and wellbeing, and provision of universal access to safe water and sanitation are necessary to reduce further the preventable disease burden due to diarrhoea.

The Global Burden of Disease, Injuries, and Risk Factors Study (GBD) is a systematic, comprehensive, and annual effort to quantify the impact of more than 200 diseases and 80 risk factors.^1^, ^6^, ^7^ We present an update to previously published estimates of diarrhoea morbidity, mortality, and risk factors^2^, ^8^ based on the results in GBD 2016. We focus on changes to the methodology that have improved the precision and accuracy of our estimates, and on instances in which our results have diverged from previous GBD iterations. Additionally, we discuss interventions and treatments that could help to guide targeted efforts to reduce diarrhoea burden.

## Methods

### Overview

The GBD study estimates prevalence, incidence, and mortality of diarrhoeal disease by country, age, sex, and year. Uncertainty in diarrhoea estimates are maintained through the modelling process by use of draws from a posterior distribution and is presented as 2·5th and 97·5th percentiles of the distribution. Detailed methods of the GBD study and diarrhoea estimation have been previously published.^1^, ^2^, ^6^, ^7^ Updated flow charts, input data for the models, and analytical code are made publicly available in compliance with the Guidelines for Accurate and Transparent Health Estimates Reporting.^9^ Detailed methodology pertaining specifically to diarrhoea estimation is provided in the appendix.

We modelled diarrhoea mortality in the Cause of Death Ensemble model (CODEm) platform, which is a Bayesian, hierarchical, space–time, ensemble model tool (appendix p 4).^1^, ^10^ CODEm produces a wide variety of submodels designed to include a diverse set of covariates (eg, safe water and sanitation and childhood under-nutrition) and model types (eg, spatiotemporal Gaussian process regression and mixed-effects models) to create a predictive model for causes of death. A subset (15%) of available data are withheld and each submodel is weighted on the basis of out-of-sample predictive validity, contributing proportionally to a final set of 1000 draws. A draw is a single realisation from an uncertainty distribution. These predictive regression models produce estimates of cause-specific mortality based on vital registration, verbal autopsy, and surveillance system data. The GBD 2016 cycle expanded its data sources by adding 169 country-years of vital registration and 24 new verbal autopsy studies, including Sample Registration System data for 2004–13 shared by the Government of India for each state stratified by urban or rural residence and for new data from the WHO Mortality Database released since GBD 2015.^1^ We added several new covariates for the selection algorithm for GBD 2016 including prevalence of childhood wasting and underweight, vitamin A deficiency, zinc deficiency, health-care access and quality, and safe handwashing. A complete list of covariates used in the models can be found in the appendix (p 6). A core component of cause of death estimation is that the sum of cause-specific mortality estimates should be equal to the all-cause mortality estimate from the GBD study. This calculation occurs in a process called CoDCorrect in which the modelled values for diarrhoea are scaled on the basis of the uncertainty of those values.^1^

We modelled diarrhoea incidence in DisMod-MR, version 2.1 (DisMod). DisMod is a Bayesian, hierarchical meta-regression tool (appendix p 8) that contains a compartmental model in which incidence, prevalence, and mortality are related in a series of ordinary differential equations.^6^ Input data for these models come from scientific literature, population representative surveys, and records of hospital and health-care facility use. We expanded the database for diarrhoea modelling in the 2016 cycle to include 139 new sources and 5696 new datapoints. As diarrhoea is seasonal in many locations, we introduced a method to adjust for data sources that were less than a year in duration by fitting a sine-cosine model with a period of 6 months for each GBD region and adjusting the diarrhoea prevalence on the basis of the predicted deviation from the mean (appendix p 9). This model includes data from a variety of case definitions and DisMod internally estimates an adjustment factor for a non-reference definition to the reference definition. The reference definition of diarrhoea is three or more abnormally loose stools in a 24-h period. We took the coefficients of this adjustment for inpatient admission to hospital to estimate the number of admissions to hospital for diarrhoea at the global level.

The attribution of 13 diarrhoeal aetiologies, identified as those significantly associated with moderate-to-severe diarrhoea in the Global Enteric Multicenter Study, was estimated separately from mortality and morbidity. The majority of diarrhoeal aetiologies were attributed with a counterfactual approach called a population attributable fraction (PAF).^2^ Our approach accounted for pathogen co-detection and detection in healthy individuals, and does not necessitate a one pathogen to one episode association. PAF is defined as the product of the modelled proportion of pathogen detection in diarrhoea samples based on a molecular diagnostic case definition and the odds ratio (OR) of diarrhoea given the detection of a pathogen: ^11^

$$PAF=Proportion\times \left(1-\frac{1}{OR}\right)$$

ORs are based on molecular diagnostic results from the Global Enteric Multicenter Study.^12^, ^13^ By contrast with previous rounds of GBD that followed the Global Enteric Multicenter Study age groups, for GBD 2016, we defined ORs for children younger than 1 year and all age groups older than 1 year. This approach makes these ORs consistent with the GBD age groups and adds power to the ORs of 1–5 years that are applied to all age groups older than 5 years. The proportion estimates are from DisMod models and their input data from scientific literature and modelled for each age, sex, year, and location. The input data for these models, including meta-data about the sources, age groups, and types of diagnostics, are provided in the appendix (pp 3–37). The number of episodes and deaths attributable to each aetiology is the product of the total number of diarrhoea episodes and deaths, and the PAF for that aetiology.

### Risk factor attribution and decomposition

Risk factors for diarrhoeal diseases were modelled independently with a comparative risk assessment framework. Detailed descriptions have been published elsewhere.^7^ Like the diarrhoeal aetiologies, risk factors are modelled assuming a counterfactual population. The exposure level in a population for a given risk factor was modelled with DisMod-MR and spatiotemporal Gaussian process regression, depending on the risk factor. Relative risks for diarrhoea by risk factor and at each exposure level were assessed, usually from published meta-analyses.

To assess the efficiency of targeted interventions for each risk factor, we took advantage of the counterfactual definition of risk factor burden, such that the diarrhoea mortality rate due to each risk factor is equivalent to the reduction expected given complete absence of the risk factor. The number needed to treat is an epidemiological concept for which the rate of disease in two populations is compared and is defined as:^14^

$$\frac{1}{\mathrm{Attributable risk reduction}}$$

Attributable risk reduction is defined as the difference in the rates between two populations. Because the counterfactual rate of disease is the difference between diarrhoea mortality rate and mortality rate due to the risk factor, the number needed to treat is the inverse of diarrhoea mortality rate due to that risk factor.

To determine the contribution of the leading ten risk factors for diarrhoea on the overall change in diarrhoea mortality rate among children younger than 5 years between 2000 and 2016, we used a combinatorial process to determine the relative contribution of each risk factor to the change in diarrhoea disability-adjusted life-years (DALYs).^2^, ^8^, ^15^ Decomposition for each risk factor was done independently and assessed the change in diarrhoea mortality due to the risk factor, population growth, and population ageing; the remaining change was considered part of the unexplained diarrhoea cause rate. These analyses are not done at the draw level so uncertainty is not propagated through risk factor decomposition.

### Role of the funding source

The funders of the study had no role in study design, data collection, data analysis, data interpretation, or writing of the report. The corresponding author had full access to all data in the study and had final responsibility for the decision to submit for publication.

## Results

**Figure 1:**
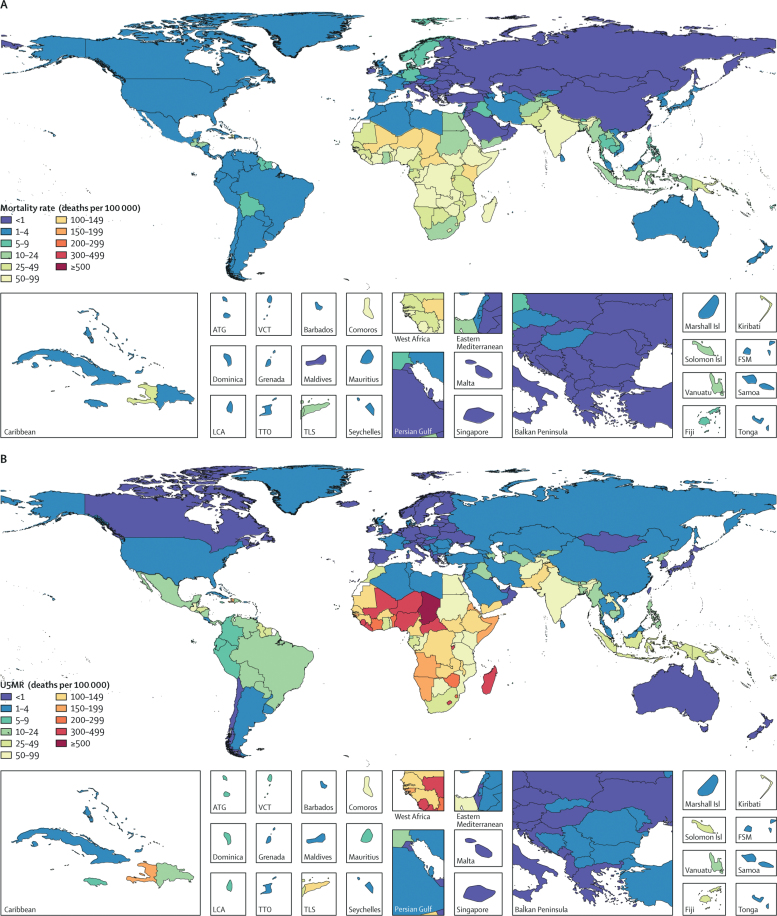

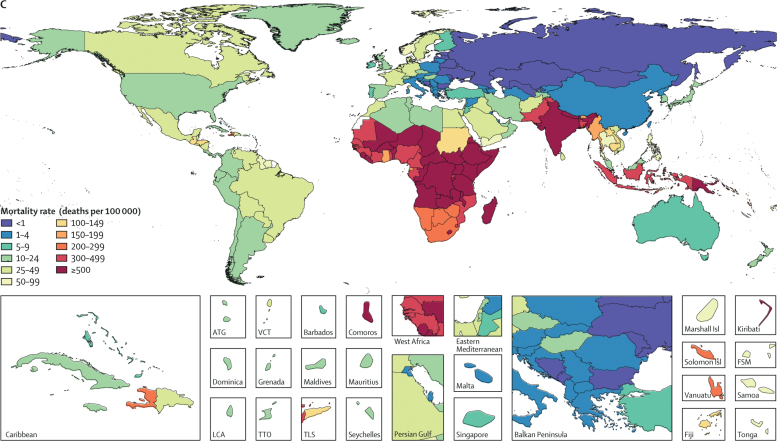
Diarrhoea mortality rate in 2016 Diarrhoea mortality rate in individuals of all ages (A), children younger than 5 years (B), and adults older than 70 years (C). U5MR=under-5 mortality rate. ATG=Antigua and Barbuda. VCT=Saint Vincent and the Grenadines. LCA=Saint Lucia. TTO=Trinidad and Tobago. Isl=Islands. FSM=Federated States of Micronesia. TLS=Timor-Leste.

We estimated that in 2016, diarrhoea was the eighth leading cause of death among all ages (1 655 944 deaths, 95% UI 1 244 073–2 366 552; table 1) and the fifth leading cause of death among children younger than 5 years (446 000 deaths, 390 894–504 613; table 1). Overall, the diarrhoea mortality was 22·4 deaths (16·8–32·0) per 100 000 in 2016 with higher rates among children younger than 5 years (70·6 deaths [61·9–79·8] per 100 000) and among adults older than 70 years (171·7 deaths [114·1–263·5] per 100 000; table 1, figure 1). The highest rate of diarrhoea mortality among children younger than 5 years occurred in Chad (499 deaths [345–686] per 100 000), the Central African Republic (384·2 deaths [237–596] per 100 000), and Niger (376 deaths [234–559] per 100 000; figure 1). Diarrhoea was responsible for 8·92% (95% UI 7·95–9·94) of all deaths in children younger than 5 years in 2016, with a higher share of deaths in girls younger than 5 years (9·02%, 7·76–10·47) than in boys of the same age (8·84%, 7·58–10·22). Among children younger than 5 years, we estimated 1 105 406 865 episodes (95% UI 961 595 610–1 274 767 300) of diarrhoea in 2016 and 1·75 episodes (1·52–2·02) per child younger than 5 years (table 1). Diarrhoea was the third leading cause of DALYs in 2016, responsible for 74·4 million DALYs (95% UI 63·4–93·4), and 40·1 million (63%) of those occurred among children younger than 5 years (35·5–45·1 million).

**Table 1:** Episodes and deaths among all ages, children younger than 5 years, and adults older than 70 years, in 2016, by Global Burden of Disease regions and super-regions

		**All ages**				**Younger than 5 years**				**Older than 70 years**			
		Deaths (95% UI)	Deaths per 100 000 (95% UI)	Episodes (95% UI)	Episodes per person-year (95% UI)	Deaths (95% UI)	Deaths per 100 000 (95% UI)	Episodes (95% UI)	Episodes per person-year (95% UI)	Deaths (95% UI)	Deaths per 100 000 (95% UI)	Episodes (95% UI)	Episodes per person-year (95% UI)
Global		1 655 944 (1 244 073–2 366 552)	22·4 (16·8–32·0)	4 480 400 603 (4 246 997 396–4 737 769 159)	0·61 (0·57–0·64)	446 000 (390 894–504 613)	70·6 (61·9–79·8)	1 105 406 865 (961 595 610–1 274 767 300)	1·75 (1·52–2·02)	694 010 (461 118–1 065 409)	171·7 (114·1–263·5)	364 929 495 (331 940 378–403 163 623)	0·90 (0·82–1·00)
High income		31 267 (29 970–32 742)	2·9 (2·8–3·1)	141 626 741 (133 506 539–150 142 971)	0·13 (0·13–0·14)	761 (693–839)	1·3 (1·2–1·5)	33 388 923 (27 829 613–40 606 214)	0·58 (0·48–0·70)	26 909 (25 688–28 313)	20·1 (19·2–21·2)	22 601 582 (21 031 917–24 284 703)	0·17 (0·16–0·18)
	High-income North America	10 919 (10 506–11 345)	3·0 (2·9–3·2)	48 921 976 (46 533 601–51 532 621)	0·14 (0·13–0·14)	394 (352–440)	1·8 (1·6–2·0)	9 056 709 (7 763 965–10 672 399)	0·42 (0·36–0·49)	8 740 (8359–9111)	24·5 (23·4–25·5)	4 023 411 (3 746 710–4 317 044)	0·11 (0·10–0·12)
	Australasia	322 (291–355)	1·1 (1·0–1·2)	2 166 116 (2 031 289–2 311 178)	0·08 (0·07–0·08)	12 (9–15)	0·7 (0·5–0·9)	234 468 (189 996–290 629)	0·13 (0·11–0·16)	276 (245–308)	9·7 (8·6–10·8)	231 951 (211 932–252 135)	0·08 (0·07–0·09)
	High-income Asia Pacific	4126 (3567–5137)	2·3 (2·0–2·8)	9 858 030 (9 308 375–10 455 512)	0·05 (0·05–0·06)	60 (51–68)	0·8 (0·7–0·9)	1 467 326 (1 207 565–1 789 585)	0·20 (0·16–0·24)	3 625 (3145–4472)	12·2 (10·6–15·0)	2 000 718 (1 847 476–2 173 129)	0·07 (0·06–0·07)
	Western Europe	14 686 (13 841–15 540)	3·4 (3·2–3·6)	65 408 598 (61 299 348–69 872 055)	0·15 (0·14–0·16)	152 (131–176)	0·7 (0·6–0·8)	16 600 090 (13 553 833–20 598 003)	0·76 (0·62–0·94)	13 405 (12 615–14 258)	22·3 (21·0–23·7)	15 539 967 (14 442 061–16 731 794)	0·26 (0·24–0·28)
	Southern Latin America	1214 (1105–1329)	1·9 (1·7–2·0)	15 272 023 (13 990 392–16 698 862)	0·23 (0·21–0·26)	144 (113–184)	2·9 (2·2–3·7)	6 030 331 (4 929 130–7 365 567)	1·20 (0·98–1·47)	862 (770–965)	17·1 (15·3–19·1)	805 535 (744 222–878 029)	0·16 (0·15–0·17)
Central Europe, eastern Europe, and central Asia		3372 (2808–4 47)	0·8 (0·7–1·0)	163 543 108 (154 189 373–174 379 708)	0·39 (0·37–0·42)	1 943 (1 411–2 716)	6·9 (5·0–9·6)	50 878 849 (43 957 060–58 895 977)	1·80 (1·56–2·09)	777 (700–866)	2·1 (1·9–2·3)	15 964 724 (14 405 434–17 721 628)	0·43 (0·39–0·48)
	Eastern Europe	550 (456–655)	0·3 (0·2–0·3)	79 911 704 (75 379 533–85 158 226)	0·38 (0·36–0·40)	180 (129–246)	1·4 (1·0–1·9)	23 661 809 (20 848 512–27 184 316)	1·83 (1·61–2·10)	166 (134–207)	0·8 (0·6–1·0)	7 883 083 (6 983 415–8 844 997)	0·38 (0·34–0·43)
	Central Europe	785 (713–857)	0·7 (0·6–0·7)	47 622 487 (44 633 208–50 845 630)	0·41 (0·39–0·44)	73 (60–88)	1·3 (1·1–1·6)	14 195 427 (11 976 777–17 026 634)	2·54 (2·14–3·05)	548 (494–608)	4·2 (3·8–4·7)	7 011 015 (6 423 089–7 705 460)	0·54 (0·50–0·60)
	Central Asia	2 037 (1 496–2 806)	2·3 (1·7–3·2)	36 008 917 (33 378 397–39 089 551)	0·41 (0·38–0·44)	1 690 (1 171–2 454)	17·5 (12·1–25·4)	13 021 613 (11 021 848–15 425 971)	1·35 (1·14–1·60)	64 (44–87)	1·9 (1·3–2·6)	1 070 626 (954 834–1 193 367)	0·32 (0·29–0·36)
Latin America and Caribbean		24 026 (21 716–27 907)	4·2 (3·8–4·8)	455 080 817 (430 795 166–481 238 663)	0·79 (0·75–0·83)	8 828 (7 589–10 522)	17·8 (15·3–21·2)	140 034 494 (123 896 675–157 523 720)	2·82 (2·50–3·18)	8 830 (7885–10 715)	32·1 (28·6–38·9)	44 369 109 (41 355 008–47 985 257)	1·61 (1·50–1·74)
	Central Latin America	10 603 (9827–11 651)	4·2 (3·9–4·6)	145 541 713 (136 220 654–155 309 571)	0·57 (0·54–0·61)	3 552 (3 154–4 093)	15·6 (13·8–17·9)	42 745 877 (37 076 669–49 851 781)	1·87 (1·62–2·18)	3904 (3553–4426)	35·6 (32·4–40·4)	11 216 119 (10 366 913–12 206 833)	1·02 (0·95–1·11)
	Andean Latin America	1 898 (1 387–2 873)	3·2 (2·3–4·8)	51 849 096 (48 728 296–55 059 137)	0·87 (0·81–0·92)	733 (554–950)	11·0 (8·3–14·3)	15 437 492 (13 322 654–17 616 648)	2·32 (2·00–2·64)	662 (395–1 168)	24·7 (14·8–43·7)	4 722 673 (4 321 225–5 183 637)	1·76 (1·61–1·94)
	Caribbean	5135 (3568–7476)	11·2 (7·8–16·3)	34 976 740 (33 008 600–37 090 931)	0·76 (0·72–0·81)	2 773 (1 724–4 440)	69·5 (43·2–111·3)	9 159 741 (7 915 572–10 575 956)	2·30 (1·98–2·65)	1180 (717–2001)	41·1 (25·0–69·8)	4 150 525 (3 810 572–4 563 391)	1·45 (1·33–1·59)
	Tropical Latin America	6196 (5911–6544)	2·9 (2·7–3·0)	222 713 268 (211 374 722–234 913 999)	1·03 (0·98–1·09)	1 686 (1 485–1 927)	10·5 (9·2–12·0)	72 691 384 (65 257 021–80 123 632)	4·51 (4·05–4·97)	3 026 (2870–3189)	27·4 (26·0–28·9)	24 279 793 (22 631 808–26 094 231)	2·20 (2·05–2·37)
Southeast Asia, East Asia, and Oceania		82 391 (52 849–114 890)	4·0 (2·5–5·5)	777 367 105 (732 380 142–826 214 236)	0·37 (0·35–0·40)	15 443 (13 267–18 208)	12·5 (10·8–14·8)	128 879 193 (108 564 727–154 165 655)	1·05 (0·88–1·25)	38 425 (19 341–56 971)	34·1 (17·2–50·6)	57 141 106 (51 364 528–63 755 385)	0·51 (0·46–0·57)
	East Asia	6443 (4668–10 215)	0·5 (0·3–0·7)	292 851 984 (273 845 010–313 093 801)	0·21 (0·19–0·22)	1 988 (1 573–2 596)	3·1 (2·4–4·0)	35 276 466 (29 295 004–43 259 235)	0·55 (0·45–0·67)	2 351 (1415–4440)	2·7 (1·6–5·1)	24 636 295 (21 886 216–27 807 635)	0·28 (0·25–0·32)
	Southeast Asia	73 484 (46 195–101 156)	11·2 (7·1–15·4)	471 272 773 (444 252 320–499 807 373)	0·72 (0·68–0·76)	13 027 (10 999–15 526)	22·8 (19·3–27·2)	91 013 337 (76 831 318–108 315 297)	1·60 (1·35–1·90)	35 063 (17 323–53 566)	140·7 (69·5–214·9)	31 904 754 (28 864 524–35 411 367)	1·28 (1·16–1·42)
	Oceania	2312 (1378–3591)	20·6 (12·3–32·0)	13 242 348 (12 409 312–14 163 414)	1·18 (1·11–1·26)	414 (224–725)	29·3 (15·9–51·3)	2 589 390 (2 167 409–3 113 582)	1·83 (1·53–2·20)	914 (492–1 605)	366·8 (197·5–643·7)	600 057 (536 232–674 469)	2·41 (2·15–2·71)
North Africa and Middle East		34 998 (26 768–44 682)	6·1 (4·7–7·8)	426 334 311 (396 205 357–457 691 871)	0·74 (0·69–0·80)	26 373 (19 539–34 818)	41·7 (30·9–55·1)	152 831 944 (131 124 308–176 495 144)	2·42 (2·08–2·79)	4751 (2708–9256)	26·9 (15·4–52·5)	15 794 118 (14 302 510–17 518 841)	0·90 (0·81–0·99)
South Asia		873 865 (605 184–1 356 359)	51·4 (35·6–79·8)	1 487 506 728 (1 408 026 882–1 568 110 857)	0·88 (0·83–0·92)	101 927 (85 817–122 100)	66·4 (55·9–79·5)	228 158 069 (198 916 167–263 837 054)	1·49 (1·29–1·72)	508 455 (337 374–798 290)	877·4 (582·2–1 377·5)	173 702 623 (156 344 871–193 583 814)	3·00 (2·70–3·34)
Sub-Saharan Africa		606 024 (469 707–798 314)	61·8 (47·9–81·5)	1 028 941 793 (966 501 198–1 098 089 853)	1·05 (0·99–1·12)	290 724 (243 545–342 557)	185·7 (155·5–218·8)	371 235 393 (322 159 662–428 790 776)	2·37 (2·06–2·74)	105 863 (63 487–166 068)	589·3 (353·4–924·4)	35 356 232 (31 983 296–39 006 426)	1·97 (1·78–2·17)
	Southern sub-Saharan Africa	24 952 (18 130–33 765)	32·4 (23·6–43·9)	80 177 323 (76 939 912–83 739 087)	1·04 (1·00–1·09)	10 281 (8 270–12 696)	119·4 (96·1–147·5)	17 539 773 (15 579 677–19 964 990)	2·04 (1·81–2·32)	5 636 (3 076–9 354)	230·3 (125·7–382·2)	4 578 637 (4 292 157–4 904 911)	1·87 (1·75–2·00)
	Western sub-Saharan Africa	270 082 (213 648–339 535)	67·8 (53·6–85·3)	380 566 260 (356 058 649–407 706 303)	0·96 (0·89–1·02)	177 262 (139 712–220 546)	274·3 (216·2–341·3)	157 133 499 (138 027 236–180 716 041)	2·43 (2·14–2·80)	28 641 (16 799–48 488)	454·0 (266·3–768·6)	11 293 766 (10 062 654–12 571 363)	1·79 (1·60–1·99)
	Eastern sub-Saharan Africa	242 217 (174 668–330 016)	62·6 (45·1–85·3)	425 972 738 (399 183 913–454 180 616)	1·10 (1·03–1·17)	72 836 (60 186–85 260)	116·4 (96·2–136·3)	137 266 734 (118 573 209–159 017 510)	2·19 (1·90–2·54)	58 013 (35 154–91 283)	807·3 (489·2–1 270·3)	15 217 020 (13 685 084–16 834 027)	2·12 (1·90–2·34)
	Central sub-Saharan Africa	68 599 (50 148–93 556)	58·2 (42·6–79·4)	142 225 473 (131 307 539–154 054 352)	1·21 (1·11–1·31)	30 306 (20 536–43 767)	145·7 (98·8–210·5)	59 295 386 (50 047 396–69 410 193)	2·85 (2·41–3·34)	13 513 (8 388–22 326)	667·7 (414·5–1 103·2)	4 266 809 (3 811 921–4 756 955)	2·11 (1·88–2·35)

UI=uncertainty interval.

Diarrhoea was the eighth leading cause of mortality among adults aged 70 years and older (171·7 deaths [95% UI 114·1–263·5] per 100 000), responsible for 694 010 deaths (461 118–1 065 409) in this age group in 2016. Diarrhoea mortality among adults older than 70 years was highest in Kenya (1877 deaths [1184–3029] per 100 000), Central African Republic (1282 deaths [680–2112] per 100 000), and India (1013 deaths [667–1578] per 100 000; figure 1). Similar to diarrhoea among children younger than 5 years, mortality among adults older than 70 years was greatest in the lowest Socio-demographic Index (SDI) quantile (773·9 deaths [490·3–1241·8] per 100 000) and lowest in the high-middle quintile of SDI (8·6 deaths [6·4–11·1] per 100 000). Although the mortality rate in adults older than 70 years was nearly three times greater than the rate in children younger than 5 years, diarrhoea incidence in adults older than 70 years was about half that of the incidence in children younger than 5 years (0·90 episodes [95% UI 0·82–1·00] per person-year).

The number of diarrhoea deaths among children younger than 5 years has decreased by 56·5% (95% UI 49·5–62·6; from 1 204 538 to 445 600) since 2000, and diarrhoea mortality in this age group has decreased by 59·3% (52·7–65·0; from 173·3 per 100 000 to 70·6 per 100 000). Diarrhoea incidence among children younger than 5 years decreased by 12·7% (10·6–14·8) between 2000 and 2016 (from 2·0 per child-year to 1·75 per child-year). Although diarrhoea mortality rate among adults older than 70 years has decreased by 31·8% (32·4–43·4) since 2000 (from 251·7 per 100 000 to 171·7 per 100 000), the number of deaths did not significantly change (2·7% increase, −14·8 to 33·3; from 675 843 to 694 010) during that time, suggesting that population ageing has increased diarrhoea burden in this age group. The greatest increase in diarrhoea mortality among adults older than 70 years occurred in high-income locations (from 7534 deaths to 25 340 deaths) including the USA, where mortality increased by 178·0% (171·6–202·1; from 8·1 per 100 000 to 23·2 per 100 000) between 2000 and 2016, and the number of deaths in this age group increased by 264·8% (245·2–283·9; from 2027 to 7396).

**Figure 2:**
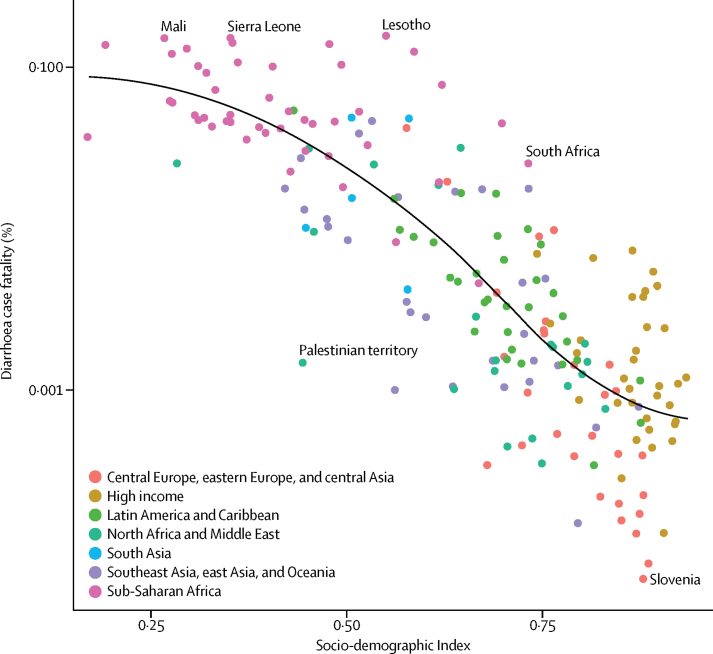
Assocation between the Socio-demographic Index and diarrhoea case fatality in children younger than 5 years in 2016 Each point represents a country.

The case-fatality ratio (CFR) of diarrhoea quantifies the association between disease incidence and mortality. The CFR among children younger than 5 years decreases non-linearly with SDI, a composite measure of fertility, education, and income (figure 2). The highest CFR among children younger than 5 years in 2016 occurred in Lesotho (0·16%, 95% UI 0·12–0·20%). Other high CFRs occurred in western sub-Saharan Africa including Sierra Leone (0·15%, 0·12–0·19) and Mali (0·15%, 0·12–0·19; figure 2). Countries in southern sub-Saharan Africa, including Lesotho, Botswana, and South Africa, had higher CFRs than expected based on the SDI alone, perhaps due to the high HIV burden in these regions (figure 2). Conversely, Palestinian territory had a much lower CFR (<0·001%) than expected based on SDI, joined by numerous countries in southeast Asia such as Vietnam, Cambodia, and Sri Lanka (figure 2). The global CFR among boys younger than 5 years (0·042%, 95% UI 0·041–0·042%) was also marginally higher than among girls younger than 5 years (0·039%, 0·038–0·039).

Rotavirus was the leading aetiology for diarrhoea mortality among all children younger than 5 years (128 515 deaths, 95% UI 105 138–155 133) and among all ages (228 047 deaths, 183 526–292 737) at the global level (table 2). Among estimated causes, *Clostridium difficile* was responsible for the fewest deaths in children younger than 5 years globally (1958 deaths, 1458–2623), but was responsible for the most deaths among children younger than 5 years (138 deaths, 111–169) and among all ages (7761 deaths, 6874–8703) in high SDI countries. Global diarrhoea mortality among individuals older than 5 years was dominated by shigella. Of the 212 438 deaths (136 979–326 913) attributable to shigella in 2016, nearly 70% occurred in individuals older than 5 years (table 2). *Vibrio cholerae* (cholera) was the third leading cause of diarrhoea mortality among all ages, responsible for 107 290 deaths (66 518–180 436).

**Table 2:** Deaths, attributable fraction, and episodes due to each diarrhoea aetiology among all ages, children younger than 5 years, and adults older than 70 years, in 2016 globally

	**Deaths (95% UI)**	**Deaths per 100 000 (95% UI)**	**Millions of episodes (95% UI)**	**Episodes per 1000 (95% UI)**	**Fatal attributable fraction (95% UI)**
**Adenovirus**					
All ages	93 286 (62 645–136 144)	1·3 (0·8–1·8)	165·48 (118·49–222·38)	22·4 (16·0–30·1)	5·68% (4·07–7·71)
Younger than 5 years	52 613 (34 709–74 377)	8·3 (5·5–11·8)	75·27 (46·93–117·80)	119·1 (74·3–186·4)	11·78% (8·19–16·13)
70 years or older	23 872 (13 596–41 598)	5·9 (3·4–10·3)	9·47 (6·30–13·34)	23·4 (15·6–33·0)	3·43% (2·37–4·67)
**Aeromonas**					
All ages	16 881 (5649–38 788)	0·2 (0·1–0·5)	39·33 (17·90–73·78)	5·3 (2·4–10·0)	1·02% (0·34–2·18)
Younger than 5 years	6332 (2098–13 192)	1·0 (0·3–2·1)	16·83 (6·47–39·14)	26·6 (10·2–61·9)	1·42% (0·47–2·92)
70 years or older	7974 (1812–19 666)	2·0 (0·4–4·9)	3·88 (1·49–8·08)	9·6 (3·7–20·0)	1·14% (0·29–2·79)
**Amoebiasis**					
All ages	26 748 (5826–74 570)	0·4 (0·1–1·0)	123·50 (54·69–257·47)	16·7 (7·4–34·8)	1·62% (0·34–4·45)
Younger than 5 years	4567 (568–17 863)	0·7 (0·1–2·8)	21·38 (4·79–63·92)	33·8 (7·6–101·2)	1·02% (0·13–3·99)
70 years or older	9673 (1566–30 389)	2·4 (0·4–7·5)	5·65 (1·97–13·90)	14·0 (4·9–34·4)	1·40% (0·22–4·51)
***Campylobacter* spp**					
All ages	75 135 (44 356–117 056)	1·0 (0·6–1·6)	172·33 (115·98–246·71)	23·3 (15·7–33·4)	4·58% (2·73–6·92)
Younger than 5 years	40 854 (20 397–65 633)	6·5 (3·2–10·4)	88·35 (47·13–162·40)	139·8 (74·6–257·0)	9·15% (4·60–14·62)
70 years or older	17 345 (8166–33 209)	4·3 (2·0–8·2)	7·63 (4·54–12·07)	18·9 (11·2–29·9)	2·50% (1·31–4·30)
***Vibrio cholerae***					
All ages	107 290 (66 518–180 436)	1·5 (0·9–2·4)	2·88 (2·28–4·04)	0·4 (0·3–0·5)	6·52% (4·02–10·02)
Younger than 5 years	52 232 (31 017–85 372)	8·3 (4·9–13·5)	0·90 (0·64–1·40)	1·4 (1·0–2·2)	11·71% (7·03–18·92)
70 years or older	4741 (1960–10 594)	1·2 (0·5–2·6)	0·19 (0·14–0·27)	0·5 (0·3–0·7)	0·68% (0·31–1·23)
***Clostridium difficile***					
All ages	22 417 (16 946–32 219)	0·3 (0·2–0·4)	0·18 (0·16–0·21)	0·0 (0·0–0·0)	1·36% (1·06–1·74)
Younger than 5 years	1958 (1458–2623)	0·3 (0·2–0·4)	0·02 (0·01–0·02)	0·0 (0·0–0·0)	0·44% (0·33–0·58)
70 years or older	8899 (7105–11 852)	2·2 (1·8–2·9)	0·07 (0·05–0·09)	0·2 (0·1–0·2)	1·31% (0·94–1·80)
***Cryptosporidium* spp**					
All ages	57 203 (29 837–94 748)	0·8 (0·4–1·3)	69·52 (36·09–118·13)	9·4 (4·9–16·0)	3·53% (1·73–6·24)
Younger than 5 years	48 301 (24 612–81 934)	7·6 (3·9–13·0)	44·84 (19·99–88·70)	71·0 (31·6–140·4)	10·83% (5·58–17·81)
70 years or older	1996 (683–4559)	0·5 (0·2–1·1)	0·74 (0·32–1·34)	1·8 (0·8–3·3)	0·29% (0·11–0·59)
**Enteropathogenic *Escherichia coli***					
All ages	12 337 (4439–25 697)	0·2 (0·1–0·3)	14·26 (5·63–30·90)	1·9 (0·8–4·2)	0·76% (0·26–1·62)
Younger than 5 years	9459 (2620–20 094)	1·5 (0·4–3·2)	8·28 (2·29–23·36)	13·1 (3·6–37·0)	2·12% (0·61–4·60)
70 years or older	1464 (513–3471)	0·4 (0·1–0·9)	0·37 (0·16–0·65)	0·9 (0·4–1·6)	0·21% (0·08–0·49)
**Enterotoxigenic *E coli***					
All ages	51 186 (26 757–83 064)	0·7 (0·4–1·1)	222·64 (144·95–322·85)	30·1 (19·6–43·7)	3·10% (1·70–4·63)
Younger than 5 years	18 669 (9800–30 659)	3·0 (1·6–4·9)	75·16 (39·69–130·35)	118·9 (62·8–206·3)	4·19% (2·21–6·85)
70 years or older	18 152 (7957–34 248)	4·5 (2·0–8·5)	15·00 (9·09–22·80)	37·1 (22·5–56·4)	2·61% (1·27–4·12)
**Norovirus**					
All ages	19 496 (8747–38 421)	0·3 (0·1–0·5)	139·63 (47·77–278·41)	18·9 (6·5–37·7)	1·19% (0·52–2·36)
Younger than 5 years	10 629 (5274–19 582)	1·7 (0·8–3·1)	48·56 (19·02–100·87)	76·8 (30·1–159·6)	2·38% (1·22–4·34)
70 years or older	3693 (810–8647)	0·9 (0·2–2·1)	7·60 (2·15–16·26)	18·8 (5·3–40·2)	0·54% (0·14–1·27)
**Non-typhoidal *Salmonella* spp**					
All ages	84 799 (46 201–144 935)	1·1 (0·6–2·0)	197·35 (127·37–299·20)	26·7 (17·2–40·5)	5·17% (2·87–8·48)
Younger than 5 years	37 410 (16 659–64 509)	5·9 (2·6–10·2)	64·00 (31·32–122·03)	101·3 (49·6–193·1)	8·38% (3·88–14·04)
70 years or older	19 056 (8451–35 666)	4·7 (2·1–8·8)	9·77 (5·55–16·14)	24·2 (13·7–39·9)	2·76% (1·37–4·70)
**Rotavirus**					
All ages	228 047 (183 526–292 737)	3·1 (2·5–4·0)	591·73 (496·00–695·46)	80·0 (67·1–94·1)	13·91% (11·51–16·34)
Younger than 5 years	128 515 (105 138–155 133)	20·3 (16·6–24·5)	258·20 (196·95–336·88)	408·6 (311·6–533·1)	28·78% (25·22–32·40)
70 years or older	57 594 (36 869–91 286)	14·2 (9·1–22·6)	29·03 (21·92–37·78)	71·8 (54·2–93·4)	8·31% (6·83–9·91)
**Shigella**					
All ages	212 438 (136 979–326 913)	2·9 (1·9–4·4)	269·19 (176·68–369·00)	36·4 (23·9–49·9)	12·85% (9·01–17·09)
Younger than 5 years	63 713 (41 191–93 611)	10·1 (6·5–14·8)	74·77 (41·40–127·74)	118·3 (65·5–202·1)	14·28% (9·58–20·74)
70 years or older	74 402 (42 443–128 668)	18·4 (10·5–31·8)	16·39 (10·46–23·36)	40·5 (25·9–57·8)	10·73% (7·18–14·81)

UI=uncertainty interval.

Childhood wasting, defined as having a weight-for-height score more than 2 SDs less than the mean, and unsafe water and sanitation were the leading risk factors for diarrhoea mortality. Childhood wasting was responsible for 80·4% (95% UI 68·2–85·0) of diarrhoea deaths and the proportion of deaths attributable to wasting was unchanged between 2006 and 2016 (77·2% in 2006 *vs* 77·0% in 2016). Unsafe water (72·1%, 34·0–91·4) and unsafe sanitation (56·4%, 49·3–62·7) were the second and third leading risk factors among children younger than 5 years, respectively, and were the first (70·1% [32·2–89·5] of diarrhoea deaths) and second (54·2% [47·1–60·4] of diarrhoea deaths) leading risks among all ages, respectively.

**Figure 3:**
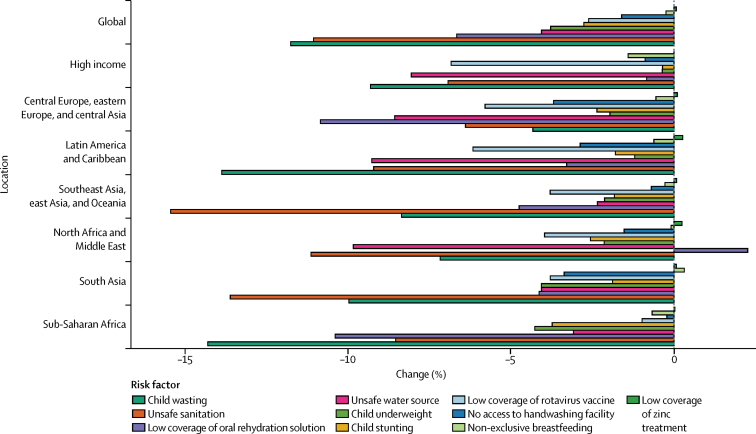
Reduction in under-5 mortality rate due to diarrhoea, 2000–16

Figure 3 shows ten risk factors for diarrhoea mortality among children younger than 5 years and the independent decomposition of each on diarrhoea mortality between 2000 and 2016. Globally, reduction in childhood wasting was responsible for an 11·8% decrease in diarrhoea mortality during this time. Decreasing wasting prevalence was responsible for the largest decrease in diarrhoea mortality in three super-regions, with the largest decrease in sub-Saharan Africa (14·3%) and the smallest in central Europe, eastern Europe, and central Asia (4·3%). Improvements in sanitation were responsible for a 15·4% decrease in southeast Asia, east Asia, and Oceania, and a greater than 10% decrease in diarrhoea mortality globally. The introduction of the rotavirus vaccine has contributed to almost a 7% reduction in mortality of children younger than 5 years due to diarrhoea in the high-income super-region (6·8%) and more than 6% in Latin America and the Caribbean (6·2%).

**Figure 4:**
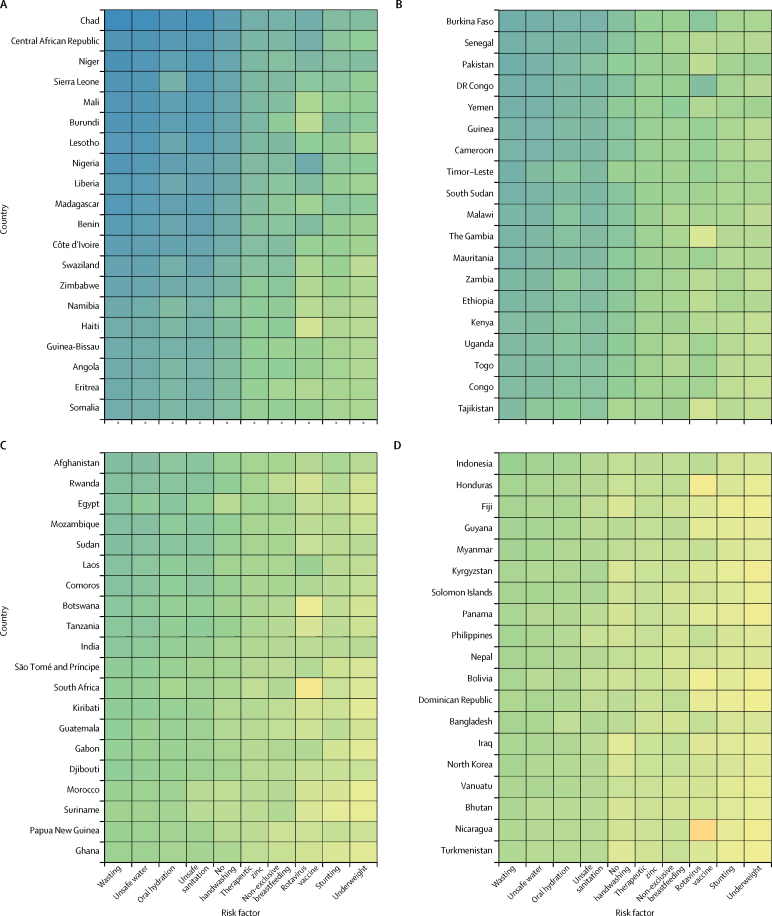

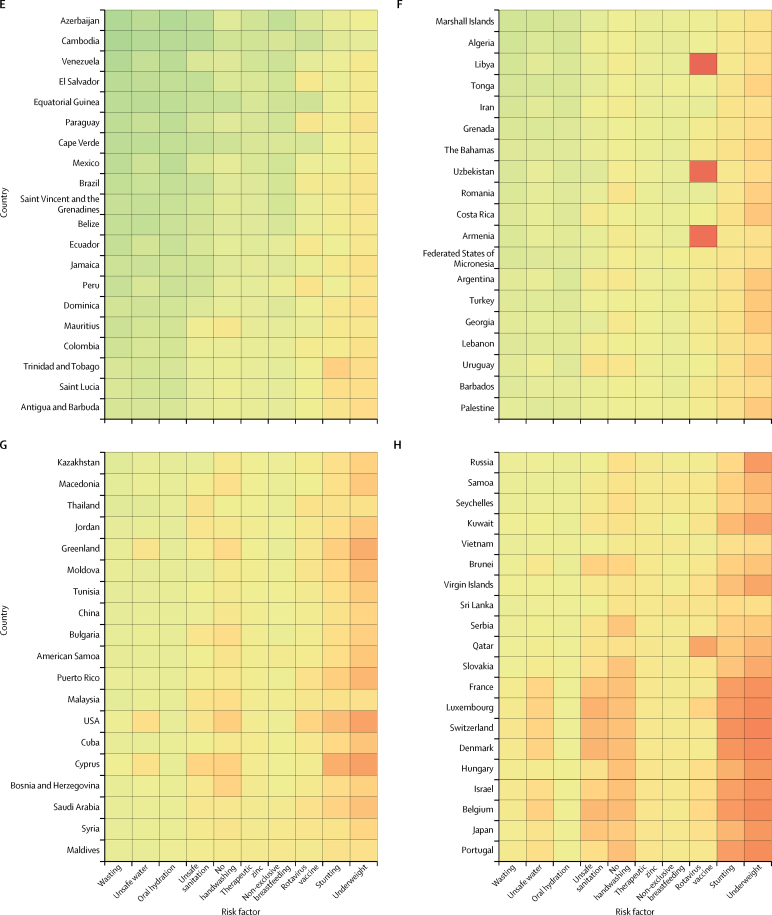

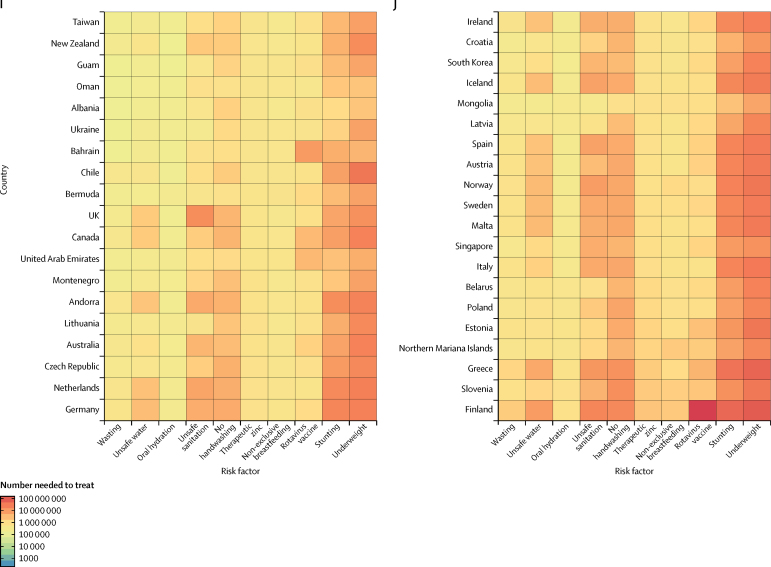
Heat map of the number of children younger than 5 years that were needed to treat to prevent a diarrhoea death in 2016 Countries are ordered by the diarrhoea mortality rate among children younger than 5 years into deciles from Chad (highest mortality rate) to Finland (lowest mortality rate).

By taking the inverse of the under-5 mortality rate in the same ten interventions, we estimated the number of children treated that could prevent one death. Interventions to address childhood wasting require the fewest number of children needed to treat to prevent a diarrhoea death globally and in every super-region other than the high-income super-region. At the global level, prevention of wasting in 1762 children (95% UI 1521–2170) could avert one death from diarrhoea (figure 4). Although interventions to address under-nutrition might be shared across growth indicators, we estimated that direct intervention on underweight and stunting would not be as efficient in the prevention of diarrhoea deaths. Provision of access to safe water (1964 children, 1481–4223) and use of oral rehydration solution (2490, 1748–4171) are globally the second and third most impactful interventions, respectively (figure 4). The relative ranking of efficiency among risk factors and interventions was similar among countries with high and low diarrhoea mortality. Six risk factors (childhood wasting, unsafe water, oral rehydration, unsafe sanitation, handwashing, and therapeutic zinc) need to reach fewer than 3000 children to avert one diarrhoea death in sub-Saharan Africa (figure 4). Results for GBD 2016, including all models, mortality, incidence, and DALYs are published in the Global Health Data Exchange.

## Discussion

Much progress has been made in the reduction of diarrhoea burden among children younger than 5 years; however, diarrhoea remains a leading cause of death and morbidity. The results of this analysis indicate that progress in the reduction of diarrhoea mortality is not equal across locations and regions, which is in part driven by decreases in several primary risk factors, and that the burden in elderly adults is an increasing public health challenge that requires appropriate attention.

Our results suggest that nearly three-quarters of diarrhoea deaths occurred in individuals older than 5 years, with a particularly high burden in adults older than 70 years. Mortality rates in this age group have decreased in most regions of the world since 1990 (42% decrease globally) and yet, as countries have moved through epidemiological transition, the population of adults older than 70 years has increased globally. The number of adults older than 70 years has increased by 50% from 1990 to 2016. Much of the global initiative to reduce the diarrhoea burden has been focused in children younger than 5 years; however, our results suggest that neglect of the burden in adults will have increasingly negative consequences. The high burden of *Clostridium difficile* in high-income countries, particularly in elderly age groups, might explain some of the increasing diarrhoea mortality because this bacterium is difficult to treat, is often resistant to antibiotics, and is frequently associated with nosocomial and retirement home outbreaks.^16^, ^17^ Further exploration of the impact of comorbidities and other risk factors that put elderly people at risk of diarrhoea should be investigated.

Although diarrhoea incidence in children younger than 5 years decreased in most countries during this time, it decreased at a slower rate in most locations than the mortality rate, and it increased in some countries. This finding suggests that the primary drivers of change in diarrhoea mortality have been ones that preferentially reduce the risk of dying from the disease rather than those that reduce the risk of infection. Interventions to prevent diarrhoea mortality should be targeted to the unique characteristics of different countries and regions. Our estimates regarding the number of children that need to be treated to prevent a diarrhoea death facilitate the necessary discussion about targeted intervention implementation. For example, promotion of a handwashing campaign in central Europe, eastern Europe, or central Asia would require reaching at least 60 000 more individuals than would promotion of intervention strategies for wasting, unsafe water, or oral rehydration. Conversely, in sub-Saharan Africa, 500–1000 more individuals would need to be reached through a handwashing campaign than through an intervention for wasting, unsafe water, or oral rehydration. Because the cost of intervention delivery might vary, and sometimes substantially between interventions, the number needed to treat could be an important factor in the assessment of the cost-effectiveness of such interventions.

Childhood undernutrition, stunting, wasting, and underweight were among the leading risks for diarrhoea in children younger than 5 years. Numerous reasons exist as to why many children in low-income countries, born underweight or not, tend to progressively deviate from the global age curves as they get older.^18^ One reason might be a positive reinforcement loop that exists between infectious disease incidence causing poor physical growth, which subsequently predisposes children to future episodes of infectious diseases.^19^, ^20^, ^21^ In fact, diarrhoea burden might be underestimated in this analysis.^18^ Diarrhoea as a comorbidity with other infectious causes of death might contribute to mortality while not being the underlying cause of death. The GBD study assumes that causes of death are mutually exclusive and collectively exhaustive, and that each death is attributable to a single cause. Disentangling the impact of diarrhoea on other causes of death is challenging. Evidence exists to suggest that diarrhoea impairs childhood growth,^19^ and by accounting for the increased risk of subsequent infectious disease episodes due to growth faltering attributable to diarrhoea, the total number of DALYs due to diarrhoea could increase by up to 40% globally.^18^ The breaking of this loop has proved challenging but some countries have succeeded more than others. Beyond socioeconomic development, this success might be due in part to improved maternal education, prenatal care, and interventions that target mothers and children.^22^, ^23^ For example, Angola, Mongolia, and Vietnam have successfully improved childhood nutrition and reduced the burden of diarrhoea.

Our results show that the CFR among children younger than 5 years decreases rapidly with increasing sociodemographic development. We previously showed that diarrhoea mortality decreases much quicker with SDI than diarrhoea incidence, suggesting a strong association exists between factors that relate to high excess mortality due to diarrhoea.^2^ This trend has numerous explanations, including improved access to health care and nutrition, and possibly improved case management. A key goal of the Global Action Plan for the Prevention and Control of Pneumonia and Diarrhoea was universal access to oral rehydration solution and antibiotic use for dysentery.^24^ Our results suggest that expanded use of oral rehydration solution contributed to a decrease in global mortality due to diarrhoea in children younger than 5 years between 2000 and 2016 and was the second largest individual driver of this change in sub-Saharan Africa. Despite modest increases in coverage of oral rehydration solution,^25^ this intervention can still prevent up to 30% of diarrhoea deaths (about 4000 children need to be reached to prevent one diarrhoea death) and expanding its use could be inexpensive and effective.^26^, ^27^

Despite a growing number of countries introducing the rotavirus vaccine, many with support from the Gavi Alliance,^28^ our results suggest that rotavirus is by far the leading aetiology responsible for diarrhoea incidence and mortality in children and adults. We estimated that introduction and expanded use of the rotavirus vaccine was responsible for a 2·6% decrease in mortality of children younger than 5 years due to diarrhoea between 2000 and 2016, and its use prevented nearly 27 000 deaths in 2016. More than 120 000 deaths among children younger than 5 years were due to rotavirus, the fifth most fatal pathogen globally.^1^ These findings should spur advocacy for improved access to the rotavirus vaccine. Shigella was the second leading cause of diarrhoea mortality and responsible for a high attributable fraction among children younger than 5 years (12·9%) and adults older than 70 years (10·7%). In fact, shigella was the leading cause of diarrhoea mortality among those older than 70 years. Although no efficient and inexpensive point-of-care diagnostic exists for diarrhoeal pathogens, WHO guidelines recommend that suspected episodes of shigella-associated diarrhoea are treated with the appropriate antibiotics.^29^ Several shigella vaccine candidates are in development and our results indicate that their use could prevent deaths among young and elderly populations at risk of death caused by diarrhoea.^30^, ^31^ Cholera epidemics continue to inflict a major burden globally, particularly in post-disaster and conflict-devastated locations. Deaths due to cholera are treated as a fatal discontinuity, or catastrophic event, in GBD studies and are added to the total number of diarrhoea deaths after the standard modelling process for mortality. This process helps to capture the epidemic nature of the disease that our models might otherwise miss or statistically smooth over.^1^

GBD 2016 estimates of diarrhoea mortality in children younger than 5 years in 2015 are nearly identical to the estimates produced for GBD 2015 in the same year (485 827, 95% UI 429 412–547 489 *vs* 498 889, 447 450–557 643)^2^ and are approximately 8% lower than those produced by the WHO Department of Evidence, Information and Research and the Maternal and Child Epidemiology Estimation group (525 977; appendix p 46).^32^ The appendix shows a comparison of aetiologies for diarrhoea-attributable mortality among children younger than 5 years by the Child Health Epidemiology Research Group, from which the WHO Department of Evidence, Information and Research and the Maternal and Child Epidemiology Estimation group was developed,^33^ and GBD 2015 estimates for 2010 (p 48). A systematic review of community-based studies on diarrhoea morbidity found an estimated 3·4 episodes of diarrhoea per child-year in 1990, decreasing to 2·9 in 2010; these estimates are higher than the GBD 2016 estimates (2·1 episodes [95% UI 1·9–2·5] per child-year in 1990, and 1·9 episodes [1·7–2·2] per child-year in 2010).^34^ Although the study was limited to children younger than 2 years, the MAL-ED study found that incidence of community diarrhoea ranged from about 0·5 episodes per child-year in Brazil to 6·2 episodes per child-year in Pakistan.^35^ These data from MAL-ED were included, along with more than 30 000 other datapoints on diarrhoea incidence or prevalence in GBD 2016 (appendix p 14).

Data availability limits our estimates of diarrhoea burden, particularly in regions of the world with the greatest mortality and morbidity. The fraction of deaths in children younger than 5 years that were well coded and the diarrhoea mortality rate in 2016 are inversely associated, with a particular absence of data in Africa, where an analysis for GBD 2016 found that only Egypt had a rating of at least three out of five for completeness and coverage.^1^ Improved surveillance and vital registration systems, including standard reporting mechanisms and case definitions, in sub-Saharan Africa and south and southeast Asia in particular, and additional cohort and clinical trials for the impact of interventions, would substantially reduce several major sources of uncertainty in diarrhoea mortality, aetiological attribution, and risk factor estimates.^36^

Data on diarrhoea incidence and aetiologies among populations older than 5 years are scarce, and studies that investigate the role of diarrhoea among adults, particularly elderly people, would be valuable. A core GBD principle is the inclusion of all reliable data; however, a potential consequence is that because of limited data availability, certain sources with small sample sizes or that are representative of a single site can have large effects on estimates. Data scarcity in high burden locations, particularly sub-Saharan Africa, result in modelled estimates with high uncertainty. For example, inclusion of a verbal autopsy study^37^ in a Nairobi slum had a large impact on diarrhoea mortality in Kenyan adults, a study that might not be representative of a country without many other available cause-of-death data. Furthermore, because of a lack of data to calculate ORs for diarrhoea and its aetiologies among ages older than 5 years in low-income and middle-income countries, we have assumed that ORs for children aged 1–5 years are the same as for older age groups. Although the analysis for these ORs are informed by findings from seven countries, it depends on a single study and the results could be strengthened with additional data from other studies or locations. Additionally, ORs tend to be biased away from the null compared with risk ratios; therefore, the PAFs used in this analysis might overestimate aetiological attribution. We assume that aetiologies associated with severe episodes of diarrhoea or those that required admission to hospital are a proxy for episodes that cause death, an assumption that requires confirmation, possibly through vaccine probe approaches.

The predictive modelling approaches used in GBD 2016 rely on covariates and shared information across space and time to fill in these data gaps. The risk factors described in this analysis are included as covariates in the diarrhoea mortality modelling, yet the list of risk factors included in this analysis might not be exhaustive; evidence on the exposure to food contamination, low birthweight, and antibiotic use might improve predictive estimates in future analyses. Uncertainty is carried through each step of the diarrhoea modelling process and is represented in uncertainty intervals for the results. A list of all GBD 2016 data sources for each country is published in the Global Health Data Exchange.

The GBD study is updated annually. Since a full analysis is done on a regular basis, the study is readily adaptable to changes in methodology, incorporation of new or previously unidentified data sources, and is timely with estimates of mortality and morbidity due to diarrhoea. Future iterations of GBD studies will benefit from strengthened surveillance systems on diarrhoea morbidity and mortality, on aetiology burden, particularly in adults, and from rigorous intervention evaluations. Work to forecast GBD estimates, including diarrhoeal diseases up to 2040, will provide a strategic framework for investment, and built-in scenarios will allow for the evaluation of potential interventions. Work to estimate diarrhoea burden at fine spatial resolutions, similar to estimates of mortality in children younger than 5 years due to malaria,^38^, ^39^ will provide extremely detailed evidence to guide policy at the local level and direct interventions to where they could cause the most change. This type of public health precision requires strong surveillance systems, sophisticated analytical approaches, and the capacity to act on the results.^40^

Although diarrhoeal disease mortality has decreased substantially in the past three decades, much work is still needed to accelerate the reduction in burden in the most vulnerable populations including undernourished children, people without reliable access to safe water and sanitation, and those without access to appropriate health care. We have shown that primary intervention strategies to reduce diarrhoea incidence and universal access to the rotavirus vaccine and oral rehydration solutions are necessary to continue momentum in the improvement of diarrhoea burden.

For the **Global Health Data Exchange** see http://ghdx.healthdata.org

For **GBD 2016 data sources** see http://ghdx.healthdata.org/gbd-2016/data-input-source

## References

[1] GBD 2016 Causes of Death Collaborators (2017). Global, regional, and national age-sex specific mortality for 264 causes of death, 1980–2016: a systematic analysis for the Global Burden of Disease Study 2016. Lancet.

[2] GBD 2015 Diarrhoeal Diseases Collaborators (2017). Estimates of global, regional, and national morbidity, mortality, and aetiologies of diarrhoeal diseases: a systematic analysis for the Global Burden of Disease Study 2015. Lancet Infect Dis.

[3] Mills A (2014). Health care systems in low- and middle-income countries. N Engl J Med.

[4] Bhutta ZA, Das JK, Walker N (2013). Interventions to address deaths from childhood pneumonia and diarrhoea equitably: what works and at what cost?. Lancet.

[5] Chopra M, Mason E, Borrazzo J (2013). Ending of preventable deaths from pneumonia and diarrhoea: an achievable goal. Lancet.

[6] GBD 2016 Disease and Injury Incidence and Prevalence Collaborators (2017). Global, regional, and national incidence, prevalence, and years lived with disability for 328 diseases and injuries for 195 countries, 1990–2016: a systematic analysis for the Global Burden of Disease Study 2016. Lancet.

[7] GBD 2016 Risk Factors Collaborators (2017). Global, regional, and national comparative risk assessment of 84 behavioural, environmental and occupational, and metabolic risks or clusters of risks, 1990–2016: a systematic analysis for the Global Burden of Disease Study 2016. Lancet.

[8] GBD 2015 Risk Factors Collaborators (2016). Global, regional, and national comparative risk assessment of 79 behavioural, environmental and occupational, and metabolic risks or clusters of risks, 1990–2015: a systematic analysis for the Global Burden of Disease Study 2015. Lancet.

[9] Stevens GA, Alkema L, Black RE (2016). Guidelines for accurate and transparent health estimates reporting: the GATHER statement. Lancet.

[10] Foreman KJ, Lozano R, Lopez AD, Murray CJ (2012). Modeling causes of death: an integrated approach using CODEm. Popul Health Metr.

[11] Miettinen OS (1974). Proportion of disease caused or prevented by a given exposure, trait or intervention. Am J Epidemiol.

[12] Kotloff KL, Nataro JP, Blackwelder WC (2013). Burden and aetiology of diarrhoeal disease in infants and young children in developing countries (the Global Enteric Multicenter Study, GEMS): a prospective, case-control study. Lancet.

[13] Liu J, Platts-Mills JA, Juma J (2016). Use of quantitative molecular diagnostic methods to identify causes of diarrhoea in children: a reanalysis of the GEMS case-control study. Lancet.

[14] Rothman K, Greenland S, Lash T (2008). Modern epidemiology.

[15] Das Gupta P (1993). Standardization and decomposition of rates: a user's manual.

[16] Baur D, Gladstone BP, Burkert F (2017). Effect of antibiotic stewardship on the incidence of infection and colonisation with antibiotic-resistant bacteria and *Clostridium difficile* infection: a systematic review and meta-analysis. Lancet Infect Dis.

[17] Burke KE, Lamont JT (2014). *Clostridium difficile* infection: a worldwide disease. Gut Liver.

[18] Troeger C, Colombara D, Rao P (2018). Global disability-adjusted life-year estimates of long-term health burden and undernutrition attributable to diarrhoeal diseases in children younger than 5 years. Lancet Glob Health.

[19] Colombara DV, Khalil IA-M, Rao PC (2016). Chronic health consequences of acute enteric infections in the developing world. Am J Gastroenterol Suppl.

[20] Salam RA, Das JK, Bhutta ZA (2015). Current issues and priorities in childhood nutrition, growth, and infections. J Nutr.

[21] Ibrahim MK, Zambruni M, Melby CL, Melby PC (2017). Impact of childhood malnutrition on host defense and infection. Clin Microbiol Rev.

[22] Duggan MB (2014). Prevention of childhood malnutrition: immensity of the challenge and variety of strategies. Paediatr Int Child Health.

[23] Akombi BJ, Agho KE, Hall JJ, Wali N, Renzaho AMN, Merom D (2017). Stunting, wasting and underweight in sub-Saharan Africa: a systematic review. Int J Environ Res Public Health.

[24] WHO, UNICEF (2013). Ending preventable child deaths from pneumonia and diarrhoea by 2025: the integrated Global Action Plan for Pneumonia and Diarrhoea (GAPPD). 2013.

[25] Sreeramareddy CT, Low Y-P, Forsberg BC (2017). Slow progress in diarrhea case management in low and middle income countries: evidence from cross-sectional national surveys, 1985–2012. BMC Pediatr.

[26] Wilson SE, Morris SS, Gilbert SS (2013). Scaling up access to oral rehydration solution for diarrhea: Learning from historical experience in low- and high-performing countries. J Glob Health.

[27] Das JK, Salam RA, Bhutta ZA (2014). Global burden of childhood diarrhea and interventions. Curr Opin Infect Dis.

[28] Gavi, The Vaccine Alliance Rotavirus vaccine support. http://www.gavi.org/support/nvs/rotavirus/.

[29] WHO (2005). Guidelines for the control of shigellosis, including epidemics due to *Shigella dysenteriae* type 1.

[30] Walker RI (2015). An assessment of enterotoxigenic *Escherichia* coli and shigella vaccine candidates for infants and children. Vaccine.

[31] PATH DefeatDD: Vaccines. https://www.defeatdd.org/solution/vaccines.

[32] Walker CLF, Rudan I, Liu L (2013). Global burden of childhood pneumonia and diarrhoea. Lancet.

[33] Johns Hopking Bloomberg School of Public health Maternal Child Epidemiology Estimation. https://www.jhsph.edu/research/centers-and-institutes/institute-for-international-programs/current-projects/maternal-child-epidemiology-estimation/.

[34] Fischer Walker CL, Perin J, Aryee MJ, Boschi-Pinto C, Black RE (2012). Diarrhea incidence in low- and middle-income countries in 1990 and 2010: a systematic review. BMC Public Health.

[35] MAL-ED Network Investigators (2014). The MAL-ED study: a multinational and multidisciplinary approach to understand the relationship between enteric pathogens, malnutrition, gut physiology, physical growth, cognitive development, and immune responses in infants and children up to 2 years of age in resource-poor environments. Clin Infect Dis.

[36] Mikkelsen L, Phillips DE, AbouZahr C (2015). A global assessment of civil registration and vital statistics systems: monitoring data quality and progress. Lancet.

[37] African Population and Health Research Center, INDEPTH (2015). Kenya-Nairobi Urban Health and Demographic Surveillance System. http://www.indepth-ishare.org/index.php/catalog/29.

[38] Gething PW, Casey DC, Weiss DJ (2016). Mapping Plasmodium falciparum mortality in Africa between 1990 and 2015. N Engl J Med.

[39] Golding N, Burstein R, Longbottom J (2017). Mapping under-5 and neonatal mortality in Africa, 2000–15: a baseline analysis for the Sustainable Development Goals. Lancet.

[40] Dowell SF, Blazes D, Desmond-Hellmann S (2016). Four steps to precision public health. Nature.

