# Supplementary material for: Estimates of the global, regional, and national morbidity, mortality, and aetiologies of diarrhoea in 195 countries: a systematic analysis for the Global Burden of Disease Study 2016
Source: Lancet Infect Dis. 2018 Nov;18(11):1211–28. doi: 10.1016/S1473-3099(18)30362-1 (PMC6202444; doi:10.1016/S1473-3099(18)30362-1)
Supplement: Supplementary appendix [file mmc1.pdf]

THE LANCET
Infectious Diseases

Supplementary appendix

This appendix formed part of the original submission and has been peer reviewed. We post it as supplied by the authors.

Supplement to: GBD 2016 Diarrhoeal Disease Collaborators. Estimates of the global, regional, and national morbidity, mortality, and aetiologies of diarrhoea in 195 countries: a systematic analysis for the Global Burden of Disease Study 2016. *Lancet Infect Dis* 2018; published online Sept 19. http://dx.doi.org/10.1016/S1473-3099(18)30362-1.

Appendix to Estimates of the global, regional, and national morbidity, mortality, and aetiologies of diarrhoea in 195 countries: a systematic analysis for the Global Burden of Disease Study 2016

This appendix provides methodological detail, supplemental figures, and comprehensive information on input data and data transformation.

Contents

Appendix Tables and Figures 2

Summary of diarrhoea mortality modelling 3

Molecular diagnostic methods 18

Modelled aetiologic proportion 20

Aetiology proportion data 22

Summary of Risk Factors 38

Comparison with GBD 2015 40

Comparison with WHO-MCEE 46

References 49

Supplementary Results 52

Appendix Tables and Figures

Appendix Tables

Appendix Table 1. Cause specific mortality input data sources 5
Appendix Table 2. Covariates in CODEm........ 6
Appendix Table 3. Nonfatal input data used in diarrhoea modeling 14
Appendix Table 4. Summary of covariates used in the diarrhoea DisMod-MR meta-regression model ... 16
Appendix Table 5. Details on the severity levels for diarrhoea in GBD 2016 and the associated disability weight (DW) with that severity........ 17
Appendix Table 6. Summary of diarrhoea aetiology data 23
Appendix Table 7. Number of deaths due to diarrhoea among children under-5 in the year 2015 is shown for the WHO-MCEE group as well as for GBD 2015 and GBD 2016 46
Appendix Table 8. The number of diarrhoea deaths among children under-5 in 2010 by aetiology estimated by CHERG, GBD 2015, and GBD 2016 48

Appendix Figures

Appendix Figure 1. Analytic flowchart for diarrhoeal mortality estimation, including aetiologic attribution 3
Appendix Figure 2. Diarrhoea mortality input data geographic distribution 7
Appendix Figure 3. Analytic flowchart for diarrhoeal morbidity estimation strategy including aetiologic attribution........ 8
Appendix Figure 4. The seasonality adjustment for survey data is shown for Eastern sub-Saharan Africa as an example........ 10
Appendix Figure 5. The seasonality curves for each GBD super region and the GBD regions 11
Appendix Figure 6. Geographic distribution of diarrhoea morbidity modelling 15
Appendix Figure 7. Plotted representation of qPCR cycle threshold (Ct) and the diagnostic discrimination between cases and controls in GEMS (accuracy) 19
Appendix Figure 8. Odds ratios for the diarrhoeal aetiologies from the Global Enteric Multicenter Study 20
Appendix Figure 9. The sensitivity and specificity of the non-molecular diagnostic methods to the molecular-based case definition is shown for each pathogen 21
Appendix Figure 10. Geographic distribution of aetiology data........ 25
Appendix Figure 11. Clustered bar chart of the number of children under 5 needed to treat to prevent a diarrhoea death by super-region in 2016........ 39
Appendix Figure 12. Scatterplot of GBD 2015 and GBD 2016 results by country in 2000 and 2015........ 40
Appendix Figure 13. Under-5 mortality and all ages mortality between 1990 – 2016 between GBD 2015 and GBD 2016. 44
Appendix Figure 14. Aetiologic attribution to diarrhoea deaths among children under 5 years old in 2010 45
Appendix Figure 15. The number of under-5 deaths due to diarrhoea in 2015 by country........ 47

Summary of diarrhoea mortality modelling

Appendix Figure 1. Analytic flowchart for diarrhoeal mortality estimation, including aetiologic attribution

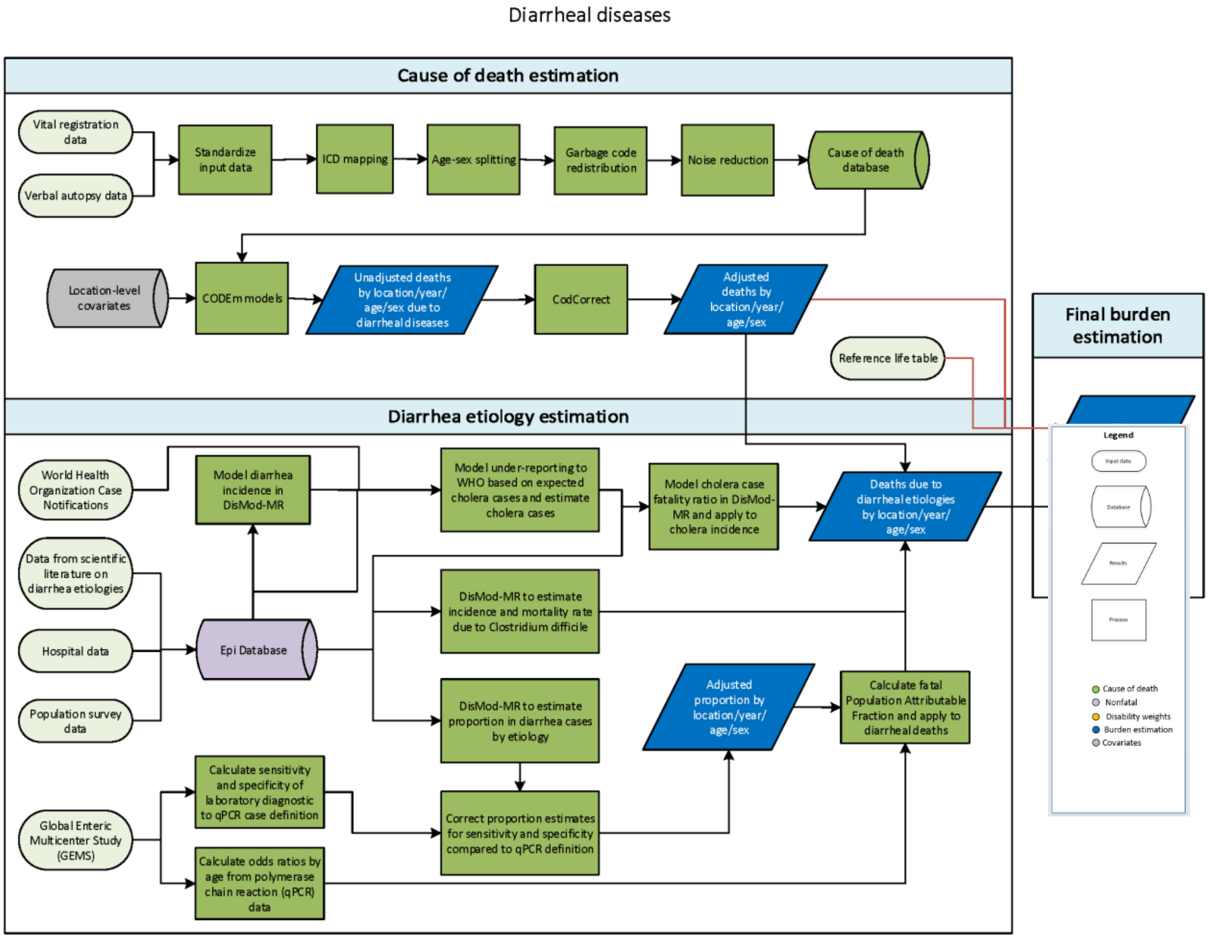

The Cause of Death database for the Global Burden of Disease study has a combination of public and private data from surveillance systems, vital registration systems, and verbal autopsy. To build the database, we first identified verbal autopsy studies, irrespective of cause, by searching PubMed and Google Scholar for all studies with the term "verbal autopsy", and did country-specific searches on Google using the country name and "verbal autopsy". We also identified studies from systematic reviews of diarrhoea mortality and updated them by searching for "verbal autopsy child diarrhoea" and "verbal autopsy child diarrhoea" in Google Scholar. We included studies that used VA, had over 50 deaths, provided the number of deaths due to diarrhoea, and were conducted for at least one year to avoid seasonality. A summary of the input data is shown in **Appendix Table 1** and **Appendix Figure 2.** Diarrhoea mortality was defined by ICD9 and ICD10 codes (ICD9 codes 001-001.9, 003-006.9, 007.4-007.8, 008.01-008.02, 008.04, 008.2-009.9, and 787.91; ICD10 codes: A00-A00.9, A02-A04.1, A04.3, A04.5-A07, A07.2-A07.4, A08-A09.9, and R19.7).

A key component of cause of death modelling in GBD is the redistribution of poorly coded causes of death such as "infection", "fever", or "dehydration" to specific causes of death.[1] This processing of *garbage* codes, causes of death that cannot or should not be considered underlying causes of death,

reallocates a number of deaths into diarrhoeal diseases. The garbage code redistribution was informed by an IHME expert review of the data and subsequent modelling.[1] Garbage code redistribution amount varies by geography and by ICD coding system and is based on a mixed-effects regression of the available cause-of-death data.

For published studies where age groups did not match the GBD age groups, we performed an age-sex split based on the global age distribution of diarrhoea mortality. GBD age groups include: 0-6 days, 7-27 days, 28-364 days, 1-5 years, then in 5-year age groups to age 95. Where necessary, the overall mortality envelope and population estimates by age, sex, and location were used to calculate cause fraction and mortality rates for each data point.

There were 800,310 data points on diarrhoea mortality that were used in the modelling. We also excluded early neonatal mortality data in the Philippines (1994–1998) and India Civil Registration System data for many states (1986–1995). Overall, 14,622 data points were excluded or outliered (2.8% of data points).

Diarrhoeal disease mortality was estimated in the Cause of Death Ensemble model (CODEm) platform.[2,3] CODEm is a Bayesian statistical model and uses spatial priors from a hierarchical structure to inform the mortality models. CODEm is based on five general principles: identifying all available data, maximizing the comparability and quality of the dataset, developing a diverse set of plausible models, assessing the predictive validity of each plausible individual model and of ensemble models, and choosing the model or ensemble model with the best performance in out-of-sample predictive analysis. CODEm produces a large suite of models based on either cause fraction or mortality rate, uses linear and space-time Gaussian process regression (ST-GPR), and a covariate selection process. Each sub-model is evaluated using out-of-sample predictive validity. Thirty percent of the data are excluded from the initial model fits and 15% are used to evaluate component models and 15% used to build the ensembles. The sub-models are ranked using 15% of the data based on their out-of-sample predictive validity. The proportion weighting of the ensemble sub-models is evaluated using the remaining 15% of the hold-out data. This weighting scheme evaluates ensemble models that are built with ranked sub-models contributing proportionally more or fewer draws to the final ensemble. The final ensemble model is evaluated against other ensemble models using the same fit statistics (in-sample, out-of-sample root mean squared error and data coverage). Detailed information on this process can be found in Foreman et al 2012[4] and in the GBD 2016 Mortality and Causes of Death manuscript.[5]

Covariates are selected independently for each sub-model and the selection is based on an algorithm that captures plausible relationships between the covariates and diarrhoeal mortality and provides a diversity of plausible models (**Appendix Table 2**). For every covariate, the direction of effect and a level of biologic proximity to diarrhoea mortality was defined. Each model includes all combination of covariates if the direction of effect is along the assumed direction and the coefficient is significant at $p < 0.05$ level. Also, if adding a more distal covariate changes the statistical significance of a more proximal covariate to non-significant or changes the direction of effect, it will be dropped from the set. The reason for this algorithm to give priority and emphasis on covariates that are more causally and proximately related to diarrhoea such as unsafe water, unsafe sanitation and malnutrition rather than more contextual and macro covariates such as education and income per capita. Covariates are modeled estimates as part of the GBD 2016, typically using surveillance systems, surveys, and scientific literature data to predict values for every GBD estimation age/sex/year/geography. Details on the modeling for most covariates can be found in the GBD 2016 Risk Factors manuscript.[6]

Diarrhoea mortality is estimated for 23 age groups, 774 locations, both sexes, and every year from 1980-2016. We estimated diarrhoea mortality separately for males and females and for children under 5 years

and older than 5 years due to expected underlying differences in the risk of mortality between these age groups. Data-rich and data-poor geographic locations were modelled separately and these models were then hybridised for a global model. This was to maintain proper uncertainty in the models where trusted data on causes of death exist. For a detailed description of the input data coverage, completeness, and reliability of the cause of death data in GBD 2016, please refer to the scoring system introduced in the GBD 2016 Mortality Collaborators manuscript.[5]

Like all models of mortality in GBD, diarrhoea mortality models are single-cause, requiring in effect that the sum of all mortality models must be equal to the all-cause mortality envelope. We correct diarrhoea mortality, and other causes of mortality, by re-scaling them according to the uncertainty around the cause-specific mortality rate. This process is called CoDCorrect and is essential to ensure internal consistency among causes of death.

Appendix Table 1. Cause specific mortality input data sources

**Type of data**	**Input data**
**Total data sources**	**16,980 site-years**
Vital registration data	15,087 site-years
Surveillance data	877 site-years
Verbal autopsy data	1,016 site-years

Appendix Table 2. Covariates in CODEm

CODEm uses a covariate selection algorithm and chooses from the covariates listed in the table below. Covariates are selected from this list while considering prior information about the strength of the association and direction of effect between the covariate and diarrhoeal mortality. The level is ranked from 1 (in causal pathway) to 3 (likely related to diarrhoea mortality). The direction of the relationship is indicated by +, -, and 0 where 0 indicates that a covariate can have either direction of effect in the model.

Covariate	Level	Direction
Diarrhoea Summary Exposure Variable (SEV)*	1	+
Height for age <2 SD	1	+
Sanitation SEV*	1	+
Water SEV*	1	+
Weight for age <2 SD	1	+
Weight for height <2 SD	1	+
Handwashing	1	-
Rotavirus vaccine	1	-
Safe sanitation	1	-
Safe water	1	-
Breastfeeding SEV*	2	+
Vitamin A deficiency	2	+
Zinc deficiency	2	+
Healthcare access and quality index	2	-
LDI per Capita	3	-
Maternal education per capita	3	-
Socio-demographic index	3	-
Population <150/km2	3	0
Population >1000/km2	3	0

*****Summary exposure variables are a risk-weighted prevalence of exposure, scaled so that 1 is 100% of the population exposed and 0 is 0%.[7] Categorical definitions of exposure to a risk factor are weighted based on the relative risk of diarrhoea based on that exposure. For example, the Sanitation SEV is a composite measure of several indicators of unsafe sanitation, such as access to piped waste removal, access to a shared pit latrine, and open defecation. Each of these indicators has a relative risk for diarrhoea and the prevalence of the population using each indicator is weighted by that relative risk and summed across exposure categories and scaled from 0-1.

Appendix Figure 2. Diarrhoea mortality input data geographic distribution

The number of location-years of verbal autopsy or vital registration data for all ages and from 1980-2016 are shown. Countries in dark gray have no data.

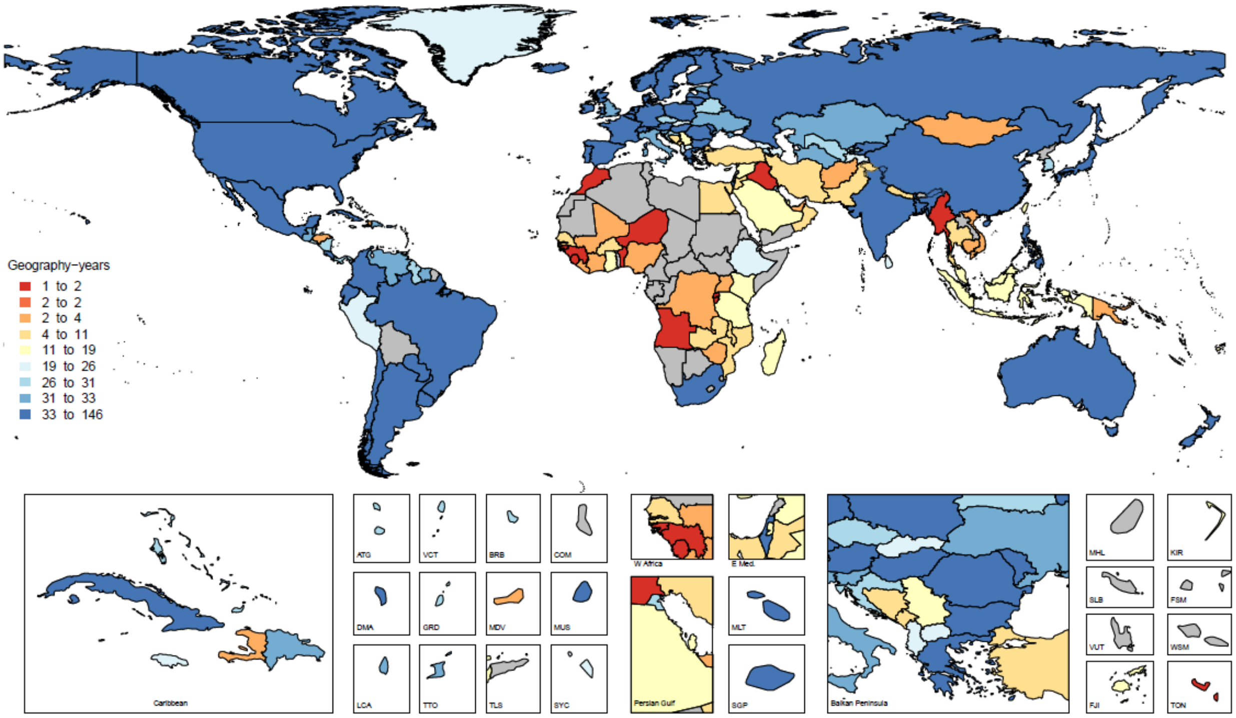

Summary of diarrhoea morbidity modelling

Appendix Figure 3. Analytic flowchart for diarrhoeal morbidity estimation strategy including aetiologic attribution

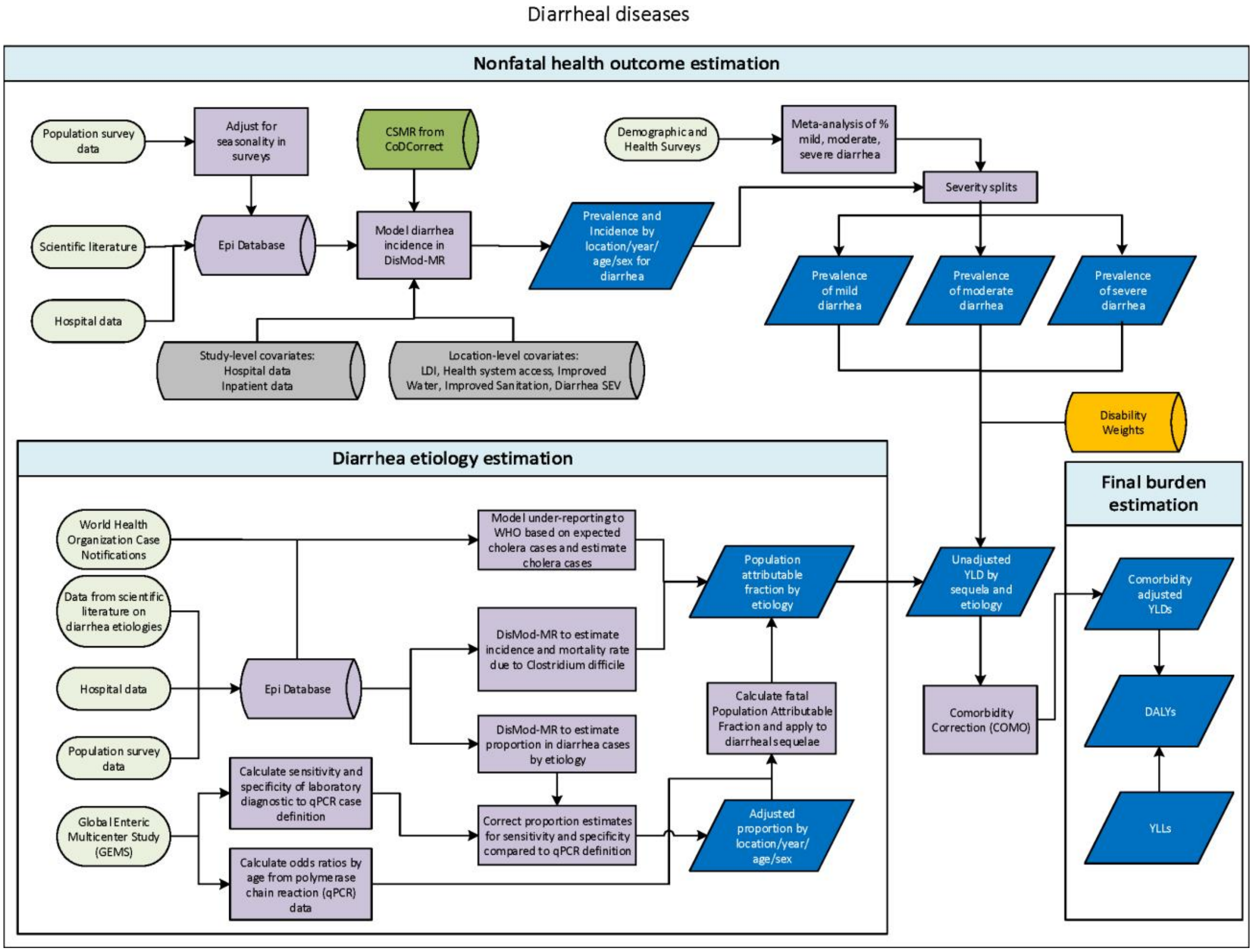

Diarrhoea morbidity was modelled in the DisMod-MR 2.1 platform.[8] DisMod is a Bayesian, hierarchical, meta-regression tool that relates incidence, prevalence, recovery, and mortality in a compartmental model of disease progression. We set the average duration of illness at 4.3 (4.2-4.4) days in the model. Input data are from population representative surveys such as the Demographic and Health Survey (DHS), hospital inpatient and outpatient data (ICD9 codes 001-009.9 and ICD10 codes A00-A09), and from the scientific literature. Input data include all data used in GBD 2015 and a new review of data sources from 2012-August 2016. A summary of the input data is provided in **Appendix Table 3** and **Appendix Figure 6** and the PubMed search string is also listed (**Search strings**). Diarrhoea incidence and prevalence data were extracted concurrently with the aetiology proportion data described on Appendix pages 16-19.

Diarrhoeal disease episodes are characterised as three or more loose stools in a 24 hour period. The reference category for our input data is community based diarrhoea episodes such as data from population-representative surveys or community cohorts. Input data that are from a different population, such as hospital outpatient or inpatient groups, are adjusted by study-level covariates so that they are consistent with the reference category. This step occurs in DisMod.

Data from population-representative surveys, such as the Demographic and Health Surveys and the Multiple Indicator Cluster Surveys, were used and identified using the Global Health Database (GHDx: www.ghdx.healthdata.org). DisMod prevalence input data must be point prevalence. Maternal reported 2-week period prevalence from the surveys was converted to point prevalence in 1-year age increments. Period prevalence was converted to point prevalence using the following formula:

$$p_{point} = p_{period} * \frac{d}{d-1+r}$$

Where *d* is duration in days and *r* is the recall period in days. As surveys are frequently conducted over a period of less than a year, these data are potentially biased by the seasonality of diarrhoea. To account for this variation, we implemented an adjustment factor for survey data where a sine-cosine regression with a period of 6 months was fit to each GBD region, weighted by the standard error of the input data.

$$Prevalence = sine\left(\frac{Prevalence * \pi}{6}\right) + cosine(\frac{Prevalence * \pi}{6})$$

The percent difference in the periodic regression fit and the mean diarrhoea prevalence in each region was used as a scalar to adjust the survey prevalence input data based on the month of the survey for each individual (**Appendix Figure 4**). After applying this scalar, the mean diarrhoea 2-week prevalence from each survey by 1-year age group and by child sex was extracted and prepared for use in the diarrhoea non-fatal modelling.

The seasonality for each GBD region is shown in the **Appendix Figure 5** that follow.

Appendix Figure 4. The seasonality adjustment for survey data is shown for Eastern sub-Saharan Africa as an example

Each point represents a single survey-month diarrhoea prevalence. The solid line shows the sine-cosine fit to the data, adjusting for the year of the survey. The dashed horitzontal line indicates the sine-cosine mean and the relative difference in the solid line compared to the dashed line for each month represents the seasonality adjustment value.

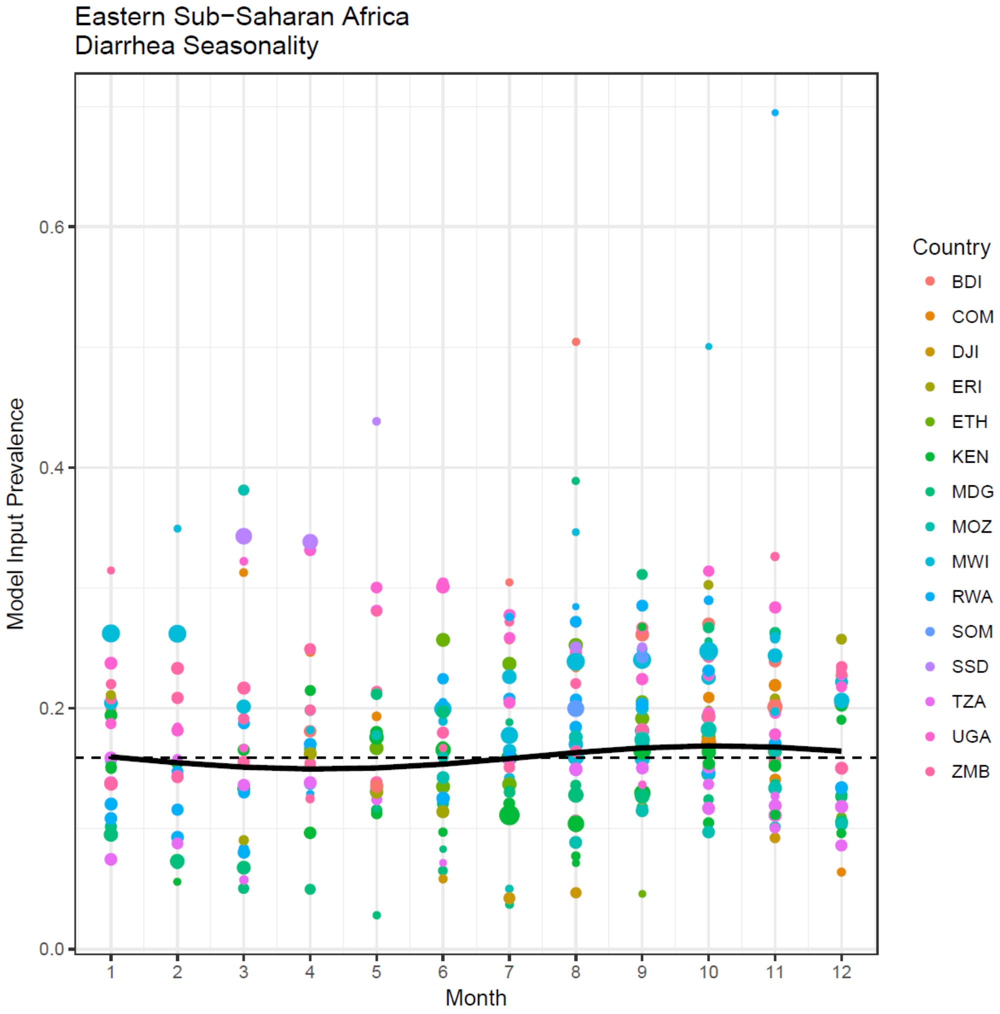

Appendix Figure 5. The seasonality curves for each GBD super region and the GBD regions

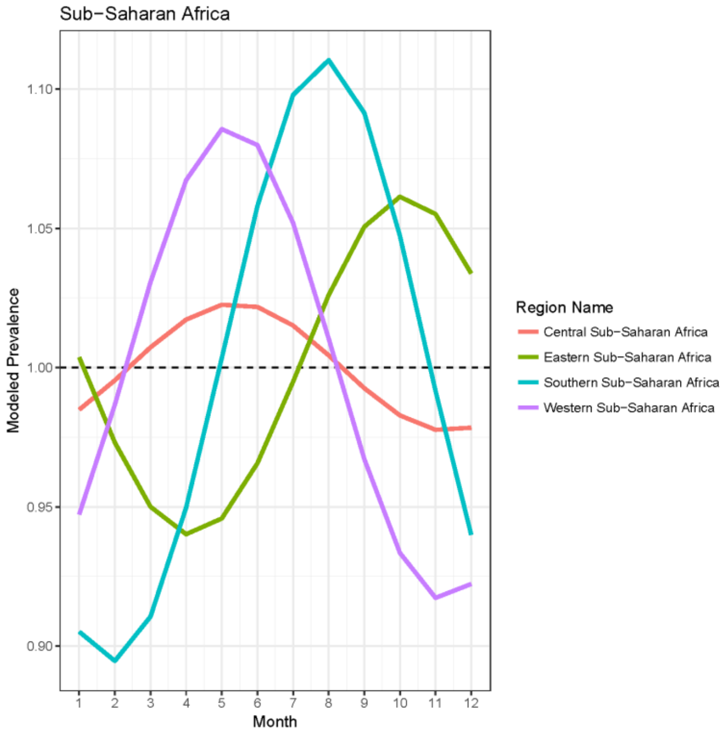

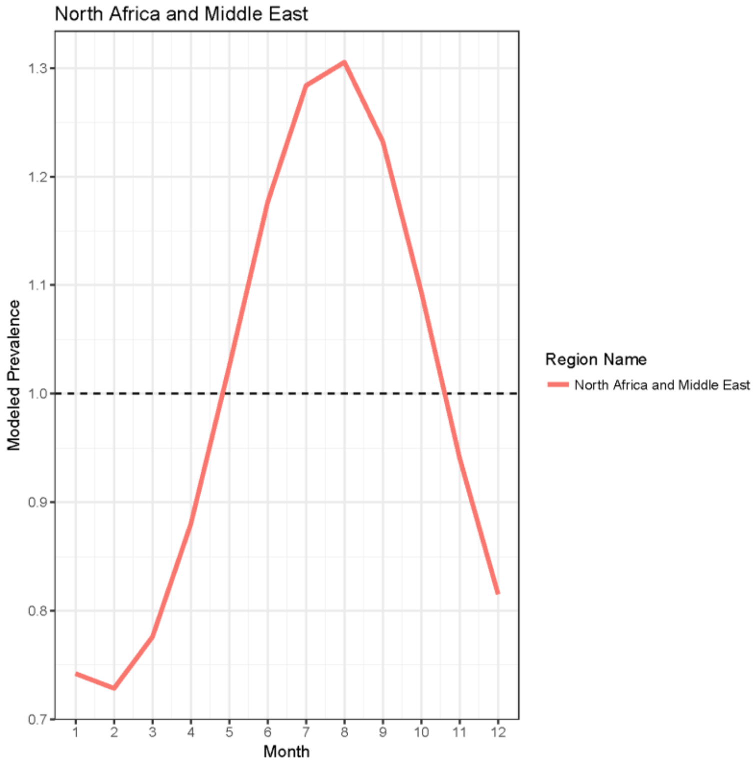

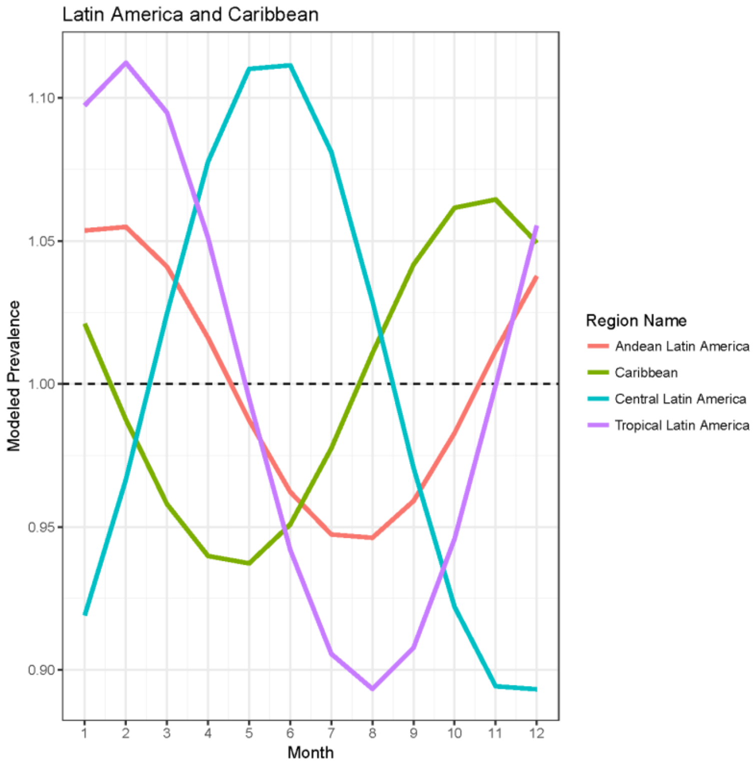
Latin America and Caribbean
1.10
1.05
1.00
0.95
0.90
Modeled Prevalence
1
2
3
4
5
6
7
8
9
10
11
12
Month
Region Name
Andean Latin America
Caribbean
Central Latin America
Tropical Latin America

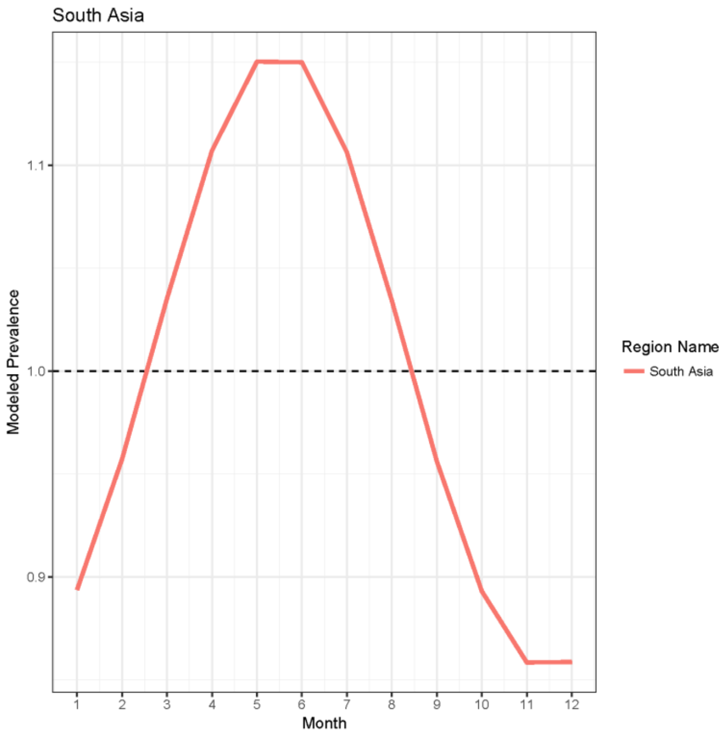
South Asia
1.1
1.0
0.9
Modeled Prevalence
1
2
3
4
5
6
7
8
9
10
11
12
Month
Region Name
South Asia

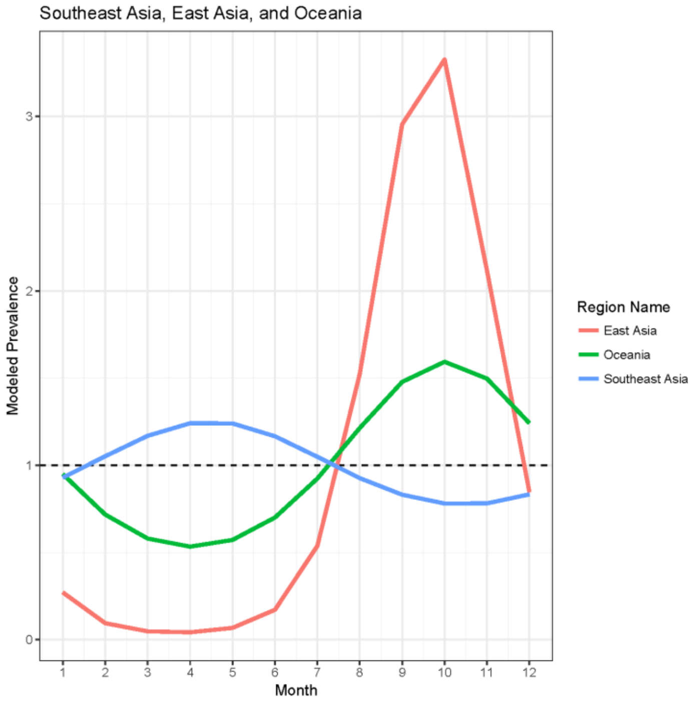
Southeast Asia, East Asia, and Oceania
Modeled Prevalence
0
1
2
3
1
2
3
4
5
6
7
8
9
10
11
12
Month
Region Name
East Asia
Oceania
Southeast Asia

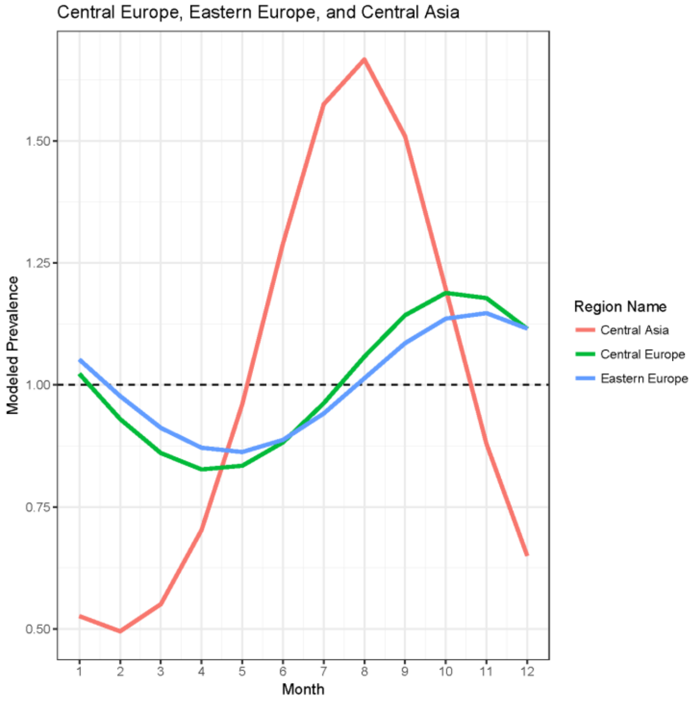
Central Europe, Eastern Europe, and Central Asia
Modeled Prevalence
0.50
0.75
1.00
1.25
1.50
1
2
3
4
5
6
7
8
9
10
11
12
Month
Region Name
Central Asia
Central Europe
Eastern Europe

Hospital data and healthcare utilization (MarketScan, United States only) data were identified using the ICD9 codes 001-009.9 and ICD10 codes A00-A09. To be consistent with the survey data, we transformed the hospital and MarketScan data from incidence to prevalence using an average duration of 4.3 days. Mortality rates from the final Cause of Death model are also used in DisMod as excess mortality rates, a ratio of mortality to prevalence.

Country-level covariates also inform the model. These include the proportion of the population that have access to improved sanitation, access to improved water sources, healthcare access and quality index (a measure of potentially avertable mortality given appropriate medical care),[9] income per capita, and the summary exposure variable (SEV) for diarrhoea (**Appendix Table 4**). The diarrhoea SEV is the sum of risk-weighted prevalence of exposure for each of the risk factors associated with diarrhoea.[7] The risk factors for diarrhoea in the GBD are unsafe water and sanitation, no handwashing with soap, childhood malnutrition, vitamin and zinc deficiency, and sub-optimal breastfeeding.

Diarrhoeal diseases have three severity levels: mild, moderate, and severe (**Appendix Table 5**). The proportion of diarrhoea cases that are assigned to each comes from a published analysis of Demographic and Health Surveys of the proportion of diarrhoeal cases that sought treatment and of community and hospital based studies on the frequency of moderate and severe dehydration.[10] Mild cases are the proportion of diarrhoea cases that did not seek medical care; moderate cases are the proportion that sought medical care but did not have severe dehydration or seizures; and severe cases are the proportion that sought medical care with severe dehydration or seizures with or without dysentery.[11]

To estimate years lived with disability (YLDs) from diarrhoeal diseases, we applied disability weight estimates for each of the possible disease states for prevalent cases of diarrhoeal diseases and the percent of prevalent cases that fall into each state. In the case of diarrhoeal diseases, we assumed that there were three mutually exclusive disease states – mild, moderate, and severe – with corresponding disability weights of 0.074, 0.188, and 0.247, respectively. Disability weights are values between 0 and 1 where 0 represents perfect health and 1 represents death. The disability weights were separately estimated in the Disability Weights Survey portion of the GBD study and were systematically constructed based on responses from more than 6,000 survey respondents.[12]

Appendix Table 3. Nonfatal input data used in diarrhoea modeling

**Type of data**	**Data points (#)**
Facility - inpatient	14,913
Facility - other/unknown	6,540
Survey - cross-sectional	6,474
Other	2,231
**Total**	**30,158**

Appendix Figure 6. Geographic distribution of diarrhoea morbidity modelling

The number of source-years by country is shown. Overall, there are 30,158 data points from 788 unique sources. Input data and models can be found using the GBD visualization tools at: http://vizhub.healthdata.org/data-visualizations.

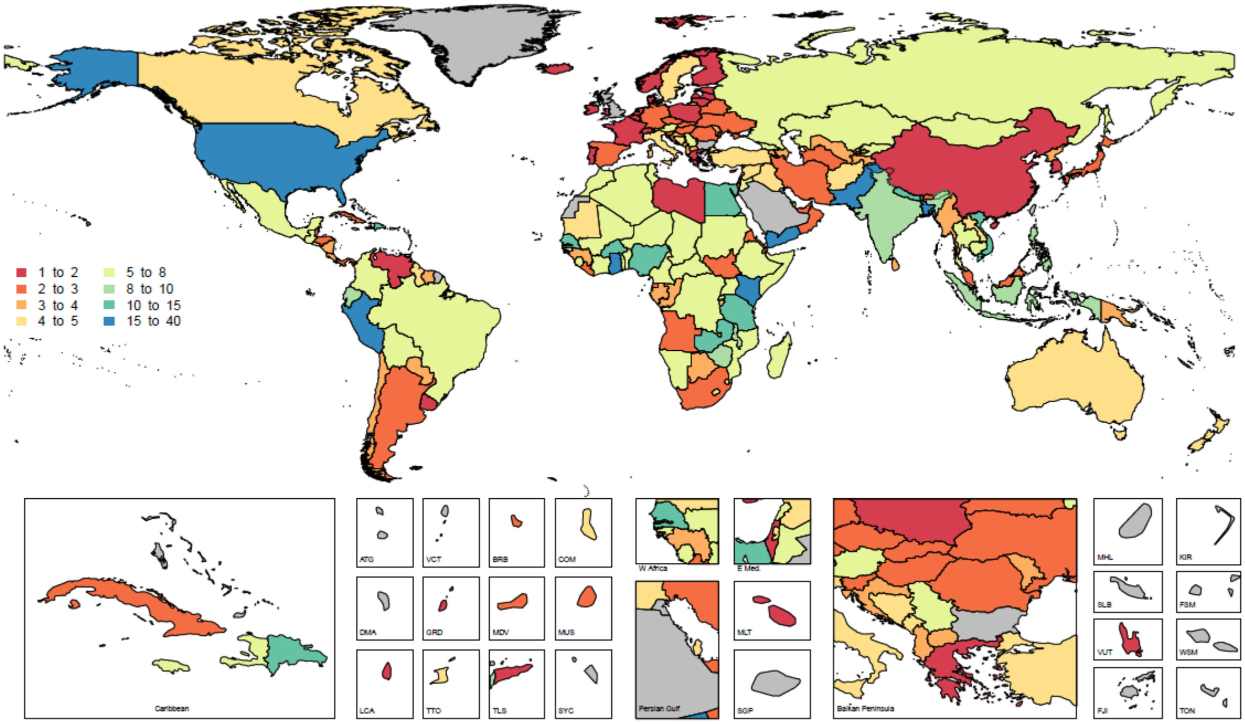

Appendix Table 4. Summary of covariates used in the diarrhoea DisMod-MR meta-regression model

Study-level covariates are binary indicators that are used in DisMod to make data directly comparable from disparate sources. Hospital data are systematically lower than the referent category and so are adjusted upward in the modelling process. Country-level covariates are representative of the country-years used in the model and inform the prediction in areas without data. Numbers in parentheses are 95% uncertainty intervals.

Covariate name	Type of covariate	Parameter	Beta coefficient
Inpatient population	Study	Prevalence	0.0089 (0.0054-0.020)
Healthcare Utilization from Marketscan	Study	Prevalence	0.044 (0.028-0.092)
Hospital data from middle- or low-income country	Study	Prevalence	0.62 (0.24-1.08)
SEV unsafe sanitation	Country	Prevalence	2.36 (2.01-2.64)
Rotavirus vaccine coverage	Country	Prevalence	0.76 (0.74-0.79)
Diarrhoea SEV	Country	Prevalence	0.87 (0.84-0.91)
Healthcare access and quality index	Country	Excess mortality	0.97 (0.97-0.97)

Appendix Table 5. Details on the severity levels for diarrhoea in GBD 2016 and the associated disability weight (DW) with that severity

Numbers in parentheses are 95% confidence intervals.

**Severity level**	**Lay description**	**Disability Weight (95% CI)**	**Percent of Cases**
Mild	Has diarrhoea defined as 3 or more loose stools in a 24 hour period with no dehydration	0.074 (0.049-0.104)	64.2%
Moderate	Has diarrhoea defined as 3 or more loose stools in a 24 hour period and sought medical treatment without dehydration	0.188 (0.125-0.264)	28.9%
Severe	Has diarrhoea defined as 3 or more loose stools in a 24 hour period and sought medical treatment with dehydration or dysentery	0.247 (0.164-0.348)	6.9%

Summary of aetiology population attributable fraction strategy

We estimated diarrhoeal disease aetiologies separately from overall diarrhoea mortality and morbidity using a counterfactual strategy for enteric adenovirus, *Aeromonas*, *Entamoeba histolytica* (amoebiasis), *Campylobacter enteritis*, cryptosporidiosis, typical enteropathogenic *Escherichia coli* (t-EPEC), enterotoxigenic *Escherichia coli* (ETEC), norovirus, non-typhoidal salmonella infections, rotavirus, and *Shigella*. *Vibrio cholerae* and *Clostridium difficile* were modelled separately.

For all aetiologies except *V cholerae* and *C difficile*, the population attributable fraction (PAF) was calculated from the proportion of diarrhoea cases that are positive for each aetiology and the odds ratio of diarrhoea given the detection of that aetiology. This is a counterfactual approach, meaning that the PAF represents the relative reduction in diarrhoea episodes and deaths if there was no exposure to a given aetiology. As diarrhoea can be caused by multiple pathogens and the pathogens may co-infect, PAFs can overlap and add up to more than 100%.

We used the following formula to estimate PAF:[13]

$$PAF = Proportion * (1 - \frac{1}{OR})$$

Where *Proportion* is the proportion of diarrhoea cases positive for an aetiology and *OR* is the odds ratio of diarrhoea given the presence of the pathogen. Both of these values are described in detail below. The number of diarrhoea deaths or episodes due to each aetiology is the product of the PAF for that aetiology and the total number of deaths or episodes due to diarrhoea in that population.

Molecular diagnostic methods

For GBD 2016, we used a systematic reanalysis of the Global Enteric Multicenter Study (GEMS) that uses quantitative polymerase chain reaction (qPCR) as the diagnostic tool for pathogen detection to estimate the odds ratios of diarrhoea given pathogen detection. Validation studies have shown that this approach is more sensitive than traditional laboratory diagnostic methods in detecting diarrhoeal pathogens.[14,15]

The qPCR test results are a continuous variable corresponding to the relative quantity of genetic target in the sample. To be consistent with a binary presence/absence of pathogen case definition from the literature review, we dichotomised the continuous qPCR test result using the lowest value of the cycle threshold (Ct) that accurately discriminated between cases and non-cases in GEMS (**Appendix Figure 7A**). We used the lower Ct value that represented the smallest false positive samples (positive in non-diarrhoea samples) when we had multiple Ct values for the cutpoint (**Appendix Figure 7B**). The Ct values range from 0 to 35 cycles representing the relative concentration of the target gene in the stool sample. A low value indicates a higher concentration of the pathogen while a value of 35 indicates the analytic level of detection. Values above 35 are not reproducible due to the stochasticity involved in the physical distribution of the clinical specimen to wells in the array where the singleplex qPCR is performed. The case definition for each pathogen is a Ct value that is below the established cutoff point.

We used a mixed effects conditional logistic regression model, matching for case-control pairs, random effects for GEMS sites, and accounting for all pathogens to calculate the odds ratio by age for each of our etiologies. This means that an odds ratio by age for each aetiology is applied regardless of the year or geographic location (**Appendix Figure 8**).

Appendix Figure 7. Plotted representation of qPCR cycle threshold (Ct) and the diagnostic discrimination between cases and controls in GEMS (accuracy)

A). The relationship between Ct and accuracy is shown for rotavirus, **B).** The relationship between Ct and accuracy is shown for adenovirus. Adenovirus has two inflection points in the smoothed accuracy curve and so the lower of the two points is chosen as the cutoff.

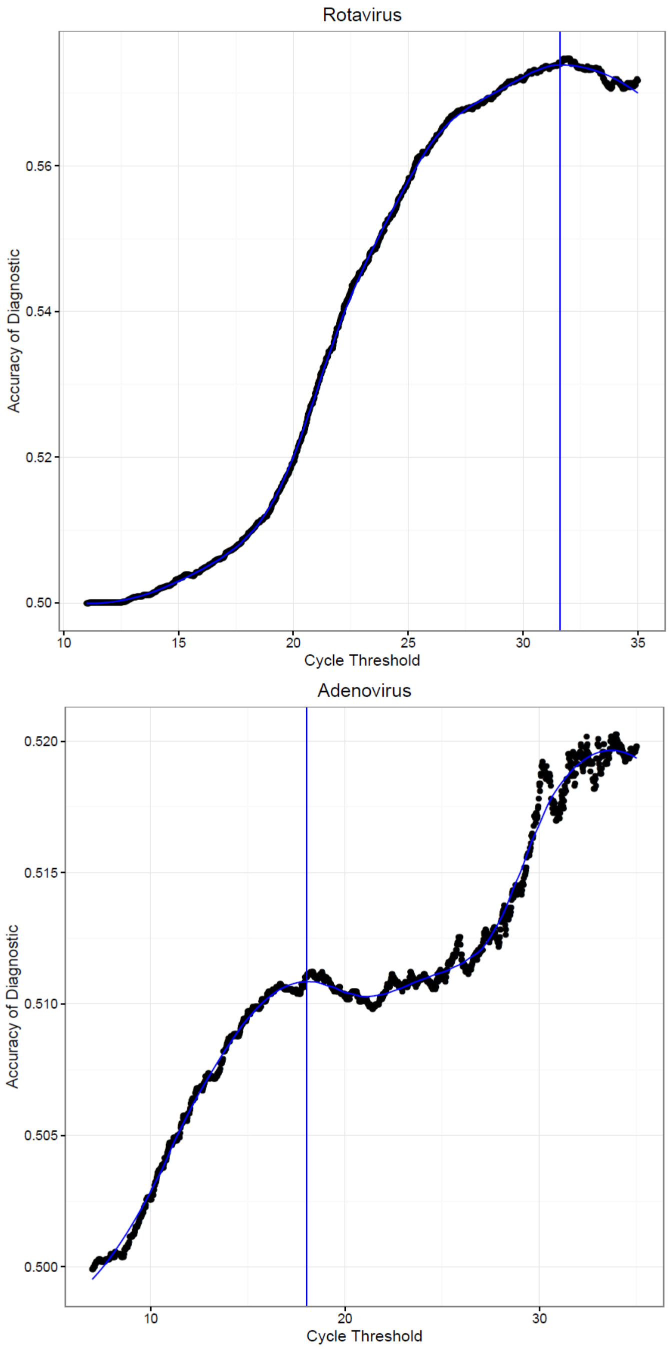

Appendix Figure 8. Odds ratios for the diarrhoeal aetiologies from the Global Enteric Multicenter Study

The panel on the left is among children less than 1 year old and the panel on the right is among children 1-5 years old. The odds ratios in the right panel are applied to all ages older than 1 year in GBD 2016.

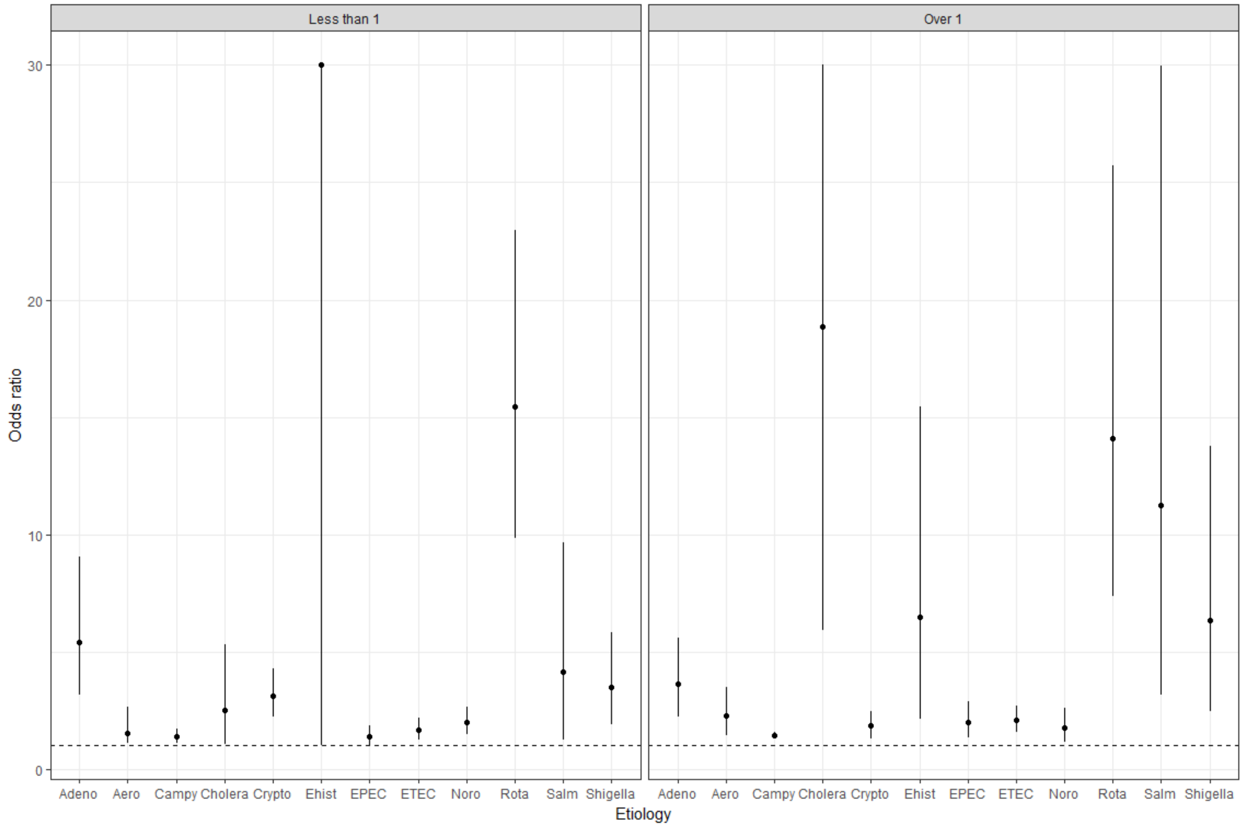

Modelled aetiologic proportion

We updated our systematic literature review to include all data from GBD 2016 and new data identified using the search strings provided below. We extracted data that described the frequency of isolation for each aetiology among diarrhoeal stool samples. We excluded studies with a sampling population fewer than 100 samples, a sampling duration shorter than 1 year to avoid biases in seasonality, and studies that used vomiting as an acceptable case definition. We extracted data at the highest time and age detail possible from a study. If the sampling age was not specified, we assumed it applied to all ages.

We modelled the proportion data using the meta-regression tool DisMod-MR to estimate the proportion of positive diarrhoea cases for each separate aetiology by location/year/age/sex and to adjust for covariates. DisMod adjusts data to be comparable before performing meta-regressions. A binary indicator for if the proportion data come from inpatient populations, assumed to be a proxy for severe and fatal diarrhoea episodes, is used as a scalar to differentiate the relative frequency of detection in fatal and non-fatal diarrhoea episodes for distinct PAFs for fatal and non-fatal diarrhoea episodes. We also accounted

for the frequency of detection in single compared to multi-pathogen studies as the detection is higher in studies that focus on a single pathogen.

We used the estimated sensitivity and specificity of the laboratory diagnostic technique used in the GEMS study compared to the qPCR case definition among cases to adjust our proportion before we computed the PAF:[16,17]

$$Proportion_{True} = \frac{(Proportion_{Observed} + Specificity - 1)}{(Sensitivity + Specificity - 1)}$$

We used this correction to account for the fact that the proportions we used are based on a new test that is not consistent with the laboratory-based case definition (qPCR versus GEMS conventional laboratory testing for pathogens).[18] A summary of the sensitivity and specificity of the non-molecular diagnostics to the molecular case definition for each pathogen is shown in **Appendix Figure 9.**

Appendix Figure 9. The sensitivity and specificity of the non-molecular diagnostic methods to the molecular-based case definition is shown for each pathogen

The sensitivity and specificity values are from diarrhoea cases in the Global Enteric Multicenter Study. The error bars represent the 95% confidence interval based on random-sampling bootstrap of the individual-level data.

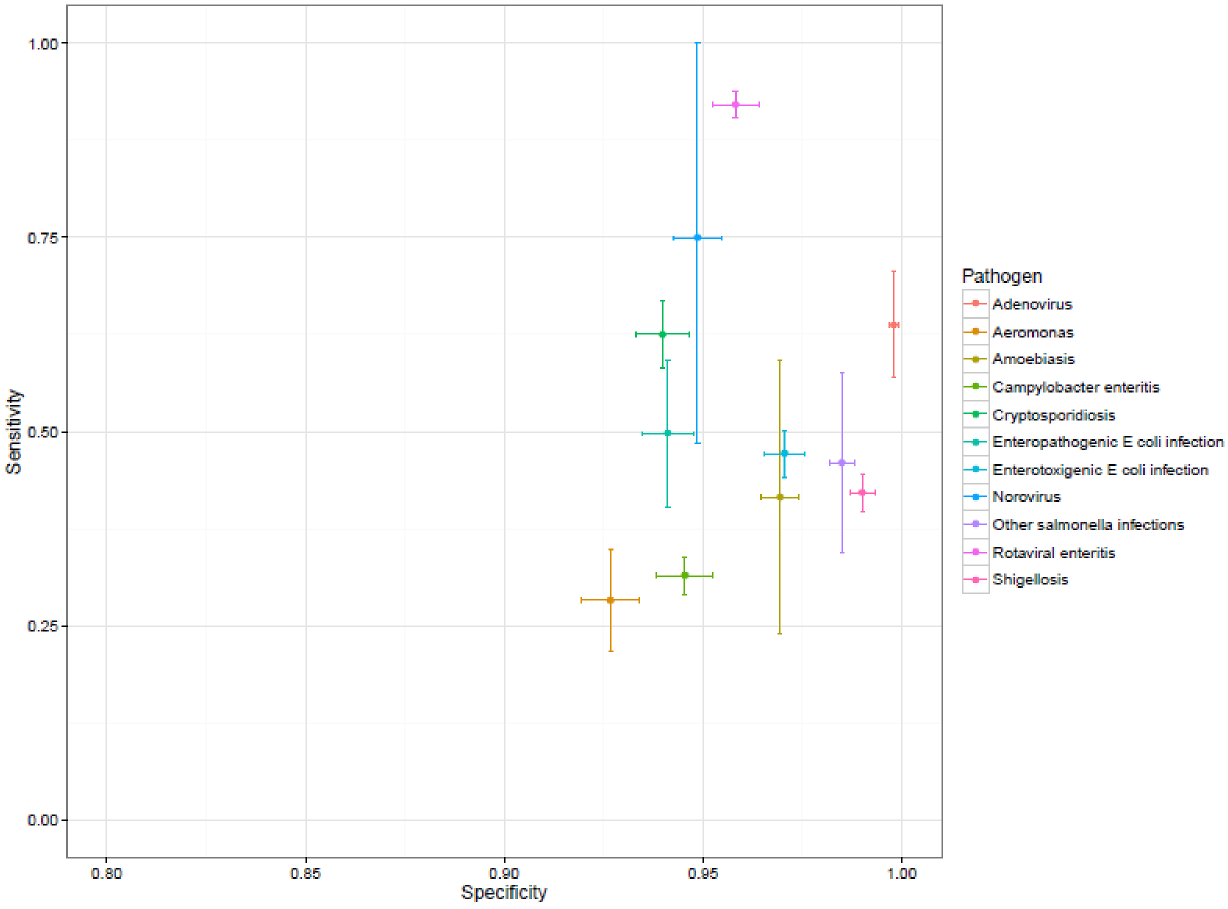

Our literature review extracted the proportion of any enteropathogenic *Escherichia coli* (EPEC) without differentiating between typical (tEPEC) and atypical (aEPEC). In order to be consistent with the odds

ratios that we obtained, which described tEPEC, we adjusted our proportion estimates of any EPEC to typical EPEC only. This adjustment was informed by a subset of our literature review that reported both atypical and typical EPEC. We estimated a ratio, by super-region, of tEPEC to any EPEC and adjusted our proportion estimates accordingly. We found that the majority of EPEC diarrhoea cases were positive for atypical EPEC, consistent with other published work.[19]

For *Vibrio cholerae* (cholera), we used the literature review to estimate expected number of cholera cases for each country-year using the incidence of diarrhoea, estimated using DisMod-MR, and the proportion of diarrhoea cases that are positive for cholera. We assigned cholera PAF using odds ratios from the qPCR results to estimate a number of cholera-attributable cases. We compared this expected number of cholera cases to the number reported to the World Health Organization at the country-year level.[20] We modelled the underreporting fraction to correct the cholera case notification data for all countries using health system access and the diarrhoea SEV scalar to predict total cholera cases. We used the age-specific proportion of positive cholera samples in DisMod and our incidence estimates to predict the number of cholera cases for each age/sex/year/location. Finally, we modelled the case fatality ratio of cholera using DisMod-MR and to estimate the number of cholera deaths.

For *C. difficile*, we modelled incidence and mortality in DisMod-MR for each age, sex, year, location. DisMod-MR is a Bayesian meta-regression tool that uses spatio-temporal information as priors to estimate prevalence, incidence, remission, and mortality for *C. difficile* infection. DisMod-MR uses a compartmental model to relate prevalence, incidence, remission, and mortality. We set remission in our model to 1 month.

Aetiology proportion data

The proportion of diarrhoea episodes where each aetiology is detected is extracted from a systematic literature review. Inclusion criteria are sample population greater than 100 individuals, studies lasting longer than 1-year in duration, and from non-epidemic locations. We excluded studies that reported on diarrhoeal outbreaks and those that used acute gastroenteritis with or without diarrhoea as the case definition. We did not set language restrictions to the search criteria.

For GBD 2016, we updated our review of literature to include studies published between January 2012 and May 2016 (**Appendix Table 6**). The PubMed search strings are provided below. We identified 2,847 studies, of which 152 met our criteria of inclusion and were included. We extracted data points for location, sex, year, and age. The geographic distribution of aetiology data points is shown in **Appendix Figure 10**. We assigned an age range based on the prevalence-weighted mean age of diarrhoea in the appropriate year/sex/location if the age of the study participants was not reported.

We modelled *Vibrio cholerae* independently from the other aetiologies because of its epidemic tendency. We conducted a systematic review of literature for studies published between January 1980 and June 2016 that reported the proportion of diarrhoea cases that tested positive for cholera or the case fatality of cholera (**Search string 2**). We excluded studies specifically about outbreaks and with less than one year of follow-up.

We also modelled *Clostridium difficile* independently from the aetiologies because it was not included as a pathogen in GEMS. We conducted a systematic literature review for the prevalence and incidence of *C. difficile* between January 1990 and May 2016 (**Search string 3**). We used inpatient and outpatient hospital visits coded for *C. difficile* as our incidence data. However, nearly all of the hospital data came from Western countries (**Appendix Figure 10**).

Appendix Table 6. Summary of diarrhoea aetiology data

The number and a description of the data and data types that are used in the diarrhoea aetiologic attribution modelling is shown for each aetiology. Each data source has one or more data point depending on the number of year, age-, and sex-specific values are reported.

Aetiology	Data points	Points among children under-5	Inpatient sample population	New to GBD 2016
Adenovirus	578	415	261	1
Aeromonas	299	193	127	0
Amoebiasis	333	220	132	0
Campylobacter enteritis	670	381	53	98
Cholera	2028	245	43	43
Clostridium difficile	3061	NA	2843	NA
Cryptosporidiosis	535	314	219	129
Enteropathogenic E coli infection	445	315	212	73
Enterotoxigenic E coli infection	400	289	169	82
Norovirus	628	415	212	0
Rotaviral enteritis	2099	1593	1209	205
Other salmonella infections	911	362	232	127
Shigellosis	783	476	315	89

Diarrhoea literature review search strings. Search terms used in PubMed systematic literature review for GBD 2016. Search string 1 is for diarrhoea and all aetiologies except cholera and *Clostridium* and are an update from 2015-2016.

1. ***Diarrhoea and aetiologies:*** *(diarrhoea[title] OR diarrhoea[MeSH Terms] OR diarrhoea[title] OR diarrhoea[MeSH Terms] OR gastroenteritis[title] OR gastroenteritis[MeSH Terms] OR gastro-enteritis[title] OR salmonella[title/abstract] OR shigell*[title/abstract] OR "enteropathogenic e. coli" [title/abstract] OR enterotoxigenic e. coli[title/abstract] OR campylobacter[title/abstract] OR amoebiasis[title/abstract] OR entamoeb*[title/abstract] OR amoebiasis[title/abstract] OR amebiasis[title/abstract] OR cryptosporidi*[title/abstract] OR rotavirus[title/abstract] OR norovirus[title/abstract] OR adenovirus[title/abstract]) AND ((aetiolog*[title/abstract] OR aetiology[MeSH Terms] OR cause[title/abstract] OR pathogen[title/abstract]))* ***NOT*** *((colitis[title/abstract] OR enterocolitis[title/abstract] OR inflammatory bowel[title/abstract] OR irritable[title/abstract]OR Crohn*[title/abstract] OR HIV[title] OR treatment[title] OR therapy[title])) NOT ((appendicitis[title/abstract] OR esophag*[title/abstract] OR surger*[title/abstract] OR gastritis[title/abstract] OR liver[title/abstract] OR case report[title] OR case-report[title] OR therapy[title] OR treatment[title])) AND (("2012/01/01"[PDat] : "2016/12/31"[PDat]) AND Humans[Mesh])*
2. ***Cholera:*** *(((diarrhoea[title] OR diarrhoea[MeSH Terms] OR diarrhoea[title] OR diarrhoea[MeSH Terms] OR gastroenteritis[title] OR gastroenteritis[MeSH Terms] OR gastro-enteritis[title] AND cholera[title/abstract] OR cholera[MeSH Terms]) AND ((aetiolog*[title/abstract] OR aetiology[MeSH Terms] OR cause[title/abstract] OR pathogen[title/abstract])) NOT ((colitis[title/abstract] OR enterocolitis[title/abstract] OR inflammatory bowel[title/abstract] OR irritable[title/abstract]OR Crohn*[title/abstract] OR HIV[title] OR treatment[title] OR therapy[title])) NOT ((appendicitis[title/abstract] OR esophag*[title/abstract] OR surger*[title/abstract] OR gastritis[title/abstract] OR liver[title/abstract] OR case report[title] OR case-report[title] OR therapy[title] OR treatment[title])) AND (("2015/01/01"[PDat] : "2016/12/31"[PDat]) AND Humans[Mesh])))*
3. ***Clostridium:*** *(((diarrhoea[title] OR diarrhoea[MeSH Terms] OR diarrhoea[title] OR diarrhoea[MeSH Terms] OR gastroenteritis[title] OR gastroenteritis[MeSH Terms] OR gastro-enteritis[title] AND clostridium difficile[title/abstract] OR c. difficile[title/abstract] OR c. difficile[Mesh Terms] OR clostridium difficile[Mesh Terms]) AND ((aetiolog*[title/abstract] OR aetiology[MeSH Terms] OR cause[title/abstract] OR pathogen[title/abstract])) NOT ((colitis[title/abstract] OR enterocolitis[title/abstract] OR inflammatory bowel[title/abstract] OR irritable[title/abstract]OR Crohn*[title/abstract] OR HIV[title] OR treatment[title] OR therapy[title])) NOT ((appendicitis[title/abstract] OR esophag*[title/abstract] OR surger*[title/abstract] OR gastritis[title/abstract] OR liver[title/abstract] OR case report[title] OR case-report[title] OR therapy[title] OR treatment[title])) AND (("2015/01/01"[PDat] : "2016/12/31"[PDat]) AND Humans[Mesh])))*

Appendix Figure 10. Geographic distribution of aetiology data

The number of data points that inform aetiologic attribution models by country and by aetiology is shown. Gray indicates no data points.

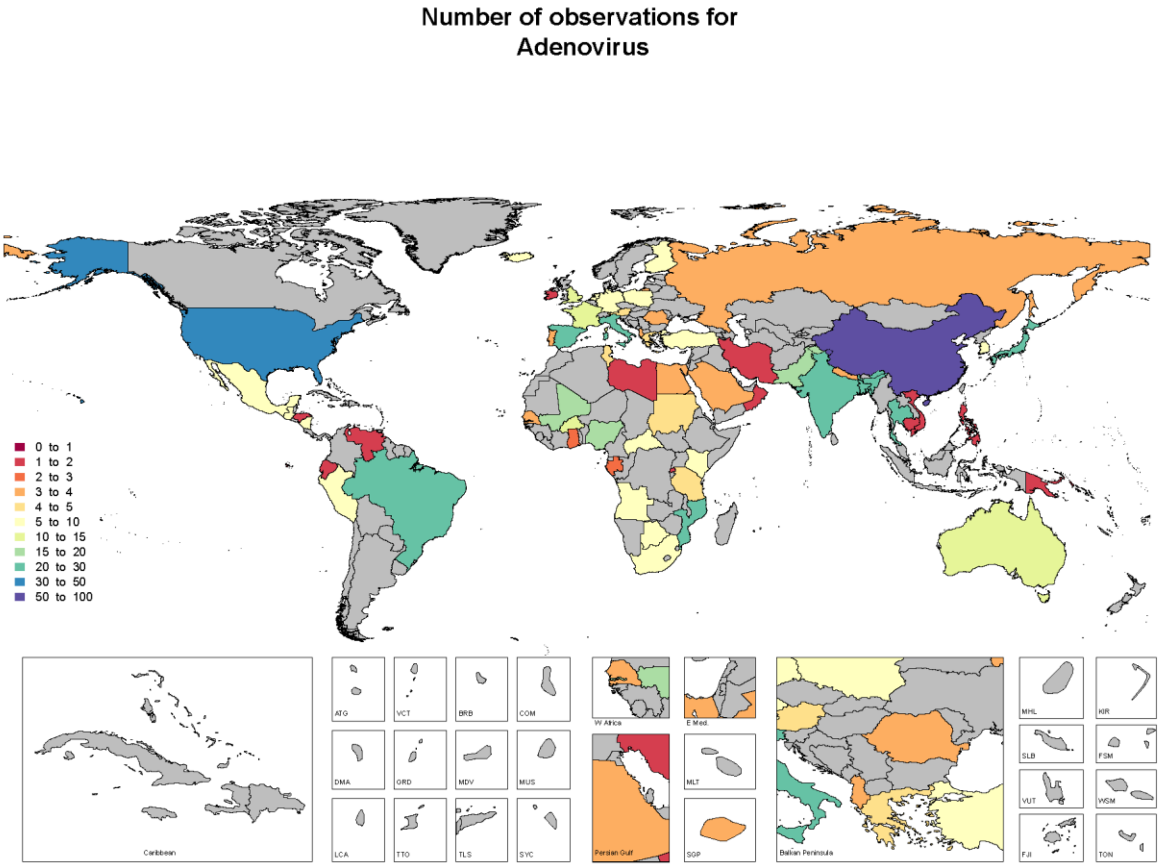

Number of observations for Aeromonas

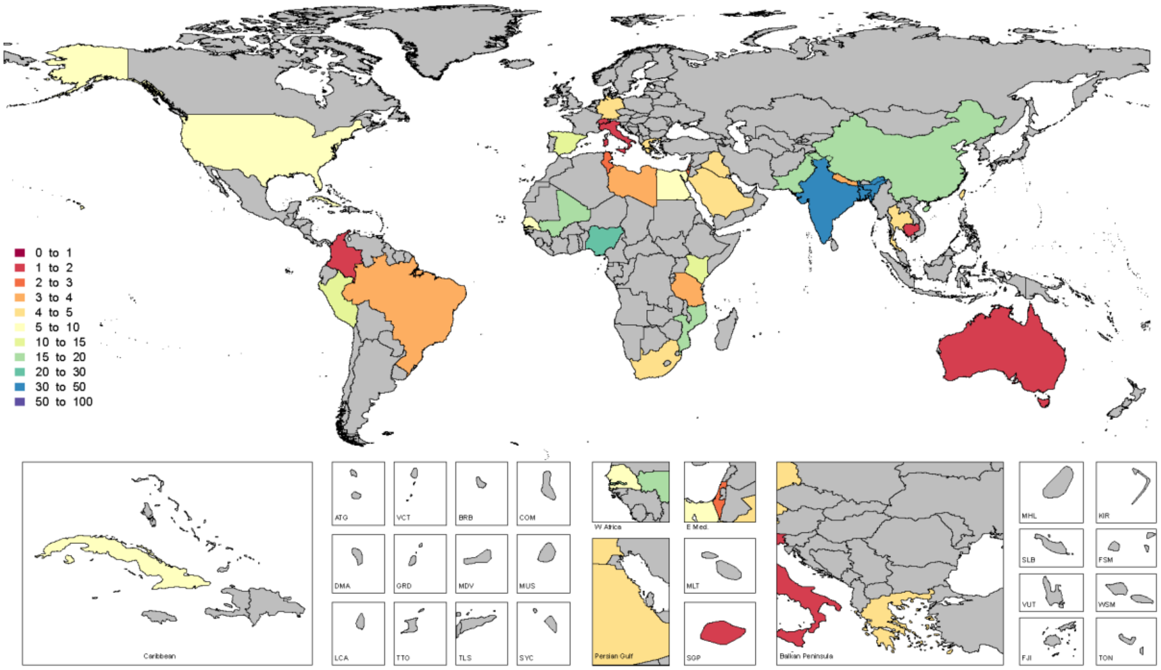

Number of observations for
Entamoeba

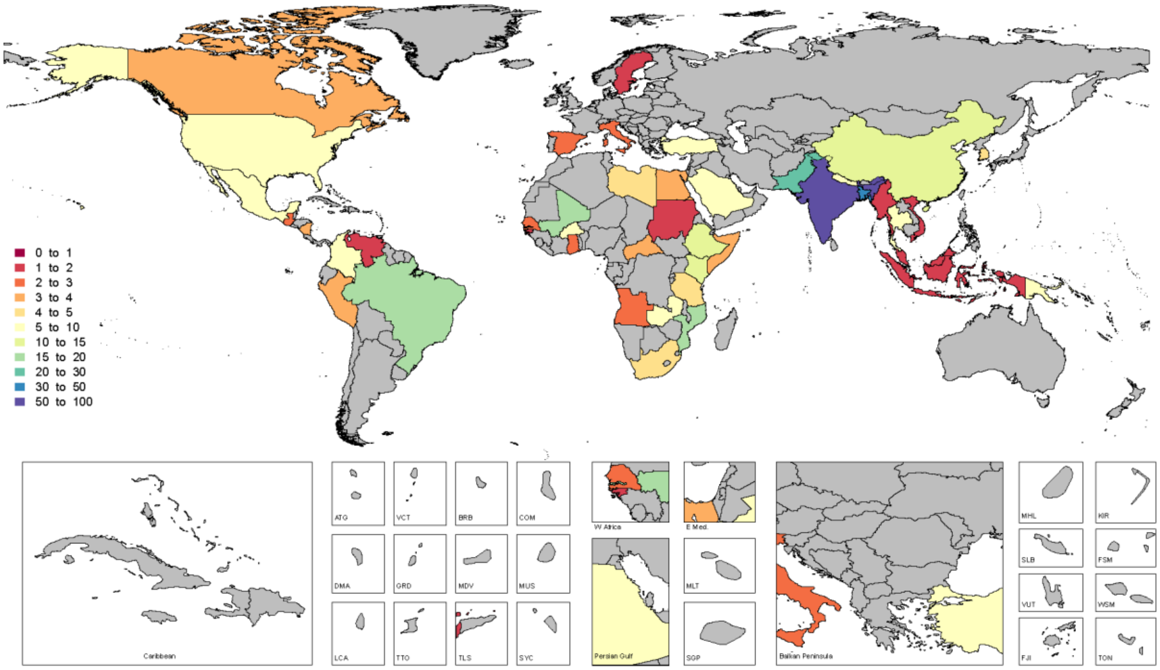

Number of observations for Campylobacter

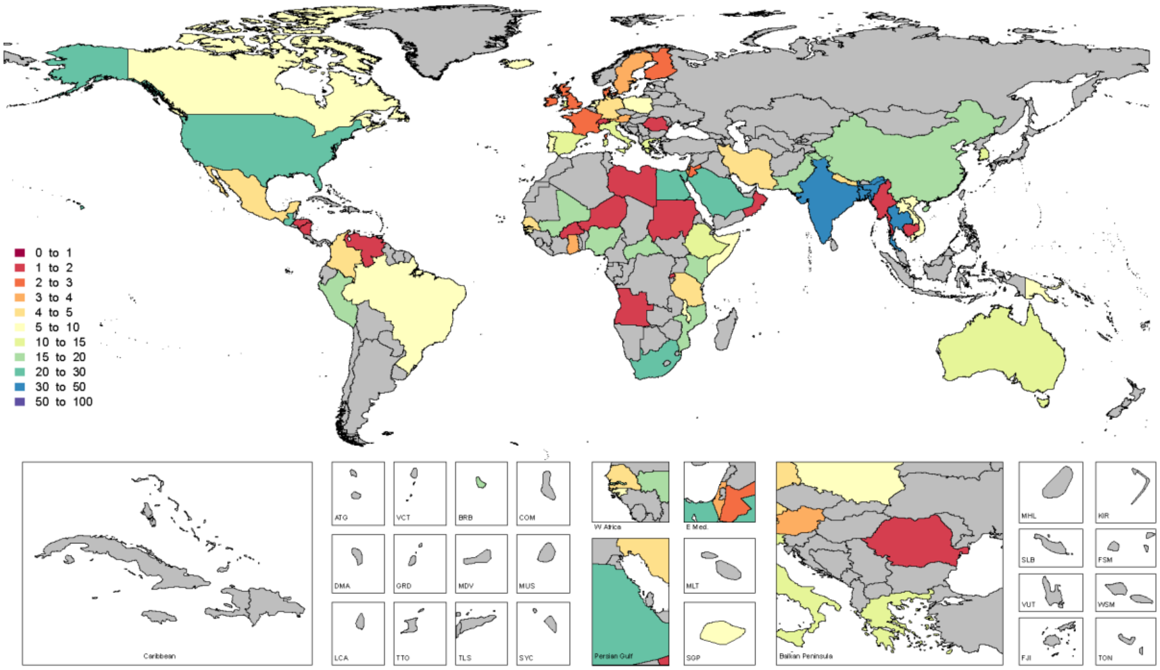

Number of observations for
Cholera

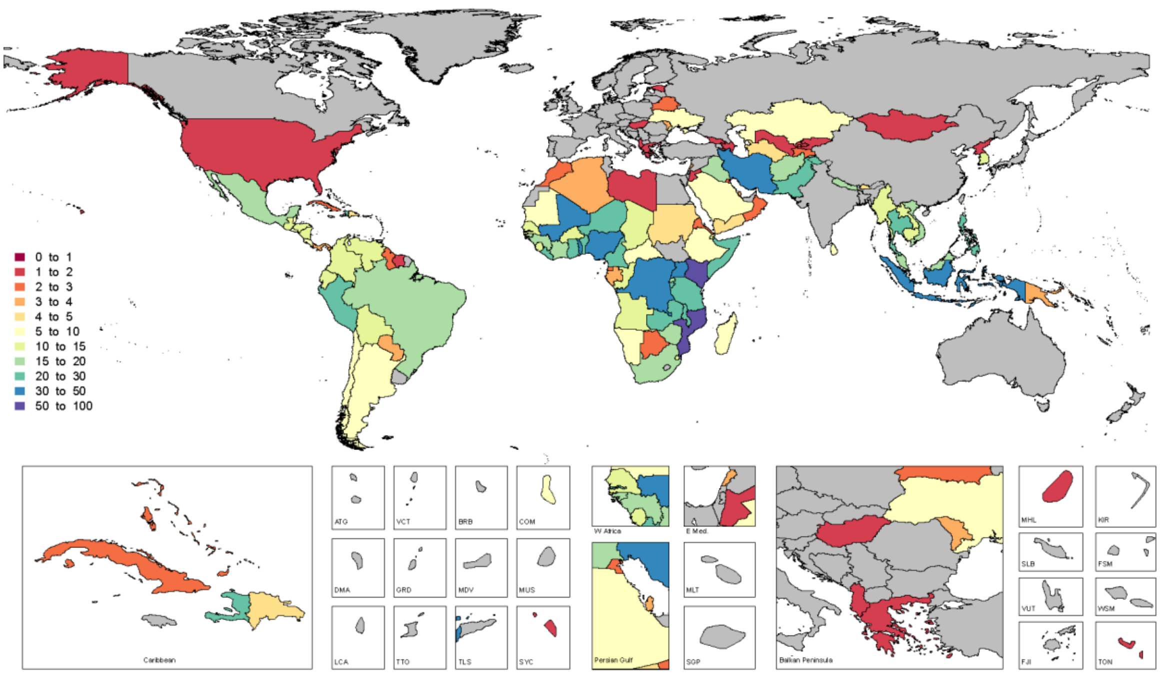

Number of observations for Clostridium difficile

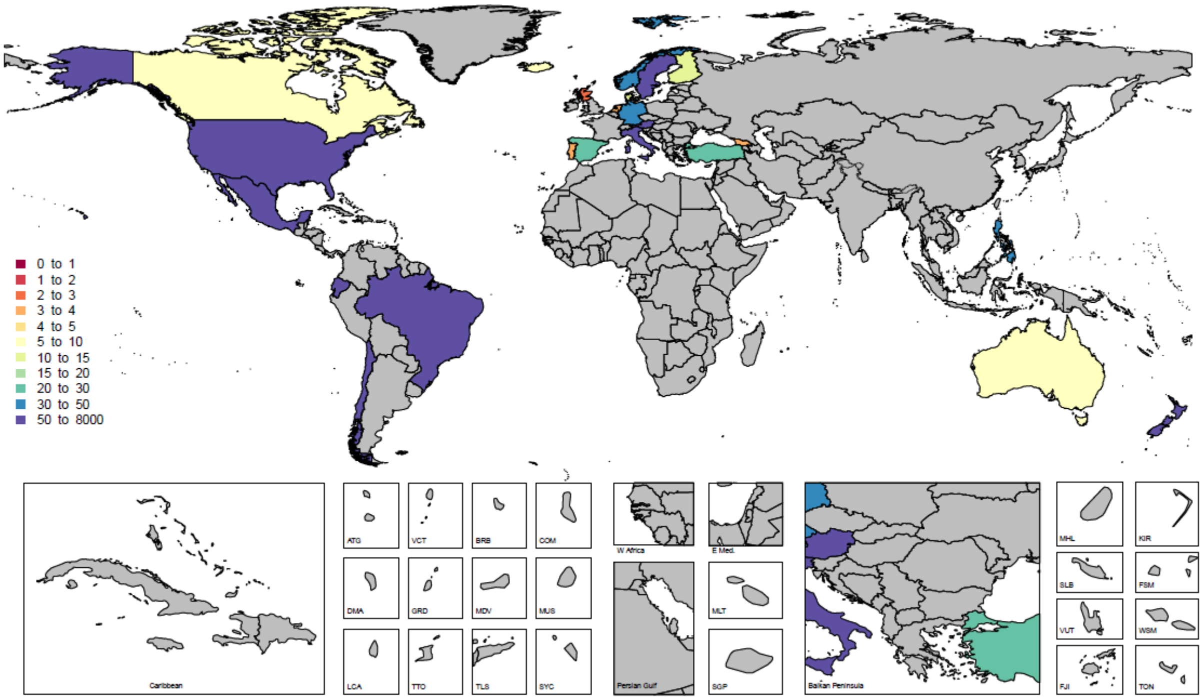

Number of observations for Cryptosporidium

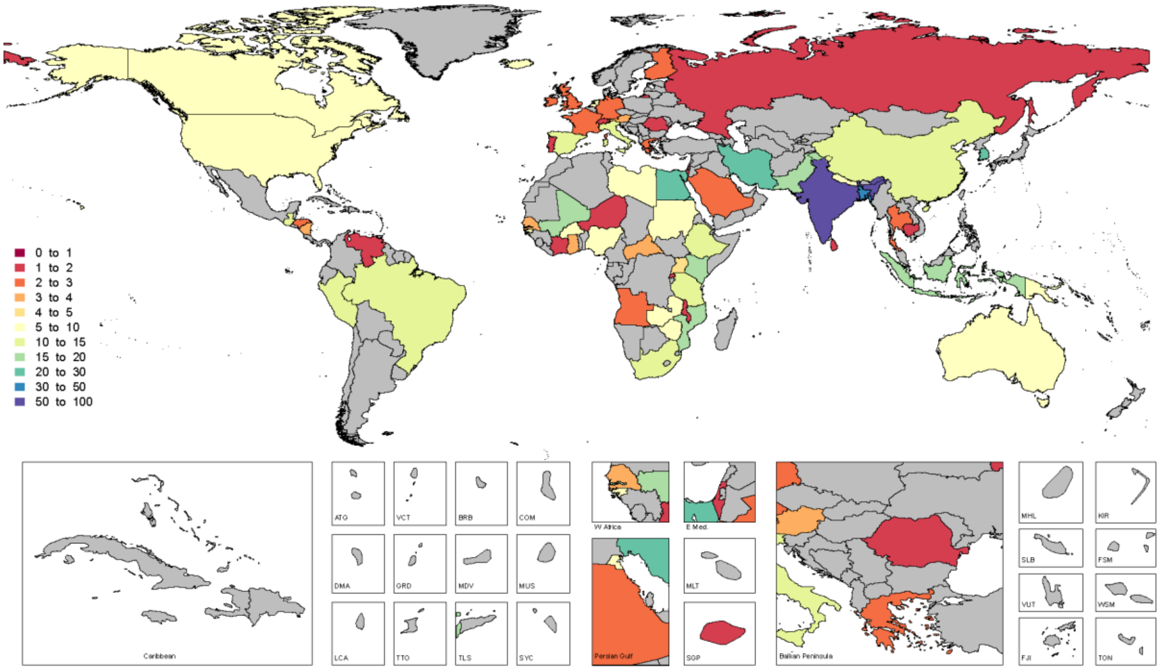

Number of observations for
EPEC

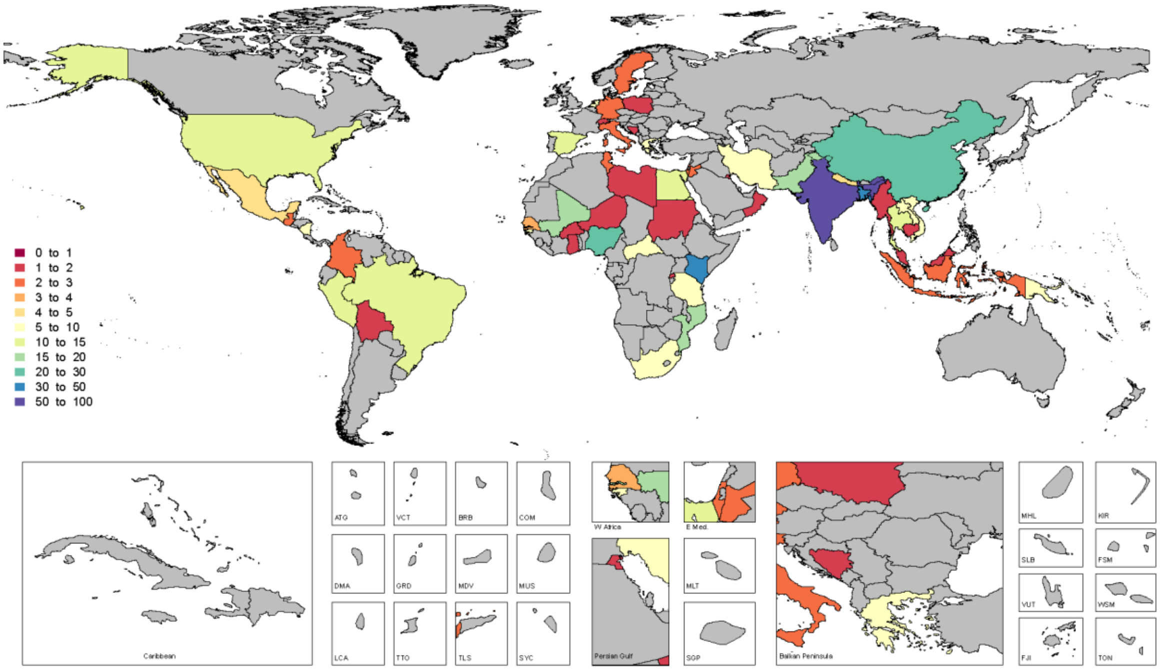

Number of observations for
ETEC

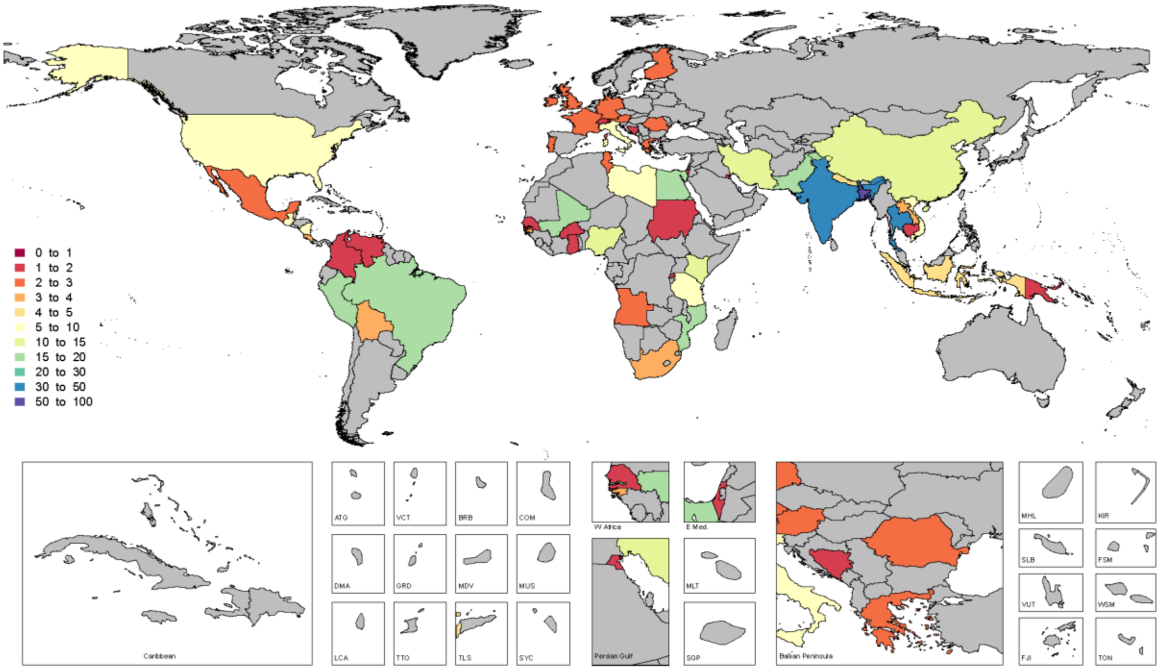

Number of observations for
Norovirus

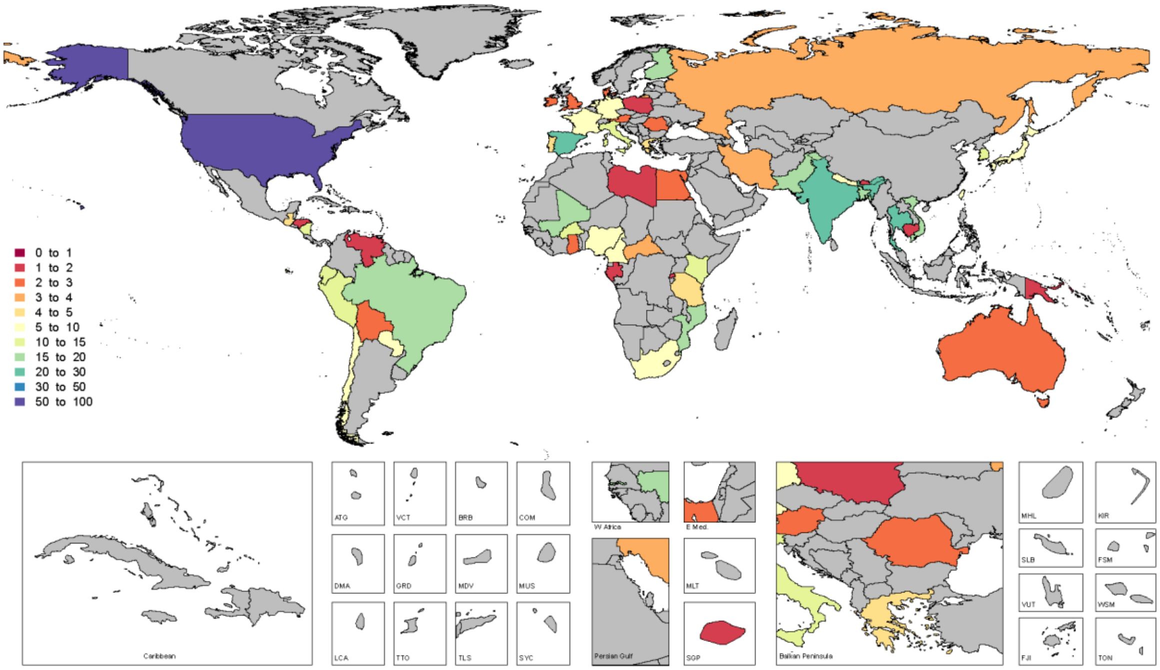

Number of observations for Rotavirus

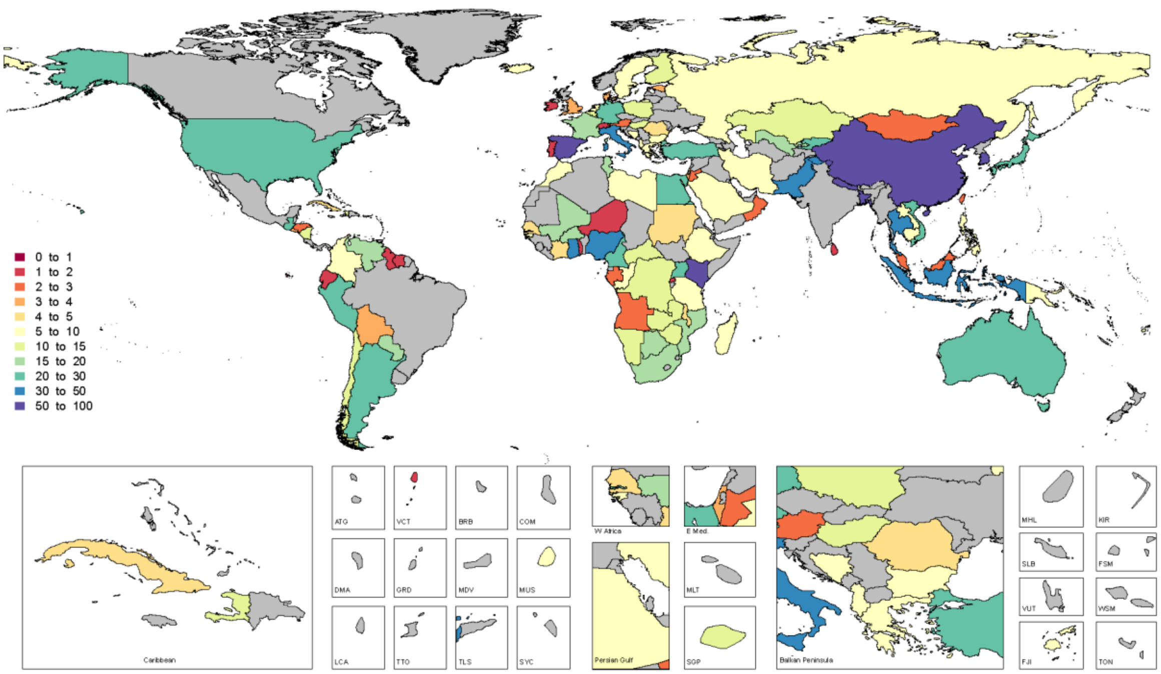

Number of observations for Salmonella

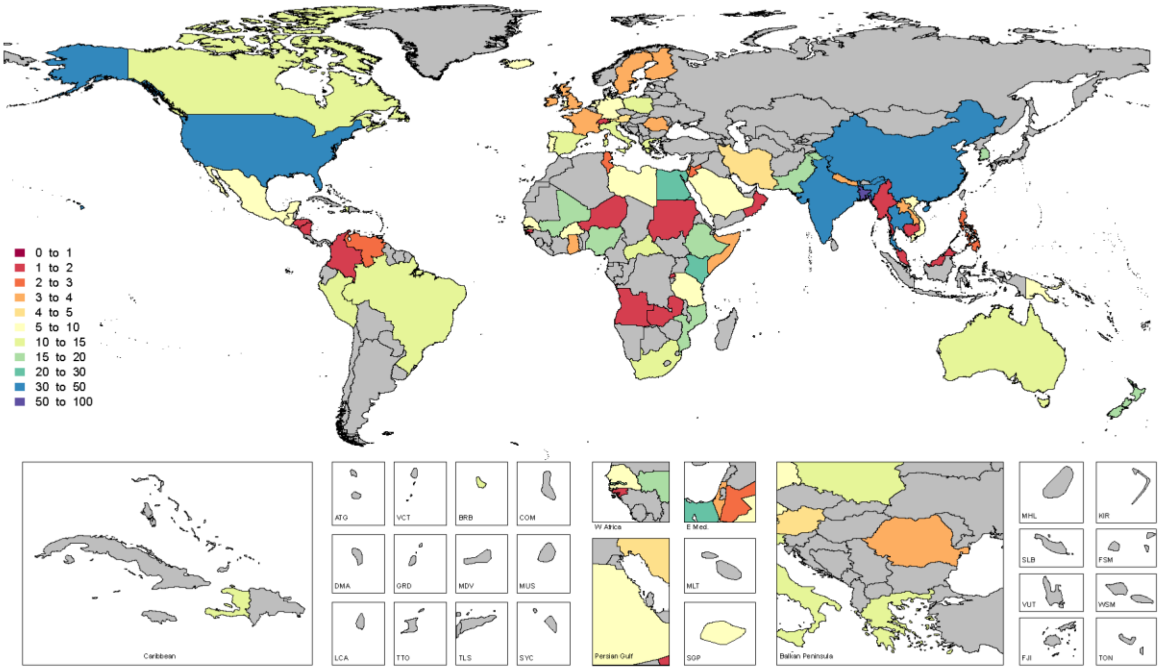

Number of observations for
Shigella

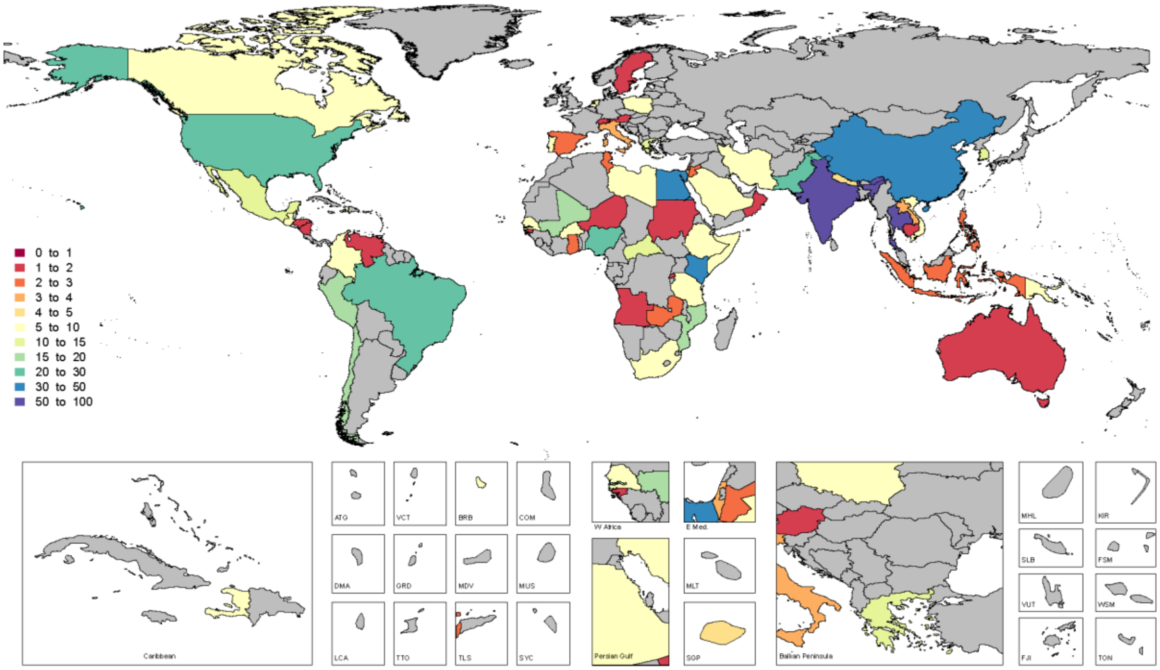

Summary of Risk Factors

Risk factors were estimated independently as part of the Global Burden of Disease study 2016 through a Comparative Risk Assessment framework. A core component of this process is determining a causal relationship between the risk and the outcome (diarrhoea). Each risk factor is composed of two parts. The first is an exposure and definition of a reference category with the lowest risk of diarrhoea. The second part is the association between the risk and diarrhoea, expressed as a relative risk, hazard ratio, or odds ratio. More information on each risk factor and the modelling strategy can be found in the Appendix to the GBD 2016 Risk Factors manuscript (http://www.thelancet.com/journals/lancet/article/PIIS0140-6736(17)32366-8/supplemental) on the pages given below:

- Childhood undernutrition (wasting, stunting, underweight)- the proportion of children under-5 that are below the global average weight, height, and weight-for-height: page 73
- Unsafe sanitation- defined based on the primary toilet type by household: page 46
- Unsafe water- defined based on the primary water source for a household before consumption: page 43
- Low access to handwashing- defined as lacking access to a handwashing station with available soap and water: page 49
- Suboptimal breastfeeding- the proportion of children under 2 years that are either breastfed non-exclusively or who receive no breast milk: page 71

We also utilized estimates of three interventions. These interventions were treated analogously to risk factors where we modeled the exposure to the intervention and the inverse of the relative risk of diarrhoea given access to the intervention.

- Low oral rehydration solution coverage- based on the proportion of children with diarrhoea in the previous two weeks that received ORS based on population representative surveys and the percent reduction in diarrhoea mortality given ORS based on a paper by Munos 2010.[21]
- Low rotavirus vaccine coverage- based on the proportion of children receiving the full three dose course of the rotavirus vaccine from surveys and scientific literature. The percent reduction in rotavirus mortality is from a systematic review by Lamberti et al.[22] The percent reduction in diarrhoea is the rotavirus PAF in a population multiplied by the low rotavirus vaccine coverage PAF.
- Low zinc treatment coverage- Therapeutic zinc coverage was defined based on the proportion of children with diarrhoea in the previous two weeks that received therapeutic zinc from population representative surveys and the percent reduction in diarrhoea mortality given zinc treatment from a paper by Walker and Black (2010).[23]

Appendix Figure 11. Clustered bar chart of the number of children under 5 needed to treat to prevent a diarrhoea death by super-region in 2016

The number needed to treat to prevent one death due to diarrhoea among children under 5 in 2016 is shown. The number neeeded to treat is the inverse of the risk-attributable diarrhoea mortality rate and is conceptually the difference between the observed and the counterfactual diarrhoea mortality rate in the absense of the risk factor.

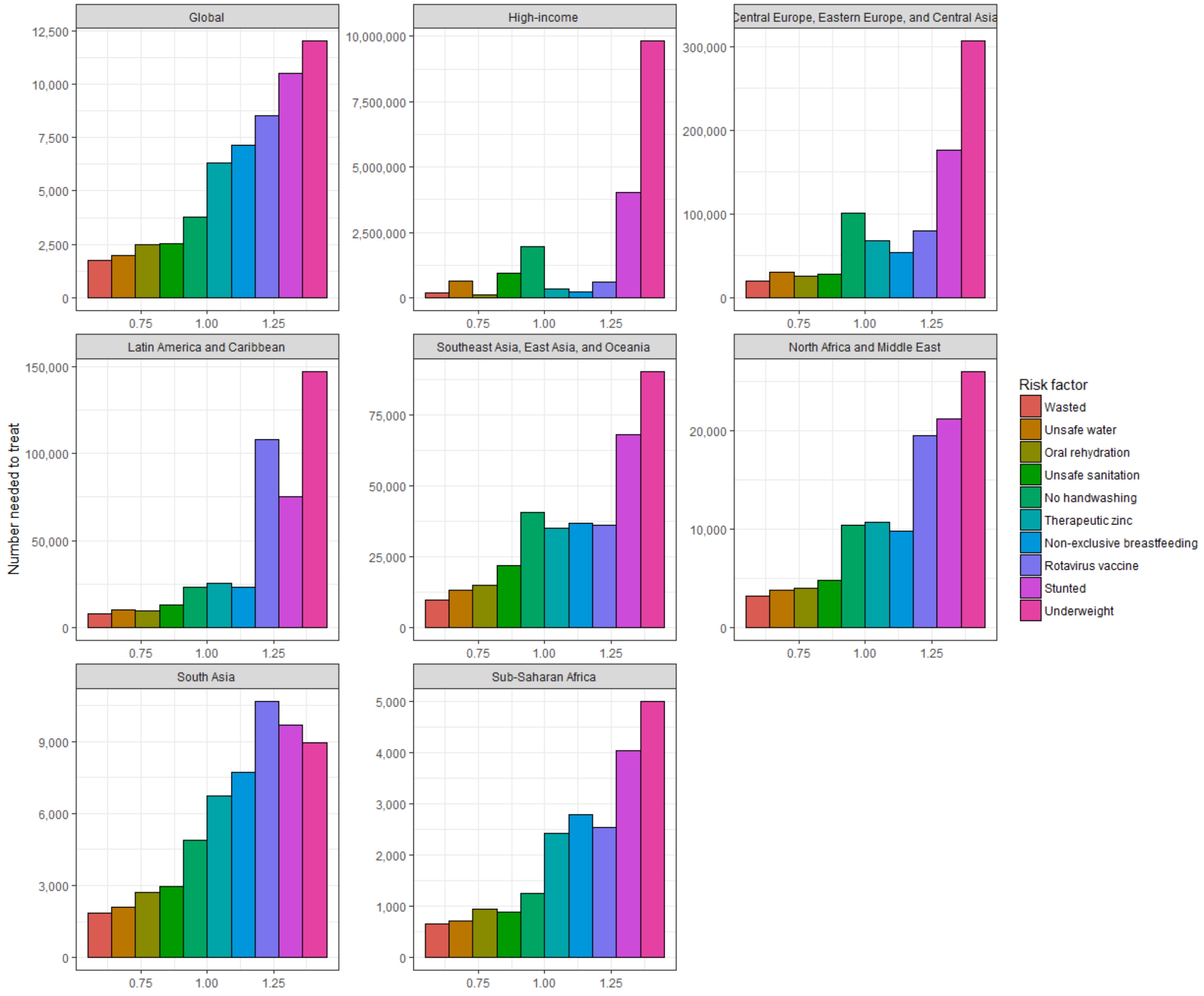

Comparison with GBD 2015

The differences in final estimates for diarrhoea mortality and morbidity between GBD 2015 and GBD 2016 are shown in **Appendix Figure 8.** The number of under-5 deaths in India in 2000 and 2015 deviates in GBD 2015 compared to GBD 2016, particularly among all ages, due mainly to changes in the most trusted data for India, the Sample Registration System from the Indian Ministry of Health. These updates in Cause of Death data are especially pronounced in locations with sparse data (**Appendix Figure 2**).

Appendix Figure 12. Scatterplot of GBD 2015 and GBD 2016 results by country in 2000 and 2015

A) The number of deaths among children under 5 years in 2000; **B)** The number of deaths among all ages in 2000; **C).** The number of deaths among children under 5 years in 2015; **D)** The number of deaths among all ages in 2015.

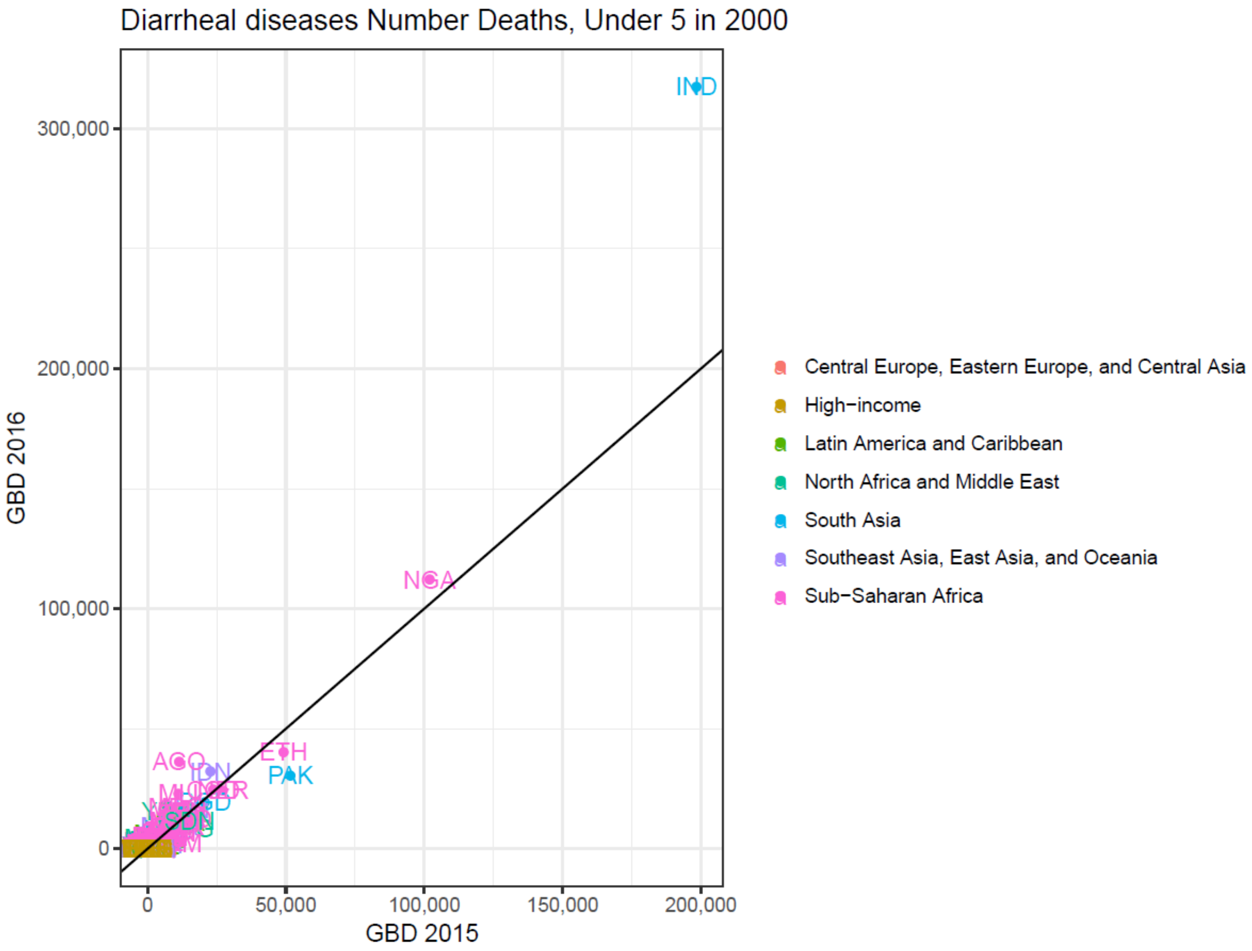

B)

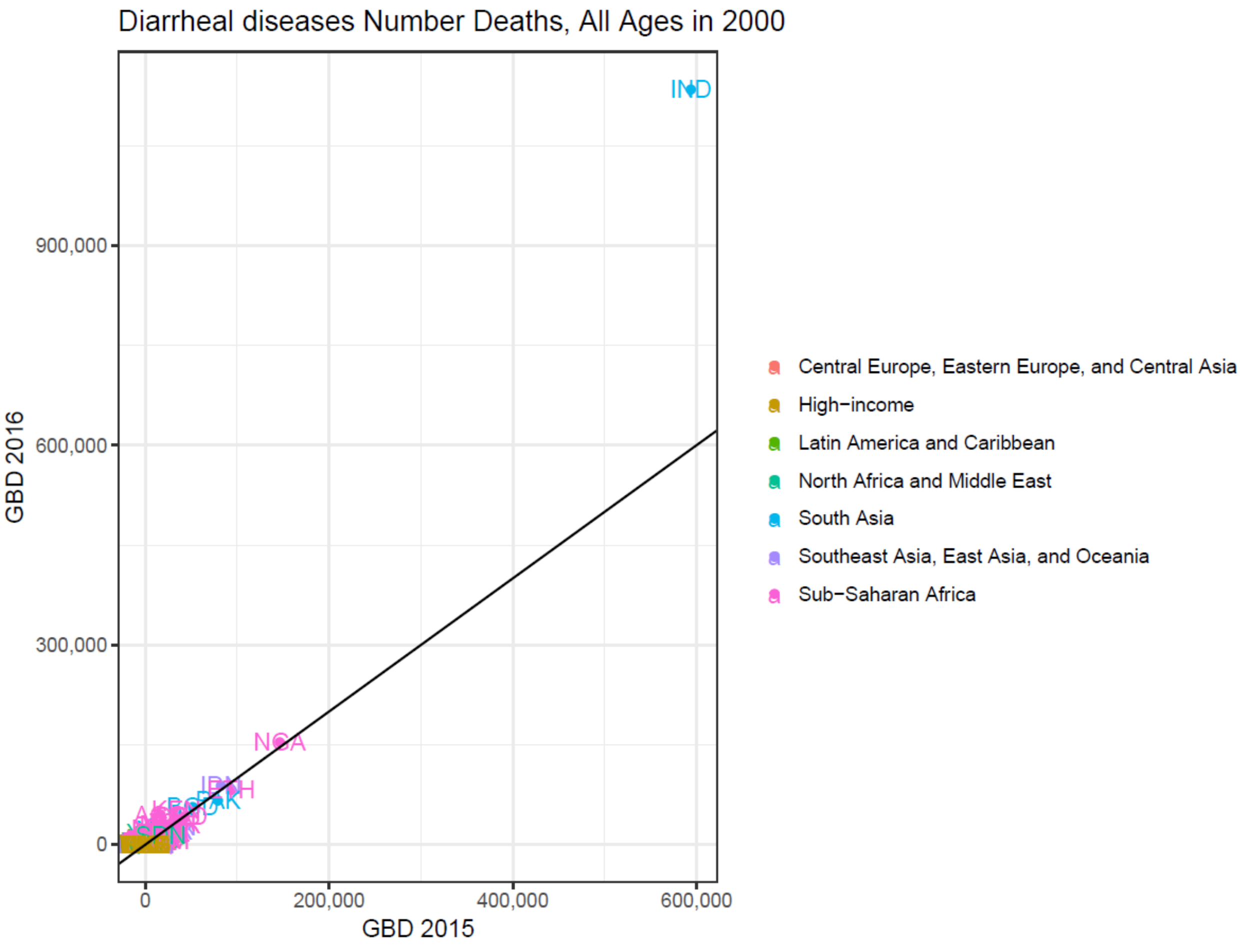
Diarrheal diseases Number Deaths, All Ages in 2000
GBD 2016
GBD 2015
900,000
600,000
300,000
0
200,000
400,000
600,000
IND
NGA
PAK
Central Europe, Eastern Europe, and Central Asia
High−income
Latin America and Caribbean
North Africa and Middle East
South Asia
Southeast Asia, East Asia, and Oceania
Sub−Saharan Africa

c)

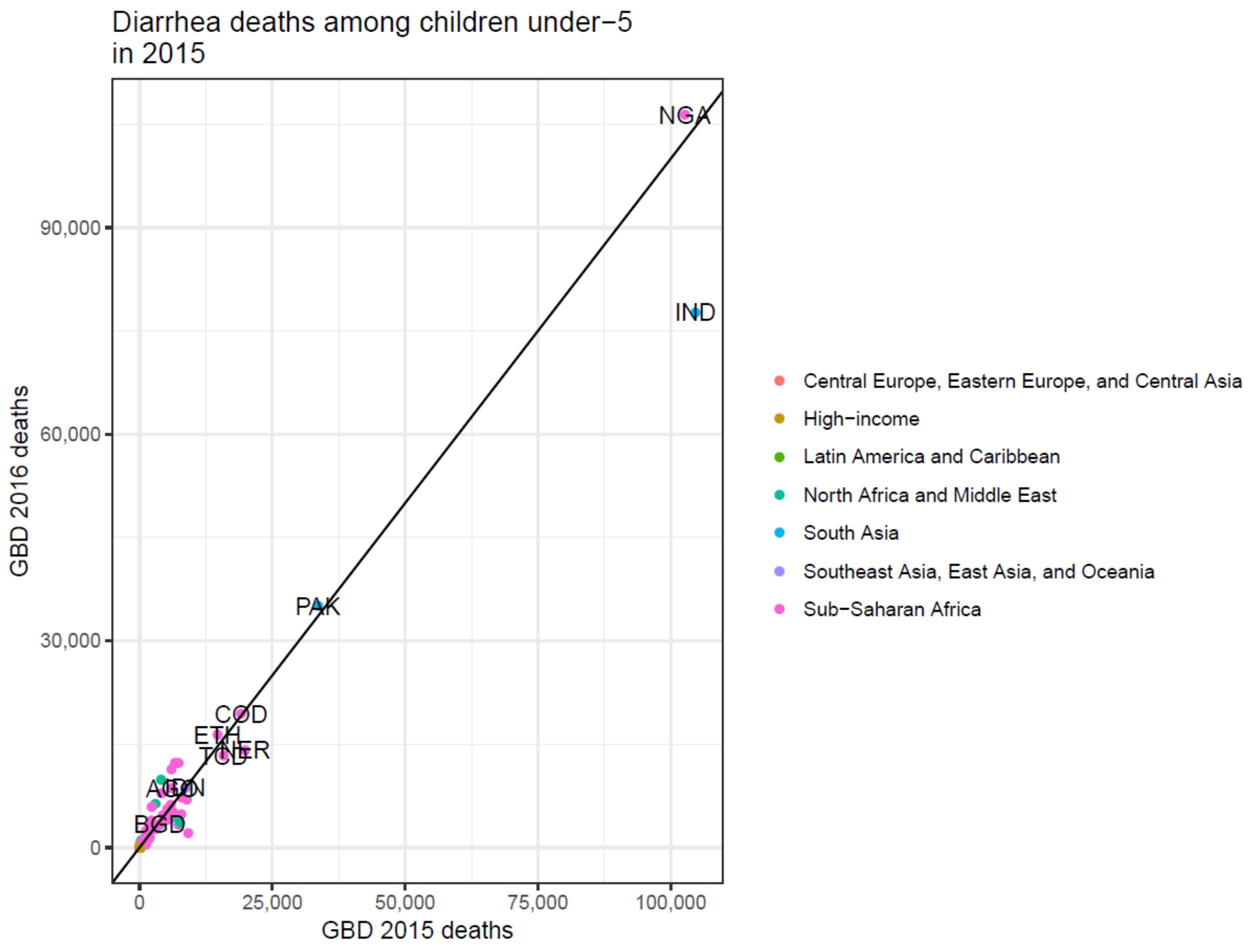
Diarrhea deaths among children under−5
in 2015
GBD 2016 deaths
GBD 2015 deaths
90,000
60,000
30,000
0
0
25,000
50,000
75,000
100,000
NGA
IND
PAK
COD
ETH
TCD
NER
AGO
CIV
BGD
Central Europe, Eastern Europe, and Central Asia
High−income
Latin America and Caribbean
North Africa and Middle East
South Asia
Southeast Asia, East Asia, and Oceania
Sub−Saharan Africa

D)

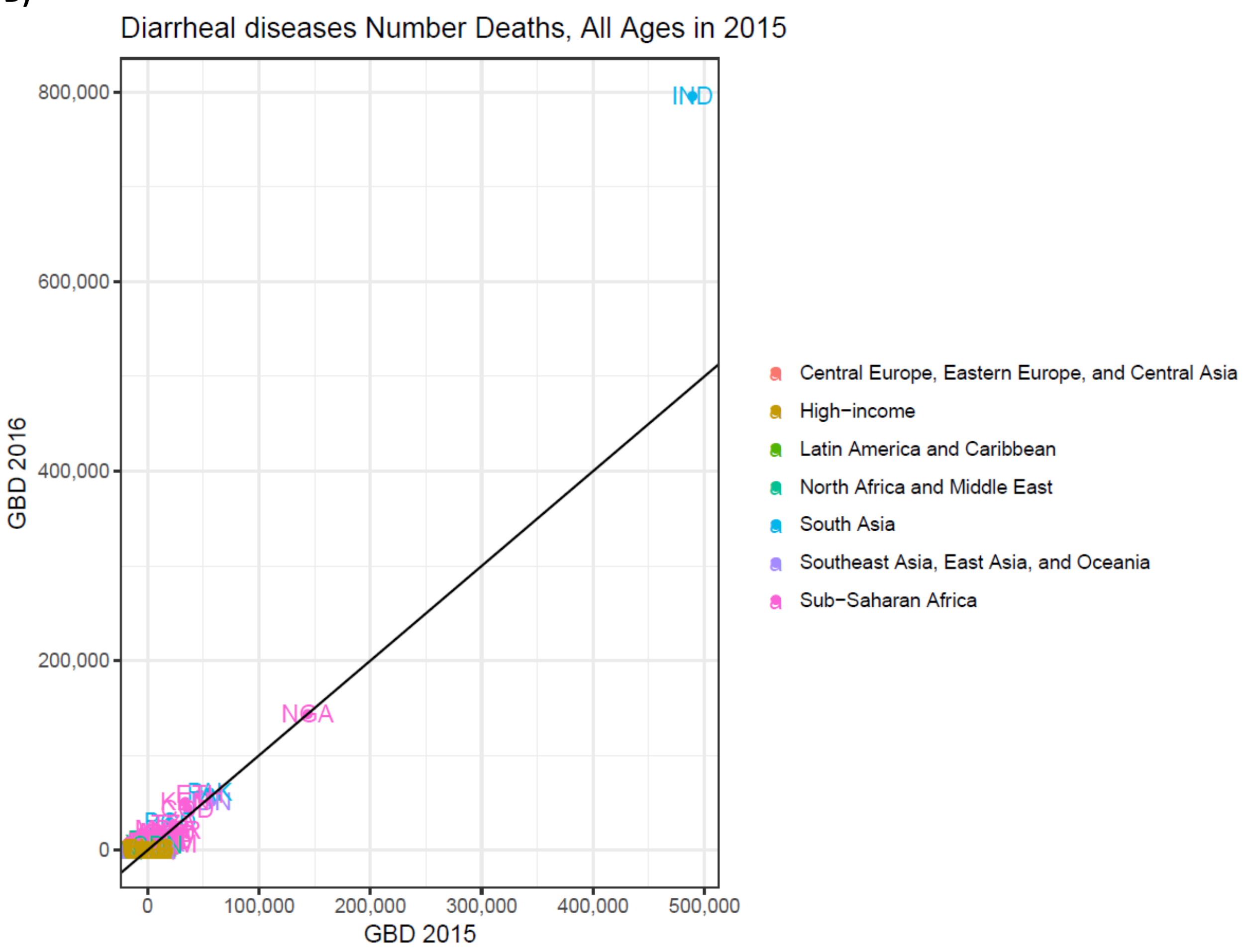
Diarrheal diseases Number Deaths, All Ages in 2015
800,000
600,000
400,000
200,000
0
GBD 2016
0
100,000
200,000
300,000
400,000
500,000
GBD 2015
IND
NGA
Central Europe, Eastern Europe, and Central Asia
High−income
Latin America and Caribbean
North Africa and Middle East
South Asia
Southeast Asia, East Asia, and Oceania
Sub−Saharan Africa

Appendix Figure 13. Under-5 mortality and all ages mortality between 1990 – 2016 between GBD 2015 and GBD 2016.

Further comparing the results from GBD 2015 and GBD 2016 for diarrhoea mortality shows that the estimates of under-5 mortality converged over time while the number of deaths for all ages remained systematically higher in GBD 2016.

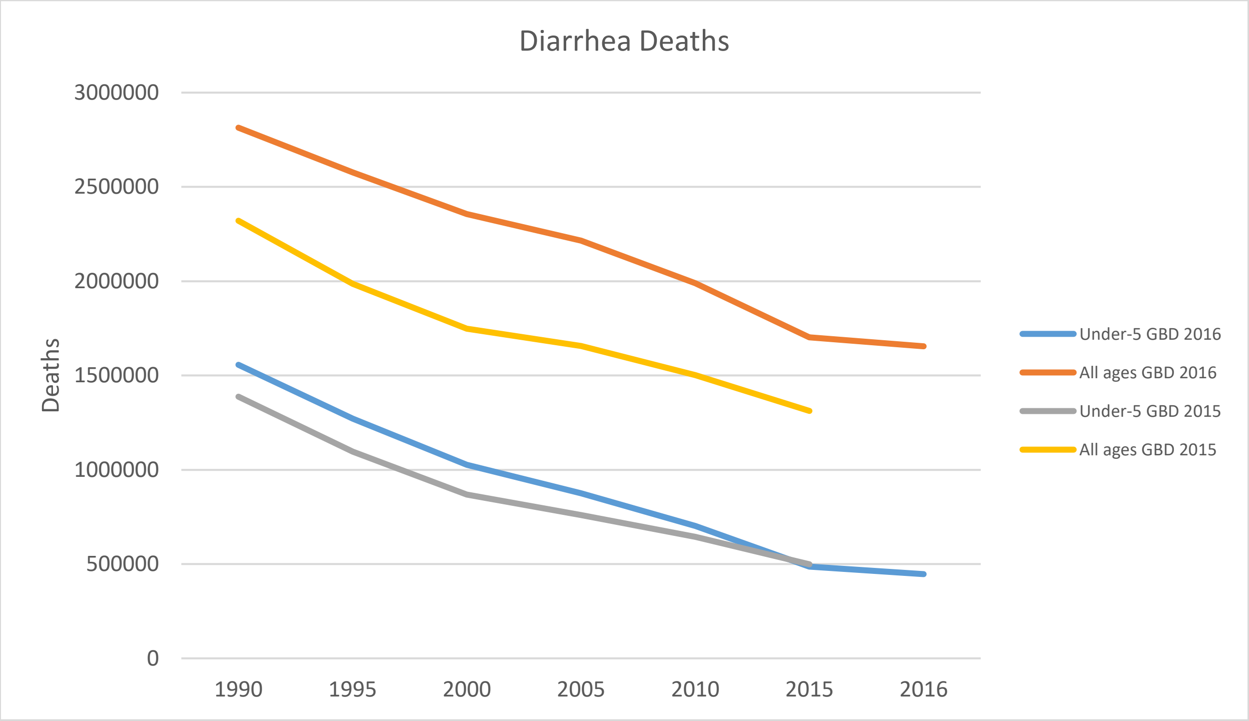

Overall, aetiologic attribution among children under-5 was very similar in 2010 in GBD 2015 and GBD 2016. GBD 2016 estimates appear to have improved the uncertainty around the mean values, due in part to fewer age groups used for the odds ratios in the aetiologic attribution. Further, there were additional data used in the modelling. Both GBD 2015 and GBD 2016 used molecular diagnostics as the case definition for pathogen detection which dramatically increased the attribution for nearly all pathogens.

Appendix Figure 14. Aetiologic attribution to diarrhoea deaths among children under 5 years old in 2010

The top pie plot shows the results at the global level for GBD 2013[24], GBD 2015, and GBD 2016

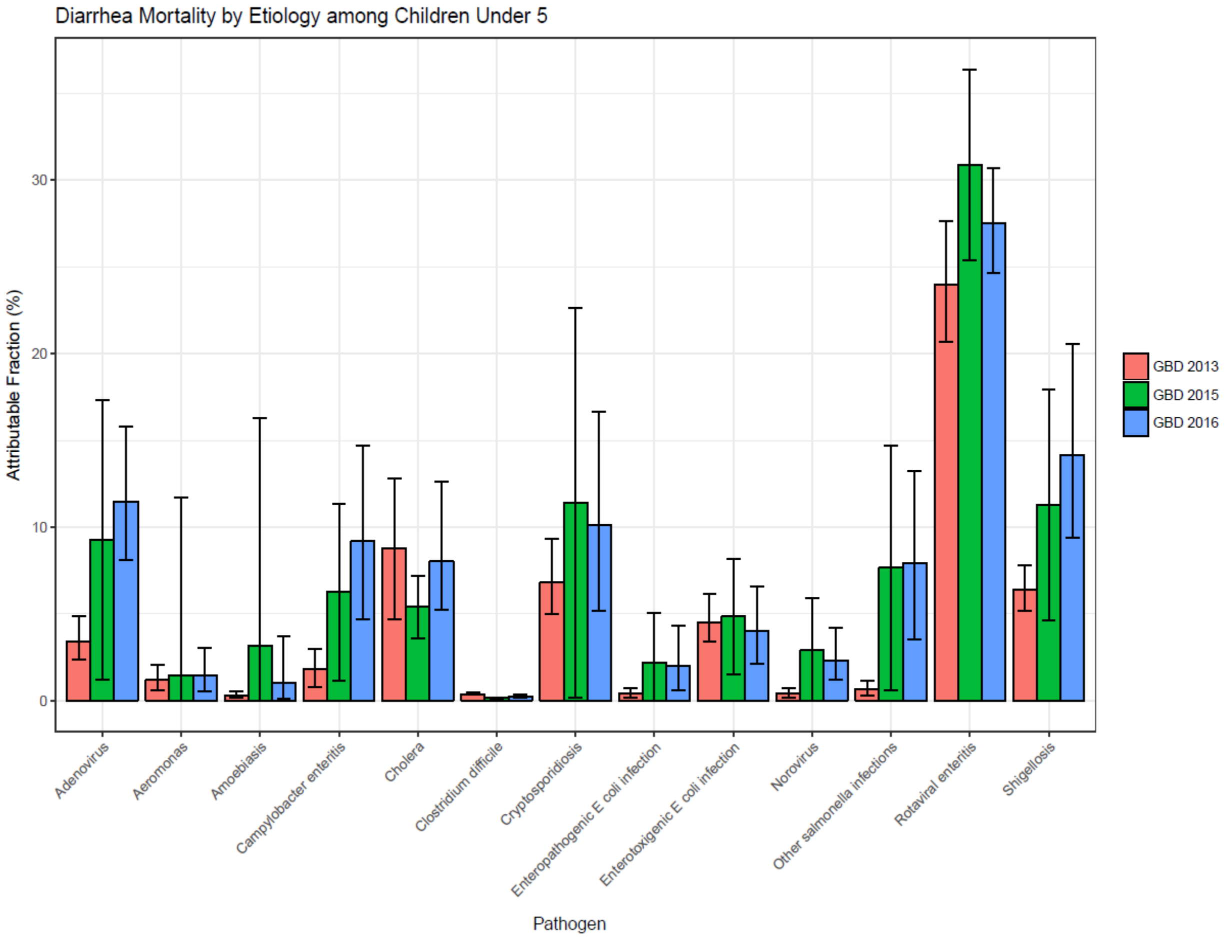

Comparison with WHO-MCEE

A comparison of the most recent estimates for aetiologic attribution to under-5 diarrhoea mortality between GBD 2016 and the CHERG-MCEE group is provided in the main text. The number of under-5 deaths by country in 2015 is shown in **Appendix Figure 14.**[25] Our estimates of diarrhoea mortality in children under 5 in 2015 (485,827, 95% UI 429,412-547,489) differ from those produced by the WHO Department of Evidence, Information and Research and the Maternal and Child Epidemiology Estimation (MCEE) group (526,000).[25] The main differences are in India and Nigeria. One possible reason for the large differences in the mortality estimates between the MCEE and GBD 2016 in India is the geographic precision in the modelling. Starting in GBD 2015, India has been estimated at the state and state-level urban/rural split. This has allowed for detailed estimates of subnational variation in diarrhoea mortality in the country. Further, the GBD 2016 relies nearly exclusively on the Sample Registration System for causes of death in India, a massive and comprehensive dataset that has data on the precision modelled in GBD 2016. As for Nigeria, the differences may arise from the input data used. GBD 2016 accepted a pair of verbal autopsy studies representative at the subnational level for Nigeria because of the GBD principle of incorporating as many data as possible in the analysis.

Appendix Table 7. Number of deaths due to diarrhoea among children under-5 in the year 2015 is shown for the WHO-MCEE group as well as for GBD 2015 and GBD 2016

	WHO-MCEE	GBD 2015	GBD 2016
**Global**	**525,977**	**482,592**	**485,800**
Indonesia	8,551	8,799	8,700
Bangladesh	7,189	3,177	3,375
India	117,285	110,736	77,684
Pakistan	39,484	39,017	35,030
DR Congo	32,047	19,204	19,414
Ethiopia	15,535	12,167	16,361
Nigeria	76,980	96,607	106,357

Appendix Figure 15. The number of under-5 deaths due to diarrhoea in 2015 by country

This scatterplot shows the number of deaths estimated by GBD 2016 and the WHO/MCEE group in 2015.

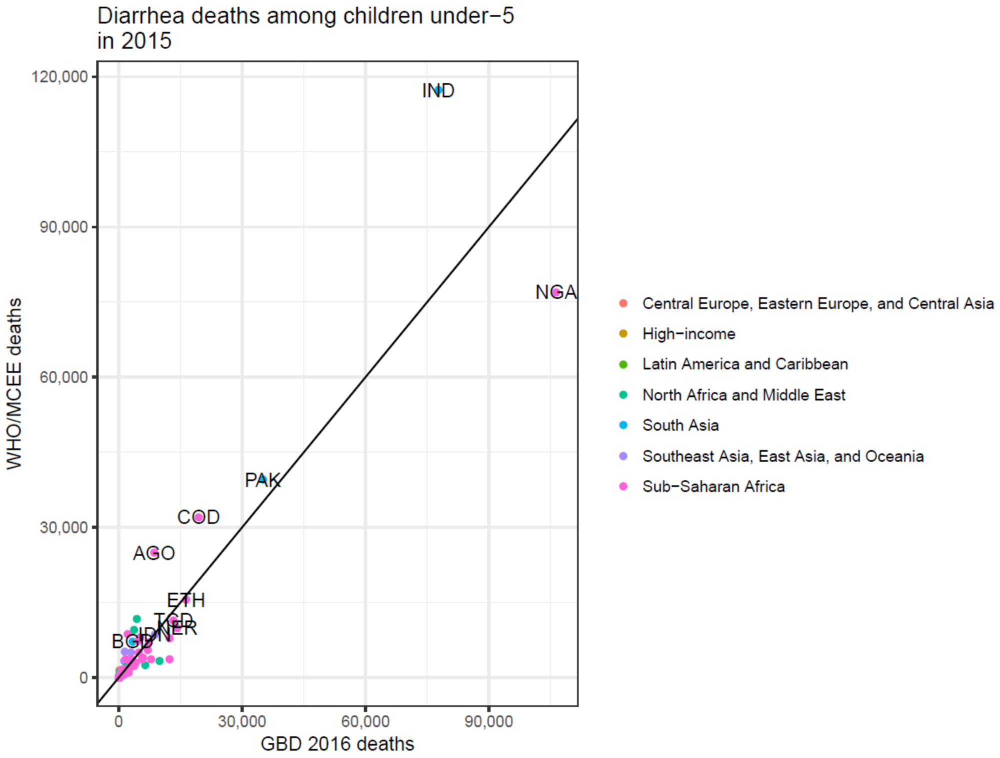

Among all ages, The WHO estimated that there were 1,388,418 deaths due to diarrhoea globally in 2015 which is lower than the GBD 2016 estimates for the same year (1,702,770 deaths, 95% UI 1,290,712-2,412,276).

The 2010 estimates for diarrhoea aetiologies for the CHERG-MCEE group and GBD 2016 are similar only for rotavirus (**Table 5**) while the other aetiologies vary by some large differences. Such differences may arise from varying methodological approaches, detailed elsewhere.[26] Two important differences are the application of molecular diagnostics in GBD 2016, which are not used by the CHERG-MCEE group, as well as the CHERG-MCEE group's categorical attribution of pathogen-specific diarrhoea proportion from hospitalised case data[27] whereas the GBD study uses a counterfactual approach to estimate population attributable fractions. We have shown that the use of molecular diagnostics as a case definition has a large impact on diarrhoea aetiologic attribution, increasing the detection rate for many bacterial pathogens by 20-60%.

Appendix Table 8. The number of diarrhoea deaths among children under-5 in 2010 by aetiology estimated by CHERG, GBD 2015, and GBD 2016

The year 2010 is used because it is the most recent common year for values across the three sets of estimates.

Aetiology	CHERG	GBD 2015	GBD 2016
Adenovirus	22 (12 to 37)	60 (22-126)	80.7 (55.3-114.7)
Aeromonas	-	9 (0-70)	10.3 (3.7-21.5)
Amoebiasis	1 (0 to 19)	20 (0-133)	6.9 (0.9-26.6)
Campylobacter	22 (11-50)	40 (11-78)	64.4 (32.3-101.8)
Cholera	9 (0 to 37)	34 (24-47)	56.3 (35.8-88.6)
Clostridium difficile	-	0.9 (0.8-1.0)	1.8 (1.4-2.3)
Cryptosporidiosis	14 (3-31)	76 (18-162)	71.0 (35.3-121.9)
Enteropathogenic E coli	79 (31-146)	14 (1-38)	13.9 (3.9-30.5)
Enterotoxigenic E coli	42 (20-76)	31 (14-57)	28.3 (15.0-46.0)
Norovirus	71 (39-113)	19 (8-45)	16.3 (8.2-30.3)
Salmonella	18 (10 to 30)	49 (16-108)	55.5 (24.3-94.5)
Rotavirus	197 (110 to 295)	199 (163-246)	193.3 (163.6-226.9)
Shigella	28 (12 to 53)	73 (36-127)	99.4 (64.8-144.7)

References

1 Naghavi M, Makela S, Foreman K, O'Brien J, Pourmalek F, Lozano R. Algorithms for enhancing public health utility of national causes-of-death data. *Popul Health Metr* 2010; **8**: 9.

2 GBD 2015 Mortality and Causes of Death Collaborators. Global, regional, and national life expectancy, all-cause and cause-specific mortality for 249 causes of death, 1980–2015: a systematic analysis for the Global Burden of Disease Study 2015. *Lancet* 2016; **388**: 1459–544.

3 Foreman KJ, Lozano R, Lopez AD, Murray CJ. Modeling causes of death: an integrated approach using CODEm. *Popul Health Metr* 2012; **10**: 1.

4 Foreman KJ, Lozano R, Lopez AD, Murray CJ. Modeling causes of death: an integrated approach using CODEm. *Popul Health Metr* 2012; **10**: 1.

5 GBD 2016 Mortality Collaborators. Global, regional, and national under-5 mortality, adult mortality, age-specific mortality, and life expectancy, 1970-2016: a systematic analysis for the Global Burden of Disease Study 2016. *Lancet Lond Engl* 2017; **390**: 1084–150.

6 GBD 2016 Risk Factors Collaborators. Global, regional, and national comparative risk assessment of 84 behavioural, environmental and occupational, and metabolic risks or clusters of risks, 1990-2016: a systematic analysis for the Global Burden of Disease Study 2016. *Lancet Lond Engl* 2017; **390**: 1345–422.

7 GBD 2015 Risk Factors Collaborators. Global, regional and national comparative risk assessment of 79 behavioural, environmental and occupational, and metabolic risks or clusters of risks in 195 countries, 1990-2015: a systematic analysis for the Global Burden of Disease Study 2015. *Lancet* 2016; **388**: 1659–724.

8 GBD 2016 Disease and Injury Incidence and Prevalence Collaborators. Global, regional, and national incidence, prevalence, and years lived with disability for 328 diseases and injuries for 195 countries, 1990-2016: a systematic analysis for the Global Burden of Disease Study 2016. *Lancet Lond Engl* 2017; **390**: 1211–59.

9 GBD 2015 Healthcare Access and Quality Collaborators. Electronic address: cjlm@uw.edu, GBD 2015 Healthcare Access and Quality Collaborators. Healthcare Access and Quality Index based on mortality from causes amenable to personal health care in 195 countries and territories, 1990-2015: a novel analysis from the Global Burden of Disease Study 2015. *Lancet Lond Engl* 2017; **390**: 231–66.

10 Lamberti LM, Fischer Walker CL, Black RE. Systematic review of diarrhea duration and severity in children and adults in low- and middle-income countries. *BMC Public Health* 2012; **12**: 276.

11 World Health Organization: Department of Child and Adolescent Health and Development. Handbook Integrated Management of Childhood Illness. 2005.

12 Salomon JA, Haagsma JA, Davis A, *et al.* Disability weights for the Global Burden of Disease 2013 study. *Lancet Glob Health* 2015; **3**: e712-723.

13 Miettinen OS. Proportion of disease caused or prevented by a given exposure, trait or intervention. *Am J Epidemiol* 1974; **99**: 325–32.

14 Liu J, Kabir F, Manneh J, *et al.* Development and assessment of molecular diagnostic tests for 15 enteropathogens causing childhood diarrhoea: a multicentre study. *Lancet Infect Dis* 2014; **14**: 716–24.

15 Liu J, Gratz J, Amour C, *et al.* A laboratory-developed TaqMan Array Card for simultaneous detection of 19 enteropathogens. *J Clin Microbiol* 2013; **51**: 472–80.

16 Reiczigel J, Földi J, Ozsvári L. Exact confidence limits for prevalence of a disease with an imperfect diagnostic test. *Epidemiol Infect* 2010; **138**: 1674–8.

17 Rothman K, Greenland S, Lash T. Modern Epidemiology, Third Edition. Philadelphia: Lippincott Williams & Wilkins, 2008.

18 Platts-Mills JA, Operario DJ, Houpt ER. Molecular diagnosis of diarrhea: current status and future potential. *Curr Infect Dis Rep* 2012; **14**: 41–6.

19 Ochoa TJ, Barletta F, Contreras C, Mercado E. New insights into the epidemiology of enteropathogenic Escherichia coli infection. *Trans R Soc Trop Med Hyg* 2008; **102**: 852–6.

20 World Health Organization. Global Health Observatory data repository: Cholera. 2016. http://apps.who.int/gho/data/node.main.174?lang=en (accessed Aug 25, 2016).

21 Munos MK, Walker CLF, Black RE. The effect of oral rehydration solution and recommended home fluids on diarrhoea mortality. *Int J Epidemiol* 2010; **39**: i75–87.

22 Lamberti LM, Ashraf S, Walker CLF, Black RE. A Systematic Review of the Effect of Rotavirus Vaccination on Diarrhea Outcomes Among Children Younger Than 5 Years. *Pediatr Infect Dis J* 2016; **35**: 992–8.

23 Walker CLF, Black RE. Zinc for the treatment of diarrhoea: effect on diarrhoea morbidity, mortality and incidence of future episodes. *Int J Epidemiol* 2010; **39**: i63–9.

24 GBD 2013 Mortality and Causes of Death Collaborators. Global, regional, and national age-sex specific all-cause and cause-specific mortality for 240 causes of death, 1990-2013: a systematic analysis for the Global Burden of Disease Study 2013. *Lancet Lond Engl* 2015; **385**: 117–71.

25 WHO. Estimates for 2000-2015. http://www.who.int/healthinfo/global_burden_disease/estimates_child_cod_2015/en/ (accessed Aug 25, 2016).

26 Kovacs SD, Mullholland K, Bosch J, *et al.* Deconstructing the differences: a comparison of GBD 2010 and CHERG's approach to estimating the mortality burden of diarrhea, pneumonia, and their etiologies. *BMC Infect Dis* 2015; **15**: 16.

27 Lanata CF, Fischer-Walker CL, Olascoaga AC, *et al.* Global causes of diarrheal disease mortality in children <5 years of age: a systematic review. *PloS One* 2013; **8**: e72788.

Supplementary Results

This section contains supplementary results for the manuscript. All models and results can be found using several online resources for a curious reader.

- **GBD Compare:** https://vizhub.healthdata.org/gbd-compare/
    - This resource provides the most current estimates for nearly all results available from the Global Burden of Disease Study and allows the user to take screenshots, download the data available in the webpage, and gives a comprehensive picture of health loss globally.

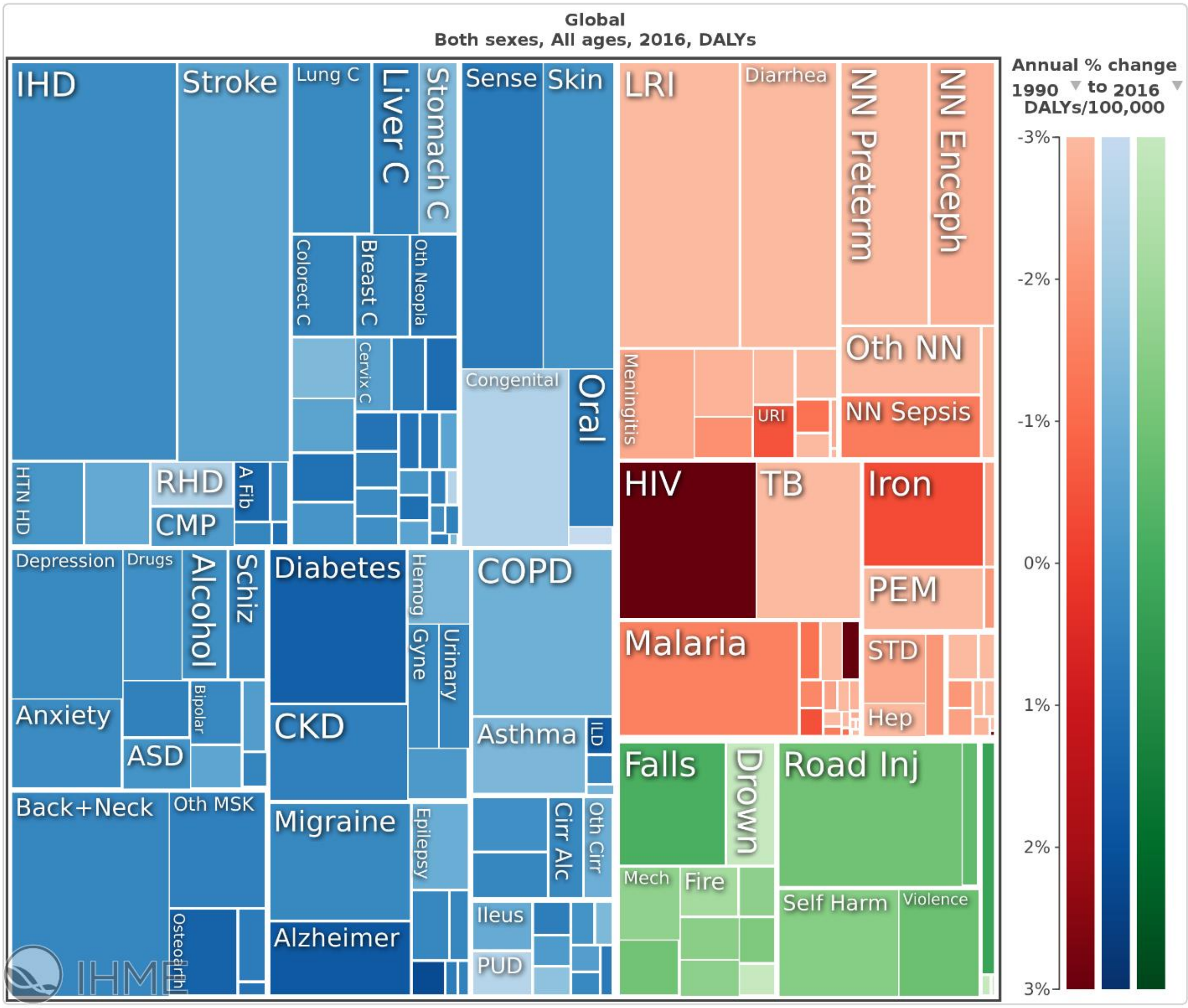

- **Global Health Data Exchange:** https://ghdx.healthdata.org/
    - This resource provides a comprehensive library of health data used in the Global Burden of Disease study and is intended to be a resource available for the entire global health community to identify data and sources.

- **Cause of Death modeling:** https://vizhub.healthdata.org/cod/

- o This tool shows the intermediate models for causes of death in the global burden of disease study, including for diarrhoeal diseases (**[http://ihmeuw.org/4dq0)](http://ihmeuw.org/4dq0)**. The tool shows the final model fits used in the GBD 2016 including input data for every location and age group.

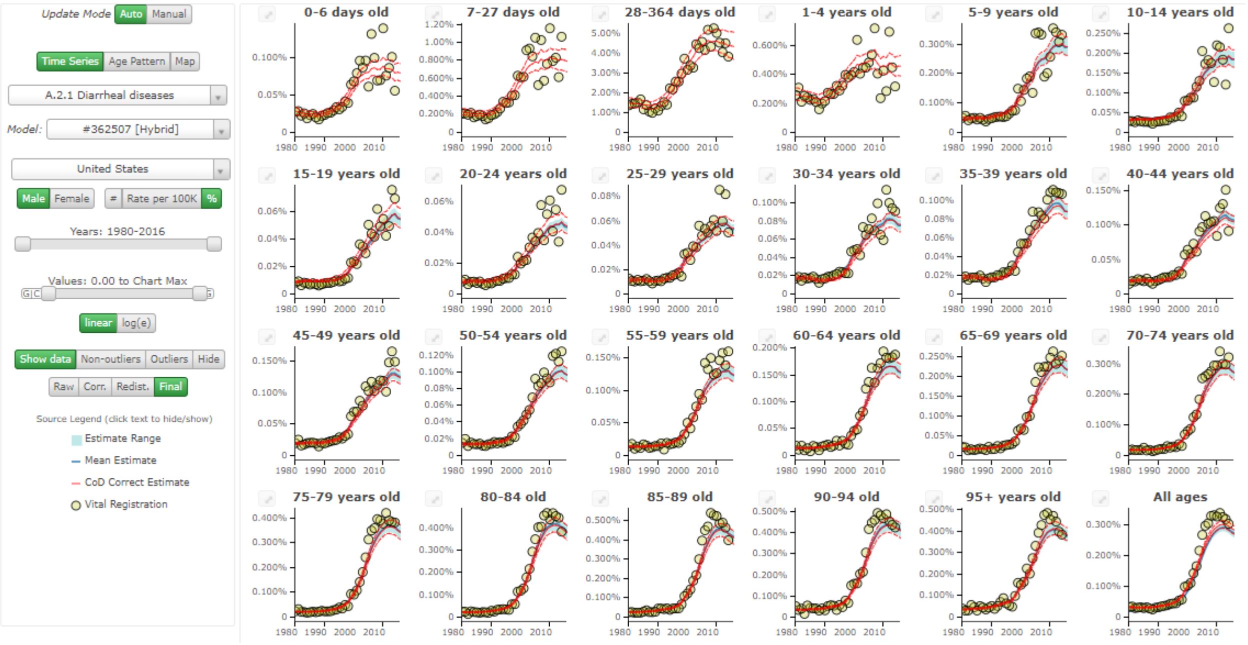

- **DisMod-MR 2.1:** **https://vizhub.healthdata.org/epi/**

- This tool shows the model results for the non-fatal models used in the Global Burden of Disease study 2016, including for diarrhoeal diseases ([http://ihmeuw.org/4ac5)](http://ihmeuw.org/4ac5). The tool shows the model fits and input data.

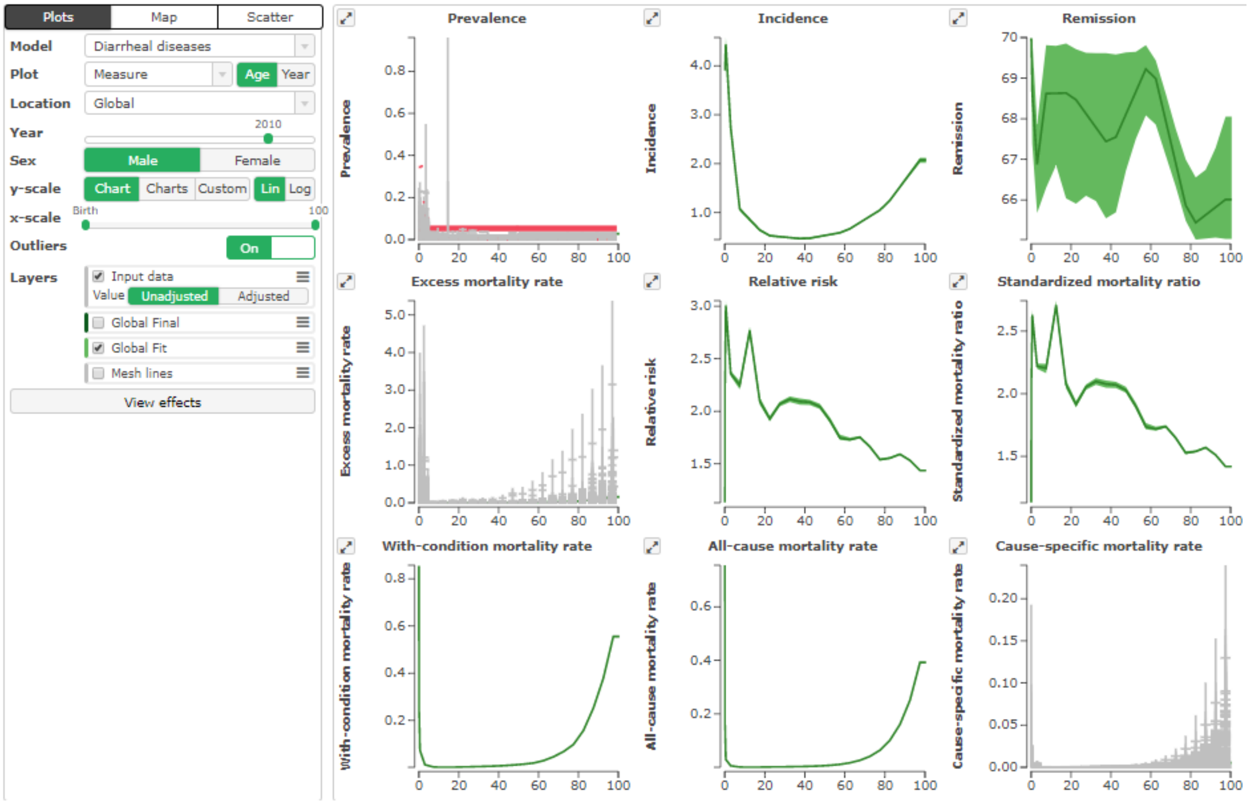

Appendix Table 9. The number of deaths, mortality rate per 100,000, episodes in millions, and incidence per 1000 person-years in 2016 by GBD region and country among all ages, children under-5 and adults over-70 years.

	All ages				Children under 5				Adults over 70			
Location	Deaths (95% UI)	Deaths per 100,000 (95% UI)	Millions of episodes (95% UI)	Incidence per person (95% UI)	Deaths (95% UI)	Deaths per 100,000 (95% UI)	Millions of episodes (95% UI)	Incidence per person (95% UI)	Deaths (95% UI)	Deaths per 100,000 (95% UI)	Millions of episodes (95% UI)	Incidence per person (95% UI)
**Global**	**1,655,944 (1,244,073-2,366,552)**	**22.4 (16.8-32.0)**	**4,480.40 (4,247.00-4,737.77)**	**0.61 (0.57-0.64)**	**446,000 (390,894-504,613)**	**70.6 (61.9-79.8)**	**1,105.41 (961.60-1,274.77)**	**1.75 (1.52-2.02)**	**694,010 (461,118-1,065,409)**	**171.7 (114.1-263.5)**	**364.93 (331.94-403.16)**	**0.90 (0.82-1.00)**
**High-income**	**31,267 (29,970-32,742)**	**2.9 (2.8-3.1)**	**141.63 (133.51-150.14)**	**0.13 (0.13-0.14)**	**761 (693-839)**	**1.3 (1.2-1.5)**	**33.39 (27.83-40.61)**	**0.58 (0.48-0.70)**	**26,909 (25,688-28,313)**	**20.1 (19.2-21.2)**	**22.60 (21.03-24.28)**	**0.17 (0.16-0.18)**
**High-income North America**	**10,919 (10,506-11,345)**	**3.0 (2.9-3.2)**	**48.92 (46.53-51.53)**	**0.14 (0.13-0.14)**	**394 (352-440)**	**1.8 (1.6-2.0)**	**9.06 (7.76-10.67)**	**0.42 (0.36-0.49)**	**8,740 (8,359-9,111)**	**24.5 (23.4-25.5)**	**4.02 (3.75-4.32)**	**0.11 (0.10-0.12)**
Canada	1,514 (1,337-1,698)	4.2 (3.7-4.7)	3.18 (2.98-3.40)	0.09 (0.08-0.09)	13 (10-18)	0.7 (0.5-1.0)	0.55 (0.45-0.67)	0.28 (0.23-0.34)	1,344 (1,174-1,528)	35.3 (30.8-40.1)	0.44 (0.41-0.48)	0.12 (0.11-0.13)
Greenland	1 (0-2)	1.6 (0.6-3.3)	0.01 (0.01-0.01)	0.11 (0.10-0.12)	0 (0-0)	2.4 (1.2-4.4)	0.00 (0.00-0.00)	0.37 (0.30-0.46)	0 (0-1)	19.1 (5.4-47.9)	0.00 (0.00-0.00)	0.10 (0.09-0.11)
United States	9,404 (9,063-9,751)	2.9 (2.8-3.0)	45.72 (43.50-48.09)	0.14 (0.13-0.15)	380 (340-426)	1.9 (1.7-2.2)	8.50 (7.30-10.00)	0.43 (0.37-0.51)	7,396 (7,086-7,706)	23.2 (22.2-24.2)	3.58 (3.33-3.84)	0.11 (0.10-0.12)
**Australasia**	**322 (291-355)**	**1.1 (1.0-1.2)**	**2.17 (2.03-2.31)**	**0.08 (0.07-0.08)**	**12 (9-15)**	**0.7 (0.5-0.9)**	**0.23 (0.19-0.29)**	**0.13 (0.11-0.16)**	**276 (245-308)**	**9.7 (8.6-10.8)**	**0.23 (0.21-0.25)**	**0.08 (0.07-0.09)**
Australia	257 (228-286)	1.1 (0.9-1.2)	1.59 (1.49-1.71)	0.07 (0.06-0.07)	9 (7-12)	0.6 (0.5-0.8)	0.17 (0.13-0.21)	0.11 (0.09-0.14)	219 (191-248)	9.1 (7.9-10.3)	0.17 (0.16-0.19)	0.07 (0.06-0.08)
New Zealand	65 (57-75)	1.4 (1.3-1.7)	0.57 (0.54-0.61)	0.13 (0.12-0.13)	3 (2-3)	0.9 (0.6-1.2)	0.07 (0.05-0.08)	0.23 (0.19-0.29)	58 (50-67)	12.7 (10.9-14.8)	0.06 (0.06-0.07)	0.13 (0.12-0.14)
**High-income Asia Pacific**	**4,126 (3,567-5,137)**	**2.3 (2.0-2.8)**	**9.86 (9.31-10.46)**	**0.05 (0.05-0.06)**	**60 (51-68)**	**0.8 (0.7-0.9)**	**1.47 (1.21-1.79)**	**0.20 (0.16-0.24)**	**3,625 (3,145-4,472)**	**12.2 (10.6-15.0)**	**2.00 (1.85-2.17)**	**0.07 (0.06-0.07)**
Brunei	3 (2-4)	0.6 (0.4-0.9)	0.02 (0.02-0.02)	0.05 (0.05-0.06)	0 (0-1)	1.3 (0.9-1.8)	0.01 (0.00-0.01)	0.17 (0.14-0.21)	1 (1-2)	10.6 (5.8-17.7)	0.00 (0.00-0.00)	0.05 (0.05-0.06)
Japan	3,077 (2,916-3,251)	2.4 (2.3-2.6)	7.78 (7.34-8.22)	0.06 (0.06-0.07)	47 (40-55)	0.9 (0.8-1.1)	1.12 (0.93-1.36)	0.22 (0.18-0.27)	2,750 (2,599-2,914)	11.1 (10.5-11.7)	1.74 (1.60-1.89)	0.07 (0.06-0.08)

Location	All ages				Children under 5				Adults over 70			
	Deaths (95% UI)	Deaths per 100,000 (95% UI)	Millions of episodes (95% UI)	Incidence per person (95% UI)	Deaths (95% UI)	Deaths per 100,000 (95% UI)	Millions of episodes (95% UI)	Incidence per person (95% UI)	Deaths (95% UI)	Deaths per 100,000 (95% UI)	Millions of episodes (95% UI)	Incidence per person (95% UI)
Singapore	30 (24-36)	0.8 (0.6-0.9)	0.18 (0.17-0.19)	0.05 (0.04-0.05)	1 (1-1)	0.4 (0.3-0.6)	0.03 (0.03-0.04)	0.17 (0.14-0.21)	22 (18-27)	6.7 (5.4-8.2)	0.02 (0.02-0.02)	0.05 (0.05-0.06)
South Korea	1,017 (516-2,000)	2.0 (1.0-4.0)	1.88 (1.75-2.03)	0.04 (0.03-0.04)	12 (8-17)	0.6 (0.4-0.8)	0.31 (0.25-0.38)	0.14 (0.12-0.18)	852 (422-1,708)	18.3 (9.1-36.7)	0.25 (0.22-0.28)	0.05 (0.05-0.06)
**Western Europe**	**14,686 (13,841-15,540)**	**3.4 (3.2-3.6)**	**65.41 (61.30-69.87)**	**0.15 (0.14-0.16)**	**152 (131-176)**	**0.7 (0.6-0.8)**	**16.60 (13.55-20.60)**	**0.76 (0.62-0.94)**	**13,405 (12,615-14,258)**	**22.3 (21.0-23.7)**	**15.54 (14.44-16.73)**	**0.26 (0.24-0.28)**
Andorra	1 (1-2)	1.8 (0.8-3.1)	0.01 (0.01-0.01)	0.15 (0.14-0.16)	0 (0-0)	0.6 (0.4-1.1)	0.00 (0.00-0.00)	0.80 (0.64-1.00)	1 (1-2)	11.8 (5.2-20.3)	0.00 (0.00-0.00)	0.24 (0.21-0.26)
Austria	68 (60-77)	0.8 (0.7-0.9)	1.68 (1.57-1.80)	0.19 (0.18-0.21)	2 (1-3)	0.5 (0.3-0.7)	0.43 (0.35-0.53)	1.07 (0.87-1.32)	58 (51-67)	4.8 (4.2-5.5)	0.30 (0.27-0.33)	0.25 (0.22-0.27)
Belgium	678 (586-782)	6.0 (5.2-6.9)	2.27 (2.12-2.45)	0.20 (0.19-0.22)	6 (4-8)	0.9 (0.7-1.2)	0.63 (0.51-0.79)	1.00 (0.80-1.24)	629 (537-734)	43.0 (36.8-50.2)	0.53 (0.49-0.58)	0.36 (0.33-0.40)
Cyprus	28 (16-46)	3.1 (1.8-5.1)	0.18 (0.16-0.19)	0.20 (0.18-0.21)	1 (1-1)	1.9 (1.2-2.9)	0.05 (0.04-0.07)	1.05 (0.86-1.32)	24 (13-41)	27.2 (15.0-46.0)	0.03 (0.02-0.03)	0.31 (0.28-0.35)
Denmark	313 (268-362)	5.5 (4.7-6.3)	1.10 (1.03-1.18)	0.19 (0.18-0.21)	3 (2-4)	1.0 (0.8-1.4)	0.27 (0.22-0.33)	0.93 (0.75-1.15)	287 (244-335)	39.5 (33.5-46.0)	0.25 (0.23-0.28)	0.35 (0.32-0.38)
Finland	53 (46-61)	1.0 (0.8-1.1)	1.37 (1.28-1.48)	0.25 (0.23-0.27)	0 (0-1)	0.2 (0.1-0.2)	0.37 (0.30-0.46)	1.28 (1.03-1.58)	48 (41-56)	6.4 (5.6-7.5)	0.25 (0.22-0.27)	0.33 (0.30-0.37)
France	3,111 (2,741-3,488)	4.8 (4.2-5.4)	10.06 (9.35-10.83)	0.15 (0.14-0.17)	42 (29-59)	1.1 (0.8-1.5)	2.68 (2.16-3.40)	0.68 (0.55-0.87)	2,809 (2,440-3,197)	33.1 (28.7-37.7)	2.49 (2.27-2.71)	0.29 (0.27-0.32)
Germany	4,859 (4,253-5,541)	5.9 (5.2-6.8)	15.98 (14.99-17.04)	0.19 (0.18-0.21)	20 (14-27)	0.6 (0.4-0.8)	3.42 (2.76-4.24)	1.00 (0.81-1.24)	4,475 (3,880-5,130)	32.7 (28.3-37.5)	4.72 (4.30-5.14)	0.35 (0.31-0.38)
Greece	28 (25-32)	0.3 (0.2-0.3)	1.46 (1.37-1.58)	0.13 (0.13-0.15)	1 (1-2)	0.2 (0.1-0.4)	0.35 (0.28-0.45)	0.75 (0.60-0.95)	23 (20-27)	1.4 (1.2-1.6)	0.30 (0.26-0.33)	0.17 (0.15-0.20)
Iceland	5 (4-5)	1.4 (1.3-1.6)	0.06 (0.06-0.06)	0.18 (0.17-0.20)	0 (0-0)	0.5 (0.4-0.8)	0.02 (0.01-0.02)	0.86 (0.70-1.09)	4 (4-5)	13.6 (11.8-15.6)	0.01 (0.01-0.01)	0.28 (0.25-0.30)
Ireland	32 (27-37)	0.7 (0.6-0.8)	0.81 (0.75-0.89)	0.18 (0.16-0.19)	2 (1-3)	0.6 (0.4-0.8)	0.28 (0.23-0.36)	0.84 (0.67-1.07)	25 (21-30)	6.1 (5.1-7.3)	0.09 (0.08-0.10)	0.21 (0.19-0.23)

Location	All ages Deaths (95% UI)	Deaths per 100,000 (95% UI)	Millions of episodes (95% UI)	Incidence per person (95% UI)	Children under 5 Deaths (95% UI)	Deaths per 100,000 (95% UI)	Millions of episodes (95% UI)	Incidence per person (95% UI)	Adults over 70 Deaths (95% UI)	Deaths per 100,000 (95% UI)	Millions of episodes (95% UI)	Incidence per person (95% UI)
Israel	260 (219-307)	3.2 (2.7-3.8)	0.96 (0.87-1.08)	0.12 (0.11-0.13)	8 (6-11)	1.0 (0.7-1.3)	0.39 (0.31-0.50)	0.46 (0.37-0.60)	224 (186-267)	36.4 (30.3-43.4)	0.14 (0.13-0.15)	0.23 (0.21-0.25)
Italy	529 (459-608)	0.9 (0.8-1.0)	4.63 (4.31-4.94)	0.08 (0.07-0.08)	11 (7-16)	0.4 (0.3-0.6)	1.25 (1.02-1.48)	0.49 (0.40-0.58)	482 (414-561)	4.9 (4.2-5.7)	1.12 (1.02-1.22)	0.11 (0.10-0.12)
Luxembourg	18 (15-20)	3.1 (2.7-3.5)	0.10 (0.09-0.11)	0.17 (0.16-0.18)	0 (0-0)	1.1 (0.7-1.5)	0.03 (0.02-0.03)	0.88 (0.72-1.07)	16 (13-18)	26.7 (22.8-30.9)	0.02 (0.02-0.02)	0.32 (0.29-0.35)
Malta	3 (2-3)	0.6 (0.5-0.8)	0.03 (0.03-0.03)	0.07 (0.06-0.07)	0 (0-0)	0.4 (0.3-0.6)	0.01 (0.01-0.01)	0.37 (0.30-0.47)	2 (2-3)	4.3 (3.5-5.1)	0.00 (0.00-0.01)	0.09 (0.08-0.10)
Netherlands	590 (520-664)	3.4 (3.0-3.9)	4.10 (3.82-4.41)	0.24 (0.22-0.26)	5 (3-7)	0.6 (0.4-0.8)	1.03 (0.82-1.30)	1.18 (0.94-1.49)	542 (475-616)	26.9 (23.6-30.5)	0.75 (0.69-0.83)	0.37 (0.34-0.41)
Norway	324 (280-373)	6.2 (5.3-7.1)	0.78 (0.72-0.84)	0.15 (0.14-0.16)	1 (1-2)	0.5 (0.3-0.7)	0.22 (0.18-0.27)	0.73 (0.59-0.90)	304 (261-352)	53.2 (45.7-61.6)	0.17 (0.16-0.19)	0.30 (0.28-0.33)
Portugal	158 (139-179)	1.5 (1.3-1.7)	0.93 (0.86-0.99)	0.09 (0.08-0.09)	4 (3-5)	0.9 (0.7-1.2)	0.23 (0.18-0.28)	0.54 (0.43-0.66)	136 (119-157)	8.8 (7.7-10.2)	0.21 (0.19-0.23)	0.14 (0.12-0.15)
Spain	869 (768-983)	1.9 (1.7-2.1)	5.32 (4.92-5.77)	0.11 (0.11-0.12)	11 (7-15)	0.5 (0.3-0.7)	1.29 (1.02-1.64)	0.59 (0.47-0.76)	798 (697-912)	12.5 (10.9-14.3)	1.21 (1.08-1.33)	0.19 (0.17-0.21)
Sweden	570 (501-652)	5.8 (5.1-6.6)	1.91 (1.78-2.05)	0.19 (0.18-0.21)	3 (2-4)	0.4 (0.3-0.6)	0.51 (0.42-0.65)	0.90 (0.73-1.13)	545 (477-624)	40.1 (35.1-45.9)	0.47 (0.43-0.51)	0.35 (0.32-0.38)
Switzerland	163 (129-200)	2.0 (1.5-2.4)	1.54 (1.44-1.65)	0.18 (0.17-0.20)	4 (3-6)	1.1 (0.7-1.5)	0.41 (0.34-0.50)	0.99 (0.81-1.21)	146 (115-180)	13.6 (10.7-16.8)	0.31 (0.28-0.34)	0.29 (0.26-0.31)
United Kingdom	2,028 (1,957-2,100)	3.1 (3.0-3.2)	10.06 (9.39-10.81)	0.15 (0.14-0.17)	28 (25-31)	0.7 (0.6-0.8)	2.72 (2.21-3.37)	0.70 (0.57-0.86)	1,825 (1,759-1,894)	22.9 (22.1-23.8)	2.16 (2.00-2.33)	0.27 (0.25-0.29)
England	1,731 (1,672-1,794)	3.1 (3.0-3.3)	8.29 (7.75-8.91)	0.15 (0.14-0.16)	20 (18-22)	0.6 (0.6-0.7)	2.23 (1.82-2.78)	0.67 (0.55-0.84)	1,566 (1,510-1,627)	23.5 (22.7-24.4)	1.81 (1.68-1.96)	0.27 (0.25-0.29)
Northern Ireland	37 (32-44)	2.0 (1.7-2.4)	0.28 (0.26-0.30)	0.15 (0.14-0.16)	2 (1-3)	1.5 (0.9-2.4)	0.09 (0.07-0.11)	0.76 (0.61-0.94)	32 (27-38)	15.6 (13.2-18.6)	0.04 (0.04-0.05)	0.22 (0.20-0.24)
Scotland	150 (131-171)	2.8 (2.4-3.2)	0.76 (0.71-0.82)	0.14 (0.13-0.15)	3 (2-5)	1.1 (0.7-1.6)	0.20 (0.16-0.26)	0.71 (0.58-0.90)	131 (113-152)	19.3 (16.7-22.4)	0.16 (0.14-0.17)	0.23 (0.21-0.25)

Location	All ages Deaths (95% UI)	All ages Deaths per 100,000 (95% UI)	All ages Millions of episodes (95% UI)	All ages Incidence per person (95% UI)	Children under 5 Deaths (95% UI)	Children under 5 Deaths per 100,000 (95% UI)	Children under 5 Millions of episodes (95% UI)	Children under 5 Incidence per person (95% UI)	Adults over 70 Deaths (95% UI)	Adults over 70 Deaths per 100,000 (95% UI)	Adults over 70 Millions of episodes (95% UI)	Adults over 70 Incidence per person (95% UI)
Wales	109 (95-126)	3.5 (3.1-4.0)	0.73 (0.68-0.78)	0.23 (0.22-0.25)	3 (2-4)	1.7 (1.1-2.4)	0.19 (0.16-0.24)	1.14 (0.92-1.41)	96 (82-112)	22.7 (19.4-26.5)	0.15 (0.13-0.16)	0.35 (0.32-0.38)
**Southern Latin America**	**1,214 (1,105-1,329)**	**1.9 (1.7-2.0)**	**15.27 (13.99-16.70)**	**0.23 (0.21-0.26)**	**144 (113-184)**	**2.9 (2.2-3.7)**	**6.03 (4.93-7.37)**	**1.20 (0.98-1.47)**	**862 (770-965)**	**17.1 (15.3-19.1)**	**0.81 (0.74-0.88)**	**0.16 (0.15-0.17)**
Argentina	711 (643-789)	1.6 (1.5-1.8)	11.67 (10.58-12.81)	0.27 (0.24-0.29)	127 (95-166)	3.5 (2.7-4.6)	4.84 (3.96-5.93)	1.35 (1.11-1.66)	454 (397-519)	13.7 (12.0-15.7)	0.53 (0.48-0.58)	0.16 (0.15-0.18)
Chile	356 (283-441)	2.0 (1.6-2.4)	3.32 (3.07-3.59)	0.18 (0.17-0.20)	9 (7-13)	0.8 (0.5-1.1)	1.08 (0.90-1.32)	0.90 (0.74-1.09)	290 (227-363)	20.9 (16.3-26.2)	0.24 (0.22-0.27)	0.17 (0.16-0.19)
Uruguay	146 (129-165)	4.3 (3.8-4.8)	0.28 (0.26-0.31)	0.08 (0.07-0.09)	8 (5-12)	3.2 (2.0-5.2)	0.11 (0.09-0.14)	0.46 (0.37-0.59)	119 (103-136)	33.1 (28.7-37.8)	0.04 (0.03-0.04)	0.10 (0.09-0.11)
**Central Europe, Eastern Europe, and Central Asia**	**3,372 (2,808-4,147)**	**0.8 (0.7-1.0)**	**163.54 (154.19-174.38)**	**0.39 (0.37-0.42)**	**1,943 (1,411-2,716)**	**6.9 (5.0-9.6)**	**50.88 (43.96-58.90)**	**1.80 (1.56-2.09)**	**777 (700-866)**	**2.1 (1.9-2.3)**	**15.96 (14.41-17.72)**	**0.43 (0.39-0.48)**
**Eastern Europe**	**550 (456-655)**	**0.3 (0.2-0.3)**	**79.91 (75.38-85.16)**	**0.38 (0.36-0.40)**	**180 (129-246)**	**1.4 (1.0-1.9)**	**23.66 (20.85-27.18)**	**1.83 (1.61-2.10)**	**166 (134-207)**	**0.8 (0.6-1.0)**	**7.88 (6.98-8.84)**	**0.38 (0.34-0.43)**
Belarus	14 (12-17)	0.1 (0.1-0.2)	3.18 (2.96-3.44)	0.33 (0.31-0.36)	2 (1-4)	0.4 (0.2-0.6)	0.99 (0.82-1.22)	1.71 (1.42-2.11)	7 (6-9)	0.8 (0.6-1.0)	0.31 (0.27-0.35)	0.32 (0.29-0.37)
Estonia	2 (2-3)	0.2 (0.1-0.2)	0.99 (0.93-1.05)	0.75 (0.71-0.80)	0 (0-0)	0.3 (0.2-0.5)	0.25 (0.21-0.28)	3.61 (3.10-4.02)	1 (1-2)	0.8 (0.6-0.9)	0.15 (0.14-0.17)	0.83 (0.73-0.94)
Latvia	4 (4-5)	0.2 (0.2-0.2)	1.11 (1.03-1.20)	0.56 (0.52-0.60)	1 (0-1)	0.5 (0.3-0.8)	0.34 (0.28-0.41)	3.21 (2.67-3.83)	2 (2-3)	0.8 (0.6-0.9)	0.15 (0.13-0.17)	0.52 (0.46-0.58)
Lithuania	9 (8-10)	0.3 (0.3-0.4)	1.89 (1.77-2.03)	0.65 (0.61-0.70)	1 (1-1)	0.6 (0.4-0.9)	0.54 (0.45-0.65)	3.61 (3.02-4.30)	5 (4-5)	1.1 (0.9-1.3)	0.27 (0.23-0.30)	0.64 (0.56-0.72)
Moldova	9 (6-13)	0.2 (0.2-0.3)	1.03 (0.96-1.12)	0.25 (0.24-0.27)	5 (3-9)	2.4 (1.4-4.0)	0.34 (0.28-0.41)	1.50 (1.25-1.81)	2 (2-2)	0.7 (0.5-0.8)	0.07 (0.06-0.08)	0.25 (0.22-0.28)
Russia	439 (347-545)	0.3 (0.2-0.4)	52.50 (49.40-55.93)	0.36 (0.34-0.38)	150 (106-218)	1.6 (1.1-2.3)	15.89 (14.18-18.18)	1.69 (1.51-1.94)	115 (85-154)	0.9 (0.6-1.2)	4.82 (4.23-5.43)	0.36 (0.32-0.41)
Ukraine	72 (55-95)	0.2 (0.1-0.2)	19.20 (17.94-20.62)	0.42 (0.39-0.45)	21 (9-41)	0.8 (0.4-1.7)	5.32 (4.43-6.33)	2.17 (1.80-2.58)	33 (25-42)	0.6 (0.5-0.8)	2.11 (1.84-2.39)	0.40 (0.35-0.45)

Location	All ages Deaths (95% UI)	All ages Deaths per 100,000 (95% UI)	All ages Millions of episodes (95% UI)	All ages Incidence per person (95% UI)	Children under 5 Deaths (95% UI)	Children under 5 Deaths per 100,000 (95% UI)	Children under 5 Millions of episodes (95% UI)	Children under 5 Incidence per person (95% UI)	Adults over 70 Deaths (95% UI)	Adults over 70 Deaths per 100,000 (95% UI)	Adults over 70 Millions of episodes (95% UI)	Adults over 70 Incidence per person (95% UI)
**Central Europe**	**785 (713-857)**	**0.7 (0.6-0.7)**	**47.62 (44.63-50.85)**	**0.41 (0.39-0.44)**	**73 (60-88)**	**1.3 (1.1-1.6)**	**14.20 (11.98-17.03)**	**2.54 (2.14-3.05)**	**548 (494-608)**	**4.2 (3.8-4.7)**	**7.01 (6.42-7.71)**	**0.54 (0.50-0.60)**
Albania	7 (5-13)	0.3 (0.2-0.4)	0.94 (0.86-1.03)	0.32 (0.30-0.36)	2 (1-3)	0.9 (0.4-1.5)	0.35 (0.28-0.43)	1.86 (1.50-2.31)	4 (2-7)	1.4 (0.8-2.7)	0.09 (0.08-0.10)	0.36 (0.31-0.40)
Bosnia and Herzegovina	8 (5-12)	0.2 (0.1-0.3)	1.06 (0.99-1.15)	0.28 (0.26-0.30)	3 (2-4)	1.7 (1.1-2.4)	0.30 (0.24-0.36)	1.73 (1.41-2.09)	4 (1-7)	0.9 (0.4-1.7)	0.14 (0.12-0.16)	0.35 (0.31-0.40)
Bulgaria	25 (21-31)	0.3 (0.3-0.4)	2.49 (2.32-2.71)	0.34 (0.32-0.37)	7 (4-12)	2.2 (1.2-3.7)	0.73 (0.60-0.91)	2.20 (1.80-2.72)	9 (7-11)	0.9 (0.8-1.1)	0.40 (0.35-0.45)	0.41 (0.36-0.46)
Croatia	23 (20-27)	0.6 (0.5-0.6)	1.77 (1.68-1.87)	0.42 (0.40-0.44)	1 (1-2)	0.6 (0.4-0.8)	0.55 (0.48-0.62)	2.79 (2.44-3.13)	16 (13-19)	2.9 (2.4-3.4)	0.29 (0.26-0.31)	0.51 (0.47-0.56)
Czech Republic	197 (176-224)	1.9 (1.7-2.1)	5.18 (4.86-5.53)	0.49 (0.46-0.52)	3 (2-5)	0.6 (0.4-0.8)	1.39 (1.19-1.66)	2.56 (2.19-3.05)	162 (141-187)	12.9 (11.2-14.9)	1.03 (0.95-1.13)	0.82 (0.75-0.90)
Hungary	202 (173-230)	2.0 (1.8-2.3)	4.37 (4.08-4.68)	0.44 (0.41-0.47)	4 (3-6)	1.0 (0.6-1.4)	1.06 (0.87-1.31)	2.35 (1.93-2.90)	156 (133-181)	12.6 (10.7-14.7)	0.93 (0.85-1.02)	0.75 (0.68-0.83)
Macedonia	9 (5-15)	0.4 (0.3-0.7)	0.64 (0.60-0.69)	0.31 (0.29-0.33)	3 (2-5)	2.7 (1.5-4.8)	0.21 (0.18-0.25)	1.86 (1.58-2.22)	4 (2-7)	2.1 (1.2-4.1)	0.06 (0.06-0.07)	0.38 (0.33-0.43)
Montenegro	1 (0-1)	0.1 (0.1-0.2)	0.15 (0.13-0.16)	0.23 (0.22-0.25)	0 (0-0)	0.7 (0.4-1.2)	0.05 (0.04-0.06)	1.27 (1.07-1.53)	0 (0-0)	0.5 (0.2-0.8)	0.02 (0.01-0.02)	0.27 (0.24-0.30)
Poland	173 (152-198)	0.4 (0.4-0.5)	16.58 (15.35-17.98)	0.43 (0.40-0.47)	7 (4-11)	0.4 (0.2-0.6)	5.16 (4.23-6.39)	2.76 (2.26-3.42)	128 (109-151)	3.2 (2.7-3.8)	2.07 (1.86-2.32)	0.52 (0.47-0.59)
Romania	75 (64-90)	0.4 (0.3-0.5)	8.05 (7.51-8.69)	0.42 (0.39-0.45)	34 (23-48)	3.9 (2.7-5.4)	2.33 (1.93-2.82)	2.65 (2.20-3.21)	26 (22-30)	1.1 (0.9-1.3)	1.15 (1.02-1.30)	0.49 (0.44-0.56)
Serbia	35 (15-70)	0.4 (0.2-0.8)	3.00 (2.84-3.16)	0.34 (0.32-0.36)	5 (3-7)	1.2 (0.8-1.7)	0.93 (0.83-1.04)	2.25 (2.01-2.51)	22 (7-52)	2.3 (0.7-5.3)	0.42 (0.38-0.46)	0.43 (0.39-0.47)
Slovakia	22 (12-32)	0.4 (0.2-0.6)	2.47 (2.28-2.68)	0.45 (0.42-0.49)	3 (2-5)	1.1 (0.7-1.8)	0.82 (0.67-1.00)	2.86 (2.33-3.48)	12 (5-20)	2.3 (1.1-3.8)	0.28 (0.24-0.31)	0.54 (0.48-0.60)
Slovenia	7 (6-8)	0.3 (0.3-0.4)	0.93 (0.86-1.00)	0.45 (0.42-0.49)	0 (0-0)	0.2 (0.1-0.3)	0.31 (0.26-0.37)	2.96 (2.42-3.54)	5 (5-6)	2.0 (1.7-2.3)	0.14 (0.12-0.16)	0.51 (0.46-0.57)

Location	All ages				Children under 5				Adults over 70			
	Deaths (95% UI)	Deaths per 100,000 (95% UI)	Millions of episodes (95% UI)	Incidence per person (95% UI)	Deaths (95% UI)	Deaths per 100,000 (95% UI)	Millions of episodes (95% UI)	Incidence per person (95% UI)	Deaths (95% UI)	Deaths per 100,000 (95% UI)	Millions of episodes (95% UI)	Incidence per person (95% UI)
**Central Asia**	**2,037 (1,496-2,806)**	**2.3 (1.7-3.2)**	**36.01 (33.38-39.09)**	**0.41 (0.38-0.44)**	**1,690 (1,171-2,454)**	**17.5 (12.1-25.4)**	**13.02 (11.02-15.43)**	**1.35 (1.14-1.60)**	**64 (44-87)**	**1.9 (1.3-2.6)**	**1.07 (0.95-1.19)**	**0.32 (0.29-0.36)**
Armenia	15 (12-20)	0.5 (0.4-0.7)	1.37 (1.28-1.48)	0.45 (0.42-0.49)	8 (5-13)	3.7 (2.3-5.9)	0.37 (0.31-0.43)	1.65 (1.39-1.96)	3 (2-3)	1.3 (1.1-1.5)	0.09 (0.08-0.11)	0.41 (0.36-0.46)
Azerbaijan	199 (113-333)	2.0 (1.1-3.4)	5.32 (4.91-5.76)	0.54 (0.50-0.59)	176 (92-311)	18.4 (9.6-32.5)	1.78 (1.51-2.13)	1.86 (1.57-2.23)	5 (3-9)	1.3 (0.7-2.3)	0.18 (0.16-0.21)	0.45 (0.40-0.51)
Georgia	21 (16-28)	0.5 (0.4-0.7)	1.45 (1.35-1.57)	0.36 (0.34-0.39)	11 (6-18)	3.3 (1.9-5.4)	0.46 (0.38-0.55)	1.37 (1.13-1.65)	5 (4-6)	1.3 (1.1-1.6)	0.13 (0.11-0.14)	0.31 (0.28-0.35)
Kazakhstan	78 (55-111)	0.4 (0.3-0.6)	6.54 (6.05-7.08)	0.37 (0.34-0.40)	59 (35-92)	3.0 (1.8-4.7)	2.18 (1.83-2.59)	1.11 (0.94-1.32)	6 (5-7)	0.7 (0.6-0.9)	0.24 (0.21-0.27)	0.29 (0.26-0.33)
Kyrgyzstan	208 (158-272)	3.5 (2.6-4.6)	2.48 (2.30-2.68)	0.41 (0.38-0.45)	187 (137-253)	25.0 (18.3-33.8)	0.94 (0.83-1.10)	1.26 (1.10-1.47)	3 (3-4)	2.1 (1.8-2.4)	0.05 (0.05-0.06)	0.31 (0.28-0.35)
Mongolia	4 (3-6)	0.1 (0.1-0.2)	1.36 (1.26-1.49)	0.45 (0.42-0.49)	2 (1-3)	0.5 (0.3-0.9)	0.57 (0.49-0.68)	1.54 (1.32-1.82)	0 (0-1)	0.5 (0.2-1.1)	0.03 (0.02-0.03)	0.32 (0.28-0.37)
Tajikistan	1,205 (717-1,961)	14.0 (8.3-22.8)	5.38 (4.94-5.91)	0.63 (0.57-0.69)	1,009 (527-1,744)	88.6 (46.3-153.2)	2.36 (2.00-2.79)	2.07 (1.75-2.45)	29 (11-51)	16.8 (6.5-29.1)	0.08 (0.07-0.09)	0.47 (0.42-0.53)
Turkmenistan	126 (79-202)	2.3 (1.4-3.7)	3.50 (3.21-3.79)	0.64 (0.59-0.69)	109 (61-184)	18.8 (10.6-31.9)	1.20 (1.00-1.43)	2.08 (1.74-2.48)	3 (2-3)	1.9 (1.6-2.3)	0.07 (0.06-0.08)	0.48 (0.43-0.54)
Uzbekistan	179 (113-279)	0.6 (0.4-0.9)	8.61 (7.89-9.43)	0.28 (0.26-0.31)	129 (62-229)	3.9 (1.9-6.9)	3.17 (2.59-3.85)	0.95 (0.78-1.15)	9 (7-11)	1.0 (0.8-1.2)	0.21 (0.18-0.23)	0.22 (0.20-0.25)
**Latin America and Caribbean**	**24,026 (21,716-27,907)**	**4.2 (3.8-4.8)**	**455.08 (430.80-481.24)**	**0.79 (0.75-0.83)**	**8,828 (7,589-10,522)**	**17.8 (15.3-21.2)**	**140.03 (123.90-157.52)**	**2.82 (2.50-3.18)**	**8,830 (7,885-10,715)**	**32.1 (28.6-38.9)**	**44.37 (41.36-47.99)**	**1.61 (1.50-1.74)**
**Central Latin America**	**10,603 (9,827-11,651)**	**4.2 (3.9-4.6)**	**145.54 (136.22-155.31)**	**0.57 (0.54-0.61)**	**3,552 (3,154-4,093)**	**15.6 (13.8-17.9)**	**42.75 (37.08-49.85)**	**1.87 (1.62-2.18)**	**3,904 (3,553-4,426)**	**35.6 (32.4-40.4)**	**11.22 (10.37-12.21)**	**1.02 (0.95-1.11)**
Colombia	703 (603-810)	1.4 (1.2-1.7)	29.20 (27.23-31.34)	0.60 (0.56-0.65)	217 (150-303)	6.1 (4.2-8.5)	6.48 (5.41-7.82)	1.81 (1.51-2.19)	321 (272-377)	14.7 (12.5-17.3)	2.18 (1.97-2.40)	1.00 (0.91-1.10)
Costa Rica	93 (82-106)	1.9 (1.7-2.2)	3.92 (3.66-4.21)	0.81 (0.76-0.87)	11 (6-20)	3.8 (2.1-6.4)	0.77 (0.65-0.94)	2.54 (2.13-3.08)	59 (51-68)	20.6 (17.6-23.8)	0.40 (0.36-0.44)	1.38 (1.25-1.53)

Location	All ages				Children under 5				Adults over 70			
	Deaths (95% UI)	Deaths per 100,000 (95% UI)	Millions of episodes (95% UI)	Incidence per person (95% UI)	Deaths (95% UI)	Deaths per 100,000 (95% UI)	Millions of episodes (95% UI)	Incidence per person (95% UI)	Deaths (95% UI)	Deaths per 100,000 (95% UI)	Millions of episodes (95% UI)	Incidence per person (95% UI)
El Salvador	292 (178-524)	4.7 (2.9-8.5)	5.50 (5.12-5.93)	0.89 (0.83-0.96)	67 (38-112)	12.6 (7.1-21.0)	1.41 (1.17-1.71)	2.64 (2.19-3.19)	141 (72-289)	40.3 (20.6-82.4)	0.66 (0.60-0.74)	1.89 (1.70-2.10)
Guatemala	2,540 (2,115-2,998)	15.4 (12.8-18.1)	15.46 (14.65-16.31)	0.94 (0.89-0.99)	980 (765-1,236)	49.7 (38.8-62.7)	6.32 (5.74-6.82)	3.20 (2.91-3.46)	630 (495-788)	119.9 (94.2-149.8)	0.94 (0.86-1.04)	1.79 (1.64-1.98)
Honduras	977 (569-1,566)	11.7 (6.8-18.8)	7.29 (6.82-7.77)	0.88 (0.82-0.93)	264 (165-392)	27.9 (17.5-41.4)	2.65 (2.28-2.99)	2.80 (2.41-3.16)	386 (170-736)	145.2 (63.8-276.8)	0.51 (0.45-0.57)	1.92 (1.70-2.14)
Mexico	4,425 (4,216-4,678)	3.4 (3.3-3.6)	39.24 (36.35-42.67)	0.30 (0.28-0.33)	1,365 (1,192-1,596)	11.7 (10.2-13.7)	13.58 (11.32-16.60)	1.16 (0.97-1.42)	1,834 (1,746-1,936)	32.4 (30.9-34.3)	3.36 (3.11-3.64)	0.59 (0.55-0.64)
Nicaragua	231 (159-356)	3.7 (2.6-5.8)	4.24 (3.96-4.54)	0.69 (0.64-0.74)	117 (79-165)	19.2 (13.0-27.0)	1.30 (1.12-1.51)	2.13 (1.84-2.48)	63 (35-125)	28.9 (15.9-57.1)	0.31 (0.27-0.34)	1.39 (1.24-1.57)
Panama	179 (146-220)	4.5 (3.7-5.5)	3.64 (3.40-3.91)	0.92 (0.86-0.98)	82 (55-120)	23.7 (15.8-34.5)	1.02 (0.87-1.20)	2.93 (2.50-3.47)	48 (40-58)	23.2 (19.2-28.1)	0.32 (0.29-0.35)	1.54 (1.40-1.69)
Venezuela	1,164 (996-1,365)	3.7 (3.2-4.3)	37.04 (34.33-39.85)	1.18 (1.09-1.27)	447 (320-616)	15.5 (11.1-21.4)	9.22 (7.83-10.87)	3.19 (2.71-3.77)	421 (349-503)	33.0 (27.4-39.5)	2.54 (2.31-2.82)	2.00 (1.81-2.22)
**Andean Latin America**	**1,898 (1,387-2,873)**	**3.2 (2.3-4.8)**	**51.85 (48.73-55.06)**	**0.87 (0.81-0.92)**	**733 (554-950)**	**11.0 (8.3-14.3)**	**15.44 (13.32-17.62)**	**2.32 (2.00-2.64)**	**662 (395-1,168)**	**24.7 (14.8-43.7)**	**4.72 (4.32-5.18)**	**1.76 (1.61-1.94)**
Bolivia	716 (452-1,096)	6.5 (4.1-9.9)	11.50 (10.65-12.47)	1.04 (0.96-1.13)	311 (179-496)	22.7 (13.1-36.3)	3.74 (3.10-4.55)	2.73 (2.27-3.33)	227 (116-440)	46.8 (23.8-90.7)	1.07 (0.96-1.19)	2.20 (1.97-2.46)
Ecuador	353 (298-418)	2.1 (1.8-2.5)	13.98 (13.12-14.92)	0.84 (0.79-0.90)	157 (108-220)	8.8 (6.1-12.3)	4.25 (3.59-4.91)	2.37 (2.01-2.75)	106 (92-123)	14.3 (12.4-16.4)	1.10 (1.02-1.20)	1.48 (1.36-1.61)
Peru	830 (549-1,412)	2.6 (1.7-4.4)	26.37 (24.84-27.90)	0.82 (0.77-0.87)	265 (175-387)	7.5 (5.0-11.0)	7.45 (6.53-8.37)	2.13 (1.86-2.39)	328 (173-649)	22.7 (11.9-44.9)	2.55 (2.29-2.84)	1.76 (1.59-1.96)
**Caribbean**	**5,135 (3,568-7,476)**	**11.2 (7.8-16.3)**	**34.98 (33.01-37.09)**	**0.76 (0.72-0.81)**	**2,773 (1,724-4,440)**	**69.5 (43.2-111.3)**	**9.16 (7.92-10.58)**	**2.30 (1.98-2.65)**	**1,180 (717-2,001)**	**41.1 (25.0-69.8)**	**4.15 (3.81-4.56)**	**1.45 (1.33-1.59)**
Antigua and Barbuda	1 (1-1)	1.4 (1.2-1.6)	0.06 (0.06-0.07)	0.70 (0.66-0.75)	0 (0-0)	5.2 (3.1-8.0)	0.01 (0.01-0.01)	1.79 (1.50-2.13)	1 (1-1)	12.7 (10.7-14.9)	0.01 (0.01-0.01)	1.35 (1.21-1.48)

Location	All ages				Children under 5				Adults over 70			
	Deaths (95% UI)	Deaths per 100,000 (95% UI)	Millions of episodes (95% UI)	Incidence per person (95% UI)	Deaths (95% UI)	Deaths per 100,000 (95% UI)	Millions of episodes (95% UI)	Incidence per person (95% UI)	Deaths (95% UI)	Deaths per 100,000 (95% UI)	Millions of episodes (95% UI)	Incidence per person (95% UI)
The Bahamas	5 (4-6)	1.2 (0.9-1.6)	0.29 (0.27-0.31)	0.73 (0.68-0.78)	1 (1-3)	3.9 (1.6-8.8)	0.06 (0.05-0.08)	1.94 (1.60-2.33)	2 (2-2)	9.2 (7.7-10.9)	0.03 (0.02-0.03)	1.22 (1.09-1.36)
Barbados	4 (4-5)	1.4 (1.3-1.7)	0.22 (0.21-0.24)	0.79 (0.74-0.85)	0 (0-1)	3.1 (1.3-6.5)	0.03 (0.03-0.04)	2.03 (1.70-2.47)	3 (2-3)	9.5 (8.2-11.2)	0.04 (0.03-0.04)	1.28 (1.15-1.44)
Belize	9 (6-14)	2.4 (1.7-3.7)	0.28 (0.26-0.30)	0.74 (0.69-0.80)	4 (2-9)	9.1 (3.7-19.0)	0.09 (0.07-0.10)	1.81 (1.56-2.14)	2 (2-3)	25.8 (21.6-30.8)	0.01 (0.01-0.01)	1.54 (1.38-1.72)
Bermuda	0 (0-0)	0.5 (0.5-0.6)	0.05 (0.05-0.06)	0.76 (0.71-0.82)	0 (0-0)	0.7 (0.4-1.3)	0.01 (0.01-0.01)	2.14 (1.73-2.63)	0 (0-0)	5.7 (4.7-6.7)	0.01 (0.01-0.01)	1.33 (1.20-1.47)
Cuba	264 (235-297)	2.3 (2.1-2.6)	6.52 (6.16-6.91)	0.57 (0.54-0.61)	11 (8-15)	1.9 (1.4-2.5)	0.78 (0.66-0.94)	1.30 (1.10-1.56)	170 (146-195)	14.9 (12.8-17.1)	1.36 (1.23-1.49)	1.19 (1.08-1.30)
Dominica	1 (1-2)	1.7 (1.4-2.1)	0.05 (0.05-0.06)	0.73 (0.69-0.78)	0 (0-1)	6.5 (3.4-10.9)	0.01 (0.01-0.01)	1.95 (1.63-2.37)	1 (1-1)	13.5 (11.4-16.2)	0.01 (0.01-0.01)	1.31 (1.18-1.46)
Dominican Republic	465 (319-743)	4.4 (3.0-7.1)	8.29 (7.73-8.89)	0.79 (0.73-0.84)	211 (130-320)	22.4 (13.8-34.0)	2.31 (1.94-2.73)	2.45 (2.07-2.90)	153 (87-316)	32.0 (18.1-65.9)	0.83 (0.75-0.93)	1.74 (1.56-1.95)
Grenada	2 (1-2)	1.5 (1.3-1.8)	0.12 (0.11-0.12)	1.08 (1.01-1.17)	0 (0-1)	4.1 (2.0-7.8)	0.02 (0.02-0.03)	2.59 (2.12-3.16)	1 (1-1)	14.1 (12.0-16.5)	0.01 (0.01-0.01)	1.78 (1.60-1.99)
Guyana	51 (39-66)	6.7 (5.1-8.7)	0.48 (0.45-0.51)	0.62 (0.58-0.66)	18 (11-26)	27.5 (16.6-40.5)	0.11 (0.09-0.12)	1.63 (1.40-1.91)	12 (9-16)	53.7 (39.2-71.6)	0.03 (0.03-0.04)	1.48 (1.33-1.66)
Haiti	4,117 (2,648-6,388)	37.0 (23.8-57.4)	12.01 (11.20-12.91)	1.08 (1.01-1.16)	2,479 (1,447-4,146)	163.1 (95.2-272.8)	4.51 (3.92-5.19)	2.97 (2.58-3.41)	724 (329-1,416)	223.2 (101.5-436.8)	0.76 (0.67-0.85)	2.34 (2.06-2.63)
Jamaica	73 (40-148)	2.5 (1.4-5.2)	1.36 (1.28-1.46)	0.47 (0.45-0.51)	22 (10-43)	7.9 (3.8-15.4)	0.34 (0.28-0.40)	1.22 (1.03-1.43)	34 (15-81)	19.1 (8.6-45.2)	0.19 (0.17-0.22)	1.07 (0.96-1.20)
Puerto Rico	72 (64-81)	2.0 (1.7-2.2)	2.53 (2.39-2.69)	0.69 (0.65-0.73)	4 (3-6)	2.1 (1.4-2.9)	0.37 (0.31-0.46)	1.78 (1.48-2.18)	49 (42-57)	13.3 (11.4-15.3)	0.50 (0.46-0.56)	1.36 (1.23-1.50)
Saint Lucia	3 (2-3)	1.6 (1.3-1.9)	0.21 (0.20-0.23)	1.16 (1.08-1.24)	1 (0-1)	5.2 (2.3-10.2)	0.03 (0.02-0.03)	2.90 (2.46-3.44)	1 (1-2)	12.8 (10.9-15.1)	0.02 (0.02-0.03)	2.01 (1.79-2.22)

Location	All ages Deaths (95% UI)	All ages Deaths per 100,000 (95% UI)	All ages Millions of episodes (95% UI)	All ages Incidence per person (95% UI)	Children under 5 Deaths (95% UI)	Children under 5 Deaths per 100,000 (95% UI)	Children under 5 Millions of episodes (95% UI)	Children under 5 Incidence per person (95% UI)	Adults over 70 Deaths (95% UI)	Adults over 70 Deaths per 100,000 (95% UI)	Adults over 70 Millions of episodes (95% UI)	Adults over 70 Incidence per person (95% UI)
Saint Vincent and the Grenadines	3 (3-4)	3.0 (2.5-3.6)	0.08 (0.07-0.08)	0.71 (0.67-0.76)	1 (0-1)	9.4 (5.1-15.9)	0.02 (0.01-0.02)	1.76 (1.49-2.11)	1 (1-2)	26.7 (22.8-31.4)	0.01 (0.01-0.01)	1.49 (1.35-1.68)
Suriname	38 (25-60)	7.0 (4.6-11.0)	0.41 (0.39-0.44)	0.76 (0.71-0.81)	16 (8-28)	34.6 (18.1-60.0)	0.10 (0.08-0.12)	2.07 (1.74-2.48)	13 (7-24)	52.0 (29.0-95.5)	0.04 (0.04-0.05)	1.80 (1.61-2.01)
Trinidad and Tobago	25 (21-30)	1.8 (1.5-2.2)	0.62 (0.59-0.67)	0.47 (0.44-0.50)	4 (1-9)	5.5 (1.9-12.8)	0.09 (0.08-0.11)	1.36 (1.13-1.64)	12 (10-14)	15.0 (12.6-17.7)	0.08 (0.07-0.09)	0.95 (0.86-1.06)
Virgin Islands, U.S.	1 (1-2)	1.2 (0.7-2.1)	0.09 (0.08-0.09)	0.81 (0.76-0.87)	0 (0-0)	1.3 (0.8-1.9)	0.01 (0.01-0.01)	2.00 (1.65-2.46)	1 (0-1)	6.9 (3.9-12.0)	0.02 (0.02-0.02)	1.39 (1.23-1.55)
**Tropical Latin America**	**6,196 (5,911-6,544)**	**2.9 (2.7-3.0)**	**222.71 (211.37-234.91)**	**1.03 (0.98-1.09)**	**1,686 (1,485-1,927)**	**10.5 (9.2-12.0)**	**72.69 (65.26-80.12)**	**4.51 (4.05-4.97)**	**3,026 (2,870-3,189)**	**27.4 (26.0-28.9)**	**24.28 (22.63-26.09)**	**2.20 (2.05-2.37)**
Brazil	5,995 (5,728-6,319)	2.9 (2.7-3.0)	212.43 (201.67-223.75)	1.01 (0.96-1.07)	1,611 (1,416-1,845)	10.4 (9.1-11.9)	69.48 (62.44-76.56)	4.49 (4.03-4.95)	2,945 (2,795-3,086)	27.4 (26.0-28.7)	23.41 (21.81-25.13)	2.17 (2.03-2.33)
Paraguay	201 (133-309)	3.0 (2.0-4.6)	10.28 (9.57-11.06)	1.54 (1.43-1.65)	75 (48-112)	11.9 (7.6-17.7)	3.22 (2.78-3.56)	5.10 (4.40-5.64)	81 (44-152)	30.7 (16.6-57.5)	0.87 (0.77-0.97)	3.29 (2.91-3.66)
**Southeast Asia, East Asia, and Oceania**	**82,391 (52,849-114,890)**	**4.0 (2.5-5.5)**	**777.37 (732.38-826.21)**	**0.37 (0.35-0.40)**	**15,443 (13,267-18,208)**	**12.5 (10.8-14.8)**	**128.88 (108.56-154.17)**	**1.05 (0.88-1.25)**	**38,425 (19,341-56,971)**	**34.1 (17.2-50.6)**	**57.14 (51.36-63.76)**	**0.51 (0.46-0.57)**
**East Asia**	**6,443 (4,668-10,215)**	**0.5 (0.3-0.7)**	**292.85 (273.85-313.09)**	**0.21 (0.19-0.22)**	**1,988 (1,573-2,596)**	**3.1 (2.4-4.0)**	**35.28 (29.30-43.26)**	**0.55 (0.45-0.67)**	**2,351 (1,415-4,440)**	**2.7 (1.6-5.1)**	**24.64 (21.89-27.81)**	**0.28 (0.25-0.32)**
China	5,544 (3,952-9,211)	0.4 (0.3-0.7)	260.40 (242.82-278.68)	0.19 (0.18-0.20)	1,342 (1,102-1,659)	2.2 (1.8-2.7)	28.66 (23.75-35.17)	0.47 (0.39-0.58)	2,209 (1,333-4,215)	2.6 (1.6-5.0)	22.12 (19.62-25.04)	0.26 (0.23-0.30)
North Korea	784 (462-1,301)	3.0 (1.7-4.9)	21.43 (19.93-23.07)	0.81 (0.75-0.87)	637 (331-1,153)	21.5 (11.2-38.9)	5.48 (4.55-6.50)	1.85 (1.54-2.20)	72 (35-148)	4.6 (2.2-9.5)	1.34 (1.19-1.52)	0.86 (0.76-0.97)
Taiwan	114 (57-173)	0.5 (0.2-0.7)	11.02 (10.33-11.75)	0.46 (0.44-0.50)	9 (6-14)	0.9 (0.6-1.3)	1.14 (0.93-1.41)	1.13 (0.92-1.39)	70 (32-113)	3.4 (1.6-5.6)	1.18 (1.05-1.31)	0.58 (0.52-0.65)
**Southeast Asia**	**73,484 (46,195-101,156)**	**11.2 (7.1-15.4)**	**471.27 (444.25-499.81)**	**0.72 (0.68-0.76)**	**13,027 (10,999-15,526)**	**22.8 (19.3-27.2)**	**91.01 (76.83-108.32)**	**1.60 (1.35-1.90)**	**35,063 (17,323-53,566)**	**140.7 (69.5-214.9)**	**31.90 (28.86-35.41)**	**1.28 (1.16-1.42)**
Cambodia	1,349 (834-2,090)	8.5 (5.2-13.1)	14.09 (13.18-15.05)	0.88 (0.83-0.94)	321 (199-509)	17.0 (10.6-27.0)	3.73 (3.22-4.18)	1.98 (1.71-2.22)	536 (248-963)	138.4 (64.0-248.7)	0.62 (0.55-0.70)	1.60 (1.42-1.80)

Location	All ages				Children under 5				Adults over 70			
	Deaths (95% UI)	Deaths per 100,000 (95% UI)	Millions of episodes (95% UI)	Incidence per person (95% UI)	Deaths (95% UI)	Deaths per 100,000 (95% UI)	Millions of episodes (95% UI)	Incidence per person (95% UI)	Deaths (95% UI)	Deaths per 100,000 (95% UI)	Millions of episodes (95% UI)	Incidence per person (95% UI)
Indonesia	49,676 (28,683-70,669)	19.3 (11.1-27.4)	209.67 (197.00-223.28)	0.81 (0.76-0.87)	7,566 (6,249-9,258)	33.3 (27.5-40.7)	42.51 (35.51-51.56)	1.87 (1.56-2.27)	24,947 (11,304-40,452)	304.3 (137.9-493.4)	13.46 (12.01-15.04)	1.64 (1.46-1.84)
Laos	1,176 (612-2,020)	16.3 (8.5-28.0)	5.59 (5.19-5.98)	0.77 (0.72-0.83)	835 (358-1,578)	73.9 (31.6-139.6)	2.11 (1.79-2.41)	1.87 (1.58-2.14)	163 (66-308)	102.0 (41.3-193.3)	0.19 (0.17-0.21)	1.19 (1.05-1.34)
Malaysia	497 (282-764)	1.6 (0.9-2.5)	22.32 (20.40-24.37)	0.73 (0.66-0.79)	52 (35-78)	2.0 (1.4-3.0)	3.59 (2.81-4.56)	1.40 (1.09-1.78)	249 (133-420)	23.1 (12.4-39.0)	1.08 (0.94-1.23)	1.00 (0.87-1.15)
Maldives	3 (2-5)	0.9 (0.5-1.3)	0.19 (0.18-0.20)	0.52 (0.49-0.56)	1 (0-1)	1.6 (1.0-2.5)	0.03 (0.03-0.04)	1.05 (0.87-1.30)	2 (1-3)	14.2 (6.8-23.2)	0.01 (0.01-0.01)	0.79 (0.70-0.89)
Mauritius	29 (25-33)	2.2 (2.0-2.6)	0.85 (0.78-0.92)	0.67 (0.62-0.72)	4 (3-6)	6.1 (4.3-8.5)	0.08 (0.07-0.10)	1.23 (1.02-1.53)	15 (13-18)	20.0 (16.9-23.6)	0.05 (0.05-0.06)	0.72 (0.65-0.81)
Myanmar	7,235 (4,264-10,953)	13.3 (7.8-20.1)	41.33 (38.77-43.99)	0.76 (0.71-0.81)	1,171 (597-2,096)	25.2 (12.8-45.0)	7.36 (6.06-8.83)	1.58 (1.30-1.90)	3,344 (1,716-5,429)	188.4 (96.7-305.8)	2.63 (2.34-2.93)	1.48 (1.32-1.65)
Philippines	6,848 (5,182-8,885)	6.7 (5.1-8.7)	58.87 (55.08-63.05)	0.57 (0.54-0.62)	2,664 (1,715-4,118)	23.0 (14.8-35.6)	15.49 (13.31-18.21)	1.34 (1.15-1.57)	1,432 (1,069-1,941)	51.8 (38.7-70.3)	2.20 (1.98-2.43)	0.79 (0.72-0.88)
Sri Lanka	541 (267-1,025)	2.6 (1.3-4.9)	12.36 (11.52-13.18)	0.60 (0.56-0.64)	19 (10-32)	1.2 (0.6-2.1)	1.79 (1.48-2.18)	1.18 (0.98-1.44)	360 (167-683)	30.6 (14.2-58.1)	1.18 (1.05-1.32)	1.00 (0.90-1.12)
Seychelles	2 (1-3)	1.9 (1.1-3.1)	0.06 (0.06-0.07)	0.65 (0.60-0.69)	0 (0-0)	1.5 (1.0-2.1)	0.01 (0.01-0.01)	1.32 (1.07-1.64)	1 (1-2)	23.4 (13.3-40.2)	0.00 (0.00-0.01)	0.97 (0.86-1.08)
Thailand	3,899 (2,166-6,842)	5.8 (3.2-10.1)	42.55 (39.95-45.43)	0.63 (0.59-0.67)	83 (52-129)	2.7 (1.7-4.2)	3.69 (3.15-4.43)	1.19 (1.02-1.43)	2,559 (1,407-4,666)	53.9 (29.6-98.3)	5.47 (4.85-6.12)	1.15 (1.02-1.29)
Timor-Leste	271 (141-444)	23.4 (12.2-38.3)	1.31 (1.20-1.42)	1.13 (1.04-1.22)	203 (106-352)	122.8 (63.8-212.7)	0.43 (0.36-0.52)	2.60 (2.18-3.15)	36 (3-87)	102.4 (9.5-248.4)	0.06 (0.05-0.07)	1.79 (1.57-2.01)
Vietnam	1,959 (867-3,711)	2.1 (0.9-3.9)	61.44 (57.20-65.74)	0.65 (0.61-0.70)	107 (63-178)	1.4 (0.8-2.4)	10.09 (8.46-12.07)	1.34 (1.12-1.60)	1,420 (550-3,018)	31.9 (12.4-67.8)	4.87 (4.36-5.40)	1.09 (0.98-1.21)

Location	All ages				Children under 5				Adults over 70			
	Deaths (95% UI)	Deaths per 100,000 (95% UI)	Millions of episodes (95% UI)	Incidence per person (95% UI)	Deaths (95% UI)	Deaths per 100,000 (95% UI)	Millions of episodes (95% UI)	Incidence per person (95% UI)	Deaths (95% UI)	Deaths per 100,000 (95% UI)	Millions of episodes (95% UI)	Incidence per person (95% UI)
**Oceania**	**2,312 (1,378-3,591)**	**20.6 (12.3-32.0)**	**13.24 (12.41-14.16)**	**1.18 (1.11-1.26)**	**414 (224-725)**	**29.3 (15.9-51.3)**	**2.59 (2.17-3.11)**	**1.83 (1.53-2.20)**	**914 (492-1,605)**	**366.8 (197.5-643.7)**	**0.60 (0.54-0.67)**	**2.41 (2.15-2.71)**
American Samoa	1 (1-2)	1.6 (0.9-2.5)	0.08 (0.07-0.09)	0.98 (0.91-1.06)	0 (0-0)	2.1 (1.3-3.3)	0.01 (0.01-0.01)	1.39 (1.15-1.69)	1 (0-1)	40.4 (20.0-68.4)	0.00 (0.00-0.00)	1.65 (1.45-1.86)
Federated States of Micronesia	4 (2-7)	3.8 (2.3-7.2)	0.09 (0.09-0.10)	0.92 (0.85-1.00)	0 (0-1)	3.5 (1.7-6.5)	0.01 (0.01-0.01)	1.23 (1.02-1.49)	2 (1-4)	84.1 (45.3-166.8)	0.00 (0.00-0.01)	1.77 (1.57-1.98)
Fiji	73 (43-107)	8.5 (5.0-12.4)	0.75 (0.70-0.80)	0.87 (0.81-0.93)	14 (7-26)	27.7 (13.6-51.7)	0.08 (0.07-0.09)	1.54 (1.31-1.86)	32 (18-55)	105.0 (56.7-179.0)	0.05 (0.05-0.06)	1.66 (1.48-1.88)
Guam	2 (1-3)	1.0 (0.6-1.6)	0.19 (0.17-0.20)	1.07 (1.00-1.15)	0 (0-0)	0.9 (0.5-1.4)	0.03 (0.02-0.03)	1.50 (1.23-1.84)	1 (1-2)	12.6 (6.5-20.4)	0.01 (0.01-0.01)	1.33 (1.18-1.48)
Kiribati	32 (17-51)	27.9 (15.1-44.6)	0.12 (0.12-0.13)	1.10 (1.03-1.18)	7 (3-13)	51.5 (23.0-99.7)	0.03 (0.02-0.03)	1.85 (1.54-2.27)	15 (6-27)	568.7 (243.1-1,061.3)	0.01 (0.01-0.01)	2.48 (2.20-2.79)
Marshall Islands	2 (1-4)	2.8 (1.8-4.9)	0.07 (0.06-0.07)	0.93 (0.86-1.00)	0 (0-1)	4.9 (2.1-9.8)	0.01 (0.01-0.02)	1.37 (1.14-1.64)	1 (0-2)	54.9 (29.3-109.7)	0.00 (0.00-0.00)	1.71 (1.51-1.92)
Northern Mariana Islands	1 (0-1)	0.4 (0.2-0.6)	0.14 (0.13-0.15)	1.11 (1.02-1.20)	0 (0-0)	0.3 (0.1-0.6)	0.03 (0.02-0.04)	2.00 (1.60-2.55)	0 (0-0)	24.0 (11.5-40.4)	0.00 (0.00-0.00)	1.49 (1.34-1.66)
Papua New Guinea	2,075 (1,214-3,262)	26.4 (15.5-41.5)	9.62 (8.99-10.34)	1.23 (1.15-1.32)	362 (172-656)	33.6 (15.9-60.9)	2.01 (1.68-2.43)	1.87 (1.56-2.25)	810 (427-1,434)	616.1 (325.1-1,091.4)	0.36 (0.32-0.41)	2.73 (2.42-3.11)
Samoa	6 (3-10)	2.8 (1.7-5.2)	0.21 (0.19-0.23)	1.06 (0.98-1.14)	0 (0-1)	1.5 (0.6-3.3)	0.04 (0.03-0.05)	1.51 (1.24-1.86)	4 (2-8)	55.9 (28.9-111.0)	0.01 (0.01-0.01)	1.84 (1.64-2.06)
Solomon Islands	76 (45-129)	12.8 (7.5-21.6)	0.71 (0.66-0.76)	1.18 (1.10-1.27)	21 (11-35)	24.8 (13.4-42.3)	0.16 (0.13-0.19)	1.87 (1.56-2.24)	30 (14-58)	248.7 (114.0-480.1)	0.03 (0.03-0.03)	2.48 (2.19-2.79)
Tonga	4 (2-7)	3.6 (2.1-6.1)	0.11 (0.11-0.12)	1.05 (0.97-1.13)	1 (0-1)	4.4 (2.0-8.2)	0.02 (0.02-0.02)	1.43 (1.21-1.71)	3 (1-5)	59.4 (31.2-110.3)	0.01 (0.01-0.01)	1.79 (1.59-2.00)

Location	All ages				Children under 5				Adults over 70			
	Deaths (95% UI)	Deaths per 100,000 (95% UI)	Millions of episodes (95% UI)	Incidence per person (95% UI)	Deaths (95% UI)	Deaths per 100,000 (95% UI)	Millions of episodes (95% UI)	Incidence per person (95% UI)	Deaths (95% UI)	Deaths per 100,000 (95% UI)	Millions of episodes (95% UI)	Incidence per person (95% UI)
Vanuatu	36 (22-63)	13.2 (8.1-22.7)	0.36 (0.33-0.38)	1.29 (1.20-1.39)	8 (4-16)	20.6 (9.8-38.3)	0.08 (0.07-0.10)	1.98 (1.65-2.35)	16 (8-31)	220.2 (111.2-425.9)	0.02 (0.02-0.02)	2.62 (2.31-2.95)
**North Africa and Middle East**	**34,998 (26,768-44,682)**	**6.1 (4.7-7.8)**	**426.33 (396.21-457.69)**	**0.74 (0.69-0.80)**	**26,373 (19,539-34,818)**	**41.7 (30.9-55.1)**	**152.83 (131.12-176.50)**	**2.42 (2.08-2.79)**	**4,751 (2,708-9,256)**	**26.9 (15.4-52.5)**	**15.79 (14.30-17.52)**	**0.90 (0.81-0.99)**
Afghanistan	4,910 (2,960-7,423)	14.7 (8.9-22.2)	38.01 (34.57-41.72)	1.14 (1.03-1.25)	4,285 (2,366-6,848)	86.4 (47.7-138.1)	16.61 (14.00-19.81)	3.35 (2.83-4.00)	207 (75-474)	45.8 (16.7-104.8)	0.56 (0.49-0.63)	1.23 (1.09-1.39)
Algeria	684 (438-1,081)	1.7 (1.1-2.7)	23.68 (22.17-25.43)	0.59 (0.55-0.63)	218 (115-378)	4.8 (2.5-8.3)	7.55 (6.55-8.53)	1.66 (1.44-1.88)	333 (166-594)	20.8 (10.4-37.1)	1.27 (1.14-1.43)	0.79 (0.71-0.89)
Bahrain	9 (5-15)	0.6 (0.4-1.0)	0.71 (0.65-0.77)	0.51 (0.47-0.55)	1 (1-1)	0.8 (0.5-1.3)	0.16 (0.13-0.20)	1.64 (1.35-2.05)	4 (2-7)	21.6 (10.3-35.2)	0.02 (0.02-0.02)	0.84 (0.75-0.94)
Egypt	10,542 (6,592-16,571)	11.5 (7.2-18.1)	67.92 (63.56-72.66)	0.74 (0.69-0.79)	9,044 (5,240-14,647)	82.9 (48.0-134.3)	28.07 (24.76-31.37)	2.57 (2.27-2.88)	811 (338-1,871)	28.6 (11.9-65.9)	2.55 (2.27-2.87)	0.90 (0.80-1.01)
Iran	1,202 (781-2,030)	1.5 (1.0-2.5)	62.47 (57.55-67.96)	0.77 (0.71-0.84)	352 (132-797)	4.4 (1.6-9.9)	18.19 (15.01-22.21)	2.26 (1.86-2.75)	530 (308-1,002)	20.6 (12.0-38.9)	2.65 (2.34-3.00)	1.03 (0.91-1.17)
Iraq	2,467 (1,329-3,661)	6.3 (3.4-9.3)	33.24 (29.81-36.40)	0.84 (0.76-0.92)	1,675 (719-2,839)	21.7 (9.3-36.8)	17.29 (14.16-20.12)	2.24 (1.84-2.61)	340 (169-683)	47.1 (23.4-94.6)	0.66 (0.59-0.74)	0.91 (0.81-1.02)
Jordan	50 (35-72)	0.6 (0.4-0.9)	4.31 (3.96-4.71)	0.56 (0.51-0.61)	24 (15-35)	2.5 (1.6-3.7)	1.54 (1.29-1.83)	1.61 (1.34-1.91)	16 (8-28)	8.5 (4.1-15.0)	0.12 (0.11-0.14)	0.64 (0.57-0.72)
Kuwait	7 (5-9)	0.2 (0.1-0.2)	2.32 (2.15-2.52)	0.59 (0.55-0.64)	4 (3-6)	1.4 (0.9-2.2)	0.53 (0.43-0.64)	1.86 (1.52-2.28)	1 (1-1)	2.5 (1.9-3.2)	0.03 (0.02-0.03)	0.66 (0.59-0.74)
Lebanon	68 (35-126)	1.2 (0.6-2.2)	3.61 (3.37-3.87)	0.62 (0.58-0.66)	10 (5-18)	3.2 (1.7-5.7)	0.68 (0.58-0.80)	2.13 (1.82-2.50)	46 (19-92)	13.7 (5.8-27.4)	0.31 (0.27-0.34)	0.92 (0.81-1.03)
Libya	62 (40-97)	1.0 (0.7-1.6)	5.07 (4.65-5.53)	0.82 (0.76-0.90)	20 (11-36)	4.4 (2.3-7.7)	1.04 (0.87-1.27)	2.25 (1.87-2.74)	27 (14-46)	14.7 (7.8-25.1)	0.19 (0.17-0.21)	1.04 (0.92-1.16)
Morocco	1,417 (938-2,083)	4.2 (2.8-6.2)	19.02 (17.68-20.52)	0.56 (0.53-0.61)	911 (547-1,424)	39.7 (23.9-62.1)	4.81 (4.03-5.80)	2.10 (1.76-2.53)	356 (179-667)	25.0 (12.6-46.8)	1.16 (1.03-1.28)	0.81 (0.72-0.90)
Palestine	57 (35-86)	1.1 (0.7-1.7)	4.30 (3.85-4.74)	0.83 (0.74-0.92)	34 (16-58)	3.1 (1.5-5.3)	2.24 (1.85-2.66)	2.07 (1.70-2.45)	12 (7-22)	14.8 (8.7-26.4)	0.07 (0.06-0.07)	0.78 (0.69-0.88)

Location	All ages Deaths (95% UI)	All ages Deaths per 100,000 (95% UI)	All ages Millions of episodes (95% UI)	All ages Incidence per person (95% UI)	Children under 5 Deaths (95% UI)	Children under 5 Deaths per 100,000 (95% UI)	Children under 5 Millions of episodes (95% UI)	Children under 5 Incidence per person (95% UI)	Adults over 70 Deaths (95% UI)	Adults over 70 Deaths per 100,000 (95% UI)	Adults over 70 Millions of episodes (95% UI)	Adults over 70 Incidence per person (95% UI)
Oman	20 (11-28)	0.4 (0.2-0.6)	2.98 (2.71-3.25)	0.63 (0.58-0.69)	4 (2-6)	0.9 (0.5-1.4)	0.85 (0.69-1.03)	1.91 (1.56-2.32)	9 (4-14)	11.5 (5.4-19.0)	0.06 (0.05-0.07)	0.83 (0.72-0.94)
Qatar	3 (2-4)	0.1 (0.1-0.2)	0.63 (0.58-0.68)	0.28 (0.25-0.30)	1 (1-2)	1.1 (0.6-1.9)	0.13 (0.11-0.16)	1.06 (0.89-1.31)	0 (0-1)	1.8 (1.0-3.6)	0.01 (0.01-0.01)	0.40 (0.35-0.45)
Saudi Arabia	281 (164-412)	0.9 (0.5-1.3)	16.12 (14.98-17.41)	0.51 (0.48-0.55)	41 (28-59)	1.7 (1.1-2.4)	3.27 (2.69-4.00)	1.31 (1.08-1.60)	153 (67-246)	27.0 (11.9-43.6)	0.49 (0.44-0.54)	0.86 (0.78-0.95)
Sudan	5,471 (2,706-9,551)	13.9 (6.9-24.3)	36.99 (33.88-40.07)	0.94 (0.86-1.02)	3,406 (1,271-7,024)	81.2 (30.3-167.5)	13.42 (11.11-15.85)	3.20 (2.65-3.78)	1,096 (338-2,554)	133.8 (41.3-311.5)	1.04 (0.92-1.18)	1.27 (1.12-1.43)
Syria	68 (45-104)	0.4 (0.2-0.6)	10.36 (9.50-11.35)	0.57 (0.52-0.62)	29 (15-49)	1.6 (0.8-2.8)	2.79 (2.31-3.43)	1.58 (1.31-1.94)	22 (11-44)	4.5 (2.2-9.2)	0.32 (0.28-0.36)	0.67 (0.60-0.75)
Tunisia	187 (98-389)	1.7 (0.9-3.5)	6.21 (5.81-6.63)	0.55 (0.52-0.59)	19 (11-31)	2.3 (1.4-3.7)	1.46 (1.24-1.74)	1.75 (1.49-2.09)	128 (56-280)	22.3 (9.7-48.7)	0.49 (0.44-0.55)	0.86 (0.77-0.96)
Turkey	639 (427-1,032)	0.8 (0.5-1.3)	47.57 (44.26-51.54)	0.60 (0.56-0.65)	215 (123-349)	3.5 (2.0-5.7)	11.50 (9.49-13.99)	1.87 (1.54-2.27)	310 (174-605)	7.4 (4.2-14.5)	3.21 (2.88-3.59)	0.77 (0.69-0.86)
United Arab Emirates	44 (21-72)	0.5 (0.2-0.7)	5.04 (4.63-5.49)	0.53 (0.48-0.57)	6 (3-11)	0.7 (0.3-1.3)	1.59 (1.30-1.96)	1.95 (1.59-2.40)	12 (3-27)	23.0 (6.7-52.9)	0.04 (0.03-0.04)	0.77 (0.69-0.86)
Yemen	6,783 (3,026-11,281)	24.1 (10.8-40.1)	35.40 (32.16-38.73)	1.26 (1.14-1.38)	6,052 (2,279-10,622)	130.8 (49.3-229.6)	18.99 (16.24-21.96)	4.10 (3.51-4.75)	334 (138-689)	79.5 (32.7-163.8)	0.54 (0.47-0.60)	1.27 (1.12-1.43)
**South Asia**	**873,865 (605,184-1,356,359)**	**51.4 (35.6-79.8)**	**1,487.51 (1,408.03-1,568.11)**	**0.88 (0.83-0.92)**	**101,927 (85,817-122,100)**	**66.4 (55.9-79.5)**	**228.16 (198.92-263.84)**	**1.49 (1.29-1.72)**	**508,455 (337,374-798,290)**	**877.4 (582.2-1,377.5)**	**173.70 (156.34-193.58)**	**3.00 (2.70-3.34)**
Bangladesh	29,203 (18,671-53,689)	18.0 (11.5-33.2)	123.47 (115.82-130.98)	0.76 (0.72-0.81)	3,149 (1,966-4,585)	22.0 (13.7-32.0)	20.06 (17.31-23.70)	1.40 (1.21-1.65)	18,541 (10,688-33,394)	347.7 (200.4-626.3)	13.43 (11.95-15.02)	2.52 (2.24-2.82)
Bhutan	78 (17-170)	9.8 (2.1-21.4)	1.94 (1.83-2.06)	2.44 (2.30-2.58)	15 (7-27)	19.8 (9.3-34.3)	0.36 (0.32-0.40)	4.66 (4.16-5.12)	45 (2-110)	172.5 (9.2-424.5)	0.17 (0.16-0.17)	6.42 (6.15-6.61)

Location	All ages				Children under 5				Adults over 70			
	Deaths (95% UI)	Deaths per 100,000 (95% UI)	Millions of episodes (95% UI)	Incidence per person (95% UI)	Deaths (95% UI)	Deaths per 100,000 (95% UI)	Millions of episodes (95% UI)	Incidence per person (95% UI)	Deaths (95% UI)	Deaths per 100,000 (95% UI)	Millions of episodes (95% UI)	Incidence per person (95% UI)
India	777,991 (533,272-1,227,903)	59.1 (40.5-93.3)	1,133.26 (1,071.40-1,198.81)	0.86 (0.81-0.91)	66,157 (56,001-78,160)	59.0 (49.9-69.7)	134.94 (115.53-159.12)	1.20 (1.03-1.42)	467,952 (308,075-729,088)	1,013.0 (666.9-1,578.3)	141.20 (126.84-157.36)	3.06 (2.75-3.41)
Nepal	8,889 (4,926-14,795)	29.6 (16.4-49.2)	32.40 (30.72-34.21)	1.08 (1.02-1.14)	916 (482-1,601)	22.9 (12.1-40.1)	8.93 (8.01-9.96)	2.24 (2.01-2.49)	5,307 (2,644-8,907)	548.2 (273.1-920.0)	3.14 (2.81-3.48)	3.24 (2.90-3.59)
Pakistan	57,704 (39,125-90,218)	30.2 (20.5-47.2)	196.43 (184.38-208.08)	1.03 (0.97-1.09)	31,690 (21,654-43,502)	137.4 (93.9-188.6)	63.86 (55.97-72.52)	2.77 (2.43-3.14)	16,609 (7,550-36,920)	306.0 (139.1-680.1)	15.76 (13.87-17.68)	2.90 (2.55-3.26)
**Sub-Saharan Africa**	**606,024 (469,707-798,314)**	**61.8 (47.9-81.5)**	**1,028.94 (966.50-1,098.09)**	**1.05 (0.99-1.12)**	**290,724 (243,545-342,557)**	**185.7 (155.5-218.8)**	**371.24 (322.16-428.79)**	**2.37 (2.06-2.74)**	**105,863 (63,487-166,068)**	**589.3 (353.4-924.4)**	**35.36 (31.98-39.01)**	**1.97 (1.78-2.17)**
**Southern Sub-Saharan Africa**	**24,952 (18,130-33,765)**	**32.4 (23.6-43.9)**	**80.18 (76.94-83.74)**	**1.04 (1.00-1.09)**	**10,281 (8,270-12,696)**	**119.4 (96.1-147.5)**	**17.54 (15.58-19.96)**	**2.04 (1.81-2.32)**	**5,636 (3,076-9,354)**	**230.3 (125.7-382.2)**	**4.58 (4.29-4.90)**	**1.87 (1.75-2.00)**
Botswana	579 (311-944)	25.2 (13.5-41.1)	1.48 (1.38-1.58)	0.64 (0.60-0.69)	173 (89-293)	65.4 (33.7-110.5)	0.38 (0.32-0.46)	1.43 (1.19-1.75)	123 (46-244)	242.8 (91.4-483.0)	0.05 (0.05-0.06)	1.08 (0.96-1.21)
Lesotho	1,990 (1,247-3,013)	93.2 (58.4-141.1)	2.04 (1.90-2.18)	0.96 (0.89-1.02)	905 (610-1,304)	350.5 (236.1-505.2)	0.57 (0.49-0.66)	2.19 (1.89-2.54)	413 (135-801)	755.5 (247.1-1,466.1)	0.09 (0.08-0.10)	1.64 (1.45-1.85)
Namibia	979 (631-1,391)	39.2 (25.3-55.7)	2.14 (1.98-2.31)	0.86 (0.79-0.92)	579 (334-886)	173.8 (100.4-266.3)	0.73 (0.60-0.86)	2.19 (1.80-2.60)	132 (57-236)	246.8 (106.5-440.4)	0.07 (0.06-0.08)	1.35 (1.19-1.50)
South Africa	12,344 (8,003-18,846)	23.4 (15.2-35.7)	59.92 (57.82-62.22)	1.14 (1.10-1.18)	2,658 (1,883-3,651)	53.0 (37.6-72.9)	10.31 (9.17-11.69)	2.06 (1.83-2.33)	3,999 (2,202-6,905)	205.2 (113.0-354.3)	3.91 (3.68-4.16)	2.01 (1.89-2.14)

Location	All ages				Children under 5				Adults over 70			
	Deaths (95% UI)	Deaths per 100,000 (95% UI)	Millions of episodes (95% UI)	Incidence per person (95% UI)	Deaths (95% UI)	Deaths per 100,000 (95% UI)	Millions of episodes (95% UI)	Incidence per person (95% UI)	Deaths (95% UI)	Deaths per 100,000 (95% UI)	Millions of episodes (95% UI)	Incidence per person (95% UI)
Swaziland	838 (591-1,137)	62.6 (44.2-85.0)	1.22 (1.13-1.33)	0.91 (0.85-0.99)	534 (339-764)	256.0 (162.5-366.5)	0.42 (0.36-0.51)	2.01 (1.70-2.45)	92 (44-168)	329.5 (156.5-602.9)	0.04 (0.04-0.04)	1.43 (1.27-1.61)
Zimbabwe	8,222 (5,752-11,184)	51.5 (36.1-70.1)	13.38 (12.52-14.31)	0.84 (0.78-0.90)	5,432 (3,747-7,487)	214.5 (147.9-295.6)	5.14 (4.50-5.86)	2.03 (1.78-2.31)	877 (412-1,609)	281.2 (132.1-515.5)	0.41 (0.37-0.46)	1.32 (1.17-1.48)
**Western Sub-Saharan Africa**	**270,082 (213,648-339,535)**	**67.8 (53.6-85.3)**	**380.57 (356.06-407.71)**	**0.96 (0.89-1.02)**	**177,262 (139,712-220,546)**	**274.3 (216.2-341.3)**	**157.13 (138.03-180.72)**	**2.43 (2.14-2.80)**	**28,641 (16,799-48,488)**	**454.0 (266.3-768.6)**	**11.29 (10.06-12.57)**	**1.79 (1.60-1.99)**
Benin	8,679 (6,301-11,864)	76.5 (55.6-104.6)	9.81 (9.08-10.67)	0.87 (0.80-0.94)	5,548 (3,710-7,669)	290.4 (194.2-401.4)	3.83 (3.24-4.55)	2.01 (1.70-2.38)	962 (524-1,835)	516.9 (281.5-986.3)	0.31 (0.27-0.35)	1.66 (1.47-1.86)
Burkina Faso	10,544 (7,360-14,483)	56.6 (39.5-77.7)	19.63 (18.30-21.28)	1.05 (0.98-1.14)	4,631 (2,837-7,223)	144.9 (88.8-226.1)	7.35 (6.40-8.65)	2.30 (2.00-2.71)	1,745 (978-2,892)	691.4 (387.4-1,145.9)	0.55 (0.49-0.62)	2.20 (1.95-2.45)
Cameroon	10,460 (7,374-15,016)	43.6 (30.7-62.6)	23.98 (21.89-26.02)	1.00 (0.91-1.08)	4,893 (2,915-7,680)	126.2 (75.2-198.1)	10.19 (8.50-12.05)	2.63 (2.19-3.11)	2,082 (1,161-3,779)	448.5 (250.1-814.2)	0.86 (0.76-0.97)	1.85 (1.64-2.09)
Cape Verde	34 (23-56)	6.3 (4.1-10.3)	0.40 (0.37-0.43)	0.73 (0.67-0.79)	9 (6-13)	11.8 (7.3-17.6)	0.11 (0.09-0.13)	1.41 (1.15-1.72)	13 (7-24)	72.6 (39.0-130.3)	0.03 (0.02-0.03)	1.40 (1.25-1.57)
Chad	18,036 (13,122-23,618)	125.0 (90.9-163.6)	20.13 (18.45-21.72)	1.39 (1.28-1.50)	13,186 (9,108-18,132)	499.2 (344.8-686.4)	9.90 (8.30-11.29)	3.75 (3.14-4.27)	1,498 (834-2,668)	718.2 (399.9-1,278.8)	0.50 (0.44-0.57)	2.41 (2.12-2.72)
Cote d'Ivoire	14,380 (9,830-20,385)	62.4 (42.6-88.4)	22.79 (20.93-24.75)	0.99 (0.91-1.07)	9,916 (6,093-14,930)	278.6 (171.2-419.5)	9.62 (8.08-11.32)	2.70 (2.27-3.18)	1,275 (656-2,566)	325.4 (167.4-654.9)	0.68 (0.60-0.77)	1.74 (1.53-1.96)

Location	All ages: Deaths (95% UI)	All ages: Deaths per 100,000 (95% UI)	All ages: Millions of episodes (95% UI)	All ages: Incidence per person (95% UI)	Children under 5: Deaths (95% UI)	Children under 5: Deaths per 100,000 (95% UI)	Children under 5: Millions of episodes (95% UI)	Children under 5: Incidence per person (95% UI)	Adults over 70: Deaths (95% UI)	Adults over 70: Deaths per 100,000 (95% UI)	Adults over 70: Millions of episodes (95% UI)	Adults over 70: Incidence per person (95% UI)
The Gambia	694 (470-1,016)	34.0 (23.0-49.7)	2.00 (1.84-2.17)	0.98 (0.90-1.06)	416 (281-594)	113.5 (76.8-162.1)	0.89 (0.76-1.05)	2.44 (2.07-2.87)	88 (42-181)	306.3 (145.7-634.0)	0.05 (0.05-0.06)	1.78 (1.58-2.00)
Ghana	4,289 (2,978-6,878)	15.2 (10.5-24.4)	21.74 (20.23-23.32)	0.77 (0.72-0.83)	1,455 (898-2,238)	33.5 (20.7-51.5)	7.93 (6.76-9.32)	1.82 (1.56-2.14)	974 (529-1,849)	178.1 (96.8-338.1)	0.81 (0.72-0.91)	1.48 (1.31-1.67)
Guinea	5,840 (4,032-8,761)	45.3 (31.3-68.0)	13.39 (12.36-14.41)	1.04 (0.96-1.12)	2,608 (1,550-4,199)	128.1 (76.2-206.4)	5.26 (4.42-6.10)	2.59 (2.17-3.00)	1,040 (585-1,883)	461.2 (259.5-835.0)	0.44 (0.39-0.50)	1.96 (1.73-2.21)
Guinea-Bissau	1,040 (724-1,565)	54.7 (38.0-82.2)	1.76 (1.61-1.92)	0.92 (0.85-1.01)	503 (313-727)	162.4 (100.9-234.4)	0.68 (0.56-0.83)	2.20 (1.82-2.67)	155 (81-288)	463.4 (241.7-862.0)	0.06 (0.05-0.06)	1.69 (1.49-1.91)
Liberia	3,842 (2,671-5,631)	83.2 (57.8-121.9)	5.78 (5.32-6.22)	1.25 (1.15-1.35)	2,146 (1,415-2,980)	301.0 (198.5-418.1)	2.28 (1.92-2.65)	3.19 (2.70-3.71)	565 (292-1,073)	698.4 (361.0-1,324.9)	0.19 (0.17-0.21)	2.35 (2.08-2.62)
Mali	18,025 (12,834-24,209)	100.4 (71.5-134.8)	18.22 (16.86-19.69)	1.02 (0.94-1.10)	11,386 (7,502-16,610)	361.5 (238.2-527.3)	7.35 (6.36-8.65)	2.33 (2.02-2.75)	2,343 (1,229-4,101)	879.7 (461.5-1,540.1)	0.56 (0.49-0.63)	2.09 (1.84-2.35)
Mauritania	1,522 (1,006-2,471)	37.4 (24.7-60.7)	3.91 (3.65-4.16)	0.96 (0.90-1.02)	583 (401-833)	113.4 (78.0-161.9)	1.24 (1.08-1.42)	2.42 (2.10-2.76)	318 (159-631)	406.7 (202.7-806.0)	0.15 (0.13-0.17)	1.89 (1.68-2.12)
Niger	21,037 (15,015-28,949)	104.8 (74.8-144.2)	23.37 (21.61-25.40)	1.16 (1.08-1.26)	13,850 (8,632-20,603)	376.0 (234.3-559.3)	9.86 (8.44-11.73)	2.68 (2.29-3.18)	2,042 (1,052-3,750)	698.3 (359.7-1,282.5)	0.66 (0.58-0.74)	2.25 (1.99-2.53)
Nigeria	135,255 (97,563-175,438)	73.2 (52.8-95.0)	164.02 (153.73-176.41)	0.89 (0.83-0.95)	97,854 (67,460-134,496)	331.3 (228.4-455.4)	68.84 (61.03-79.42)	2.33 (2.07-2.69)	11,102 (5,769-19,648)	404.2 (210.1-715.4)	4.54 (4.00-5.12)	1.65 (1.46-1.86)

Location	All ages				Children under 5				Adults over 70			
	Deaths (95% UI)	Deaths per 100,000 (95% UI)	Millions of episodes (95% UI)	Incidence per person (95% UI)	Deaths (95% UI)	Deaths per 100,000 (95% UI)	Millions of episodes (95% UI)	Incidence per person (95% UI)	Deaths (95% UI)	Deaths per 100,000 (95% UI)	Millions of episodes (95% UI)	Incidence per person (95% UI)
Sao Tome and Principe	30 (18-48)	15.2 (9.2-24.1)	0.18 (0.17-0.20)	0.91 (0.84-0.99)	18 (9-30)	53.2 (28.0-89.9)	0.08 (0.07-0.09)	2.32 (1.96-2.73)	5 (2-11)	126.9 (50.0-275.5)	0.01 (0.01-0.01)	1.66 (1.47-1.86)
Senegal	7,082 (5,241-9,853)	45.8 (33.9-63.8)	15.79 (14.86-16.82)	1.02 (0.96-1.09)	3,494 (2,406-4,864)	137.5 (94.7-191.4)	6.76 (6.00-7.55)	2.66 (2.36-2.97)	1,241 (699-2,128)	449.9 (253.5-771.4)	0.51 (0.45-0.57)	1.84 (1.64-2.07)
Sierra Leone	6,310 (4,467-8,445)	95.3 (67.4-127.5)	6.48 (5.99-7.04)	0.98 (0.90-1.06)	3,761 (2,434-5,476)	361.6 (234.1-526.6)	2.42 (2.05-2.91)	2.33 (1.97-2.80)	640 (337-1,074)	690.2 (363.4-1,158.2)	0.17 (0.15-0.19)	1.88 (1.65-2.10)
Togo	2,981 (2,019-4,818)	40.2 (27.2-65.0)	7.19 (6.70-7.67)	0.97 (0.90-1.03)	1,006 (609-1,554)	92.0 (55.7-142.1)	2.52 (2.16-2.93)	2.31 (1.97-2.68)	554 (294-1,031)	479.3 (254.7-891.6)	0.22 (0.19-0.25)	1.92 (1.68-2.15)
**Eastern Sub-Saharan Africa**	**242,217 (174,668-330,016)**	**62.6 (45.1-85.3)**	**425.97 (399.18-454.18)**	**1.10 (1.03-1.17)**	**72,836 (60,186-85,260)**	**116.4 (96.2-136.3)**	**137.27 (118.57-159.02)**	**2.19 (1.90-2.54)**	**58,013 (35,154-91,283)**	**807.3 (489.2-1,270.3)**	**15.22 (13.69-16.83)**	**2.12 (1.90-2.34)**
Burundi	13,375 (9,504-19,359)	115.6 (82.1-167.3)	14.86 (13.85-15.89)	1.28 (1.20-1.37)	7,633 (4,992-11,309)	356.3 (233.0-527.9)	6.18 (5.39-7.05)	2.88 (2.51-3.29)	1,642 (843-2,784)	978.4 (502.1-1,658.9)	0.36 (0.32-0.41)	2.16 (1.90-2.43)
Comoros	412 (239-681)	52.9 (30.7-87.4)	0.94 (0.87-1.00)	1.20 (1.12-1.29)	68 (37-122)	73.6 (39.5-131.8)	0.22 (0.19-0.26)	2.39 (2.02-2.79)	101 (52-175)	752.6 (389.4-1,301.0)	0.03 (0.03-0.03)	2.27 (2.00-2.55)
Djibouti	319 (181-569)	33.0 (18.7-58.7)	0.81 (0.75-0.88)	0.84 (0.77-0.91)	78 (38-143)	45.5 (22.0-83.1)	0.27 (0.23-0.34)	1.59 (1.31-1.96)	88 (41-181)	388.0 (180.1-798.8)	0.03 (0.03-0.04)	1.44 (1.28-1.62)
Eritrea	3,315 (2,314-4,843)	62.2 (43.4-90.8)	5.93 (5.48-6.41)	1.11 (1.03-1.20)	1,233 (750-1,895)	155.0 (94.3-238.2)	1.87 (1.55-2.28)	2.35 (1.94-2.87)	564 (302-978)	731.0 (391.7-1,268.3)	0.16 (0.14-0.18)	2.05 (1.81-2.30)

Location	All ages				Children under 5				Adults over 70			
	Deaths (95% UI)	Deaths per 100,000 (95% UI)	Millions of episodes (95% UI)	Incidence per person (95% UI)	Deaths (95% UI)	Deaths per 100,000 (95% UI)	Millions of episodes (95% UI)	Incidence per person (95% UI)	Deaths (95% UI)	Deaths per 100,000 (95% UI)	Millions of episodes (95% UI)	Incidence per person (95% UI)
Ethiopia	57,101 (38,903-83,133)	55.8 (38.0-81.2)	111.98 (105.26-119.06)	1.09 (1.03-1.16)	14,997 (10,176-20,493)	98.1 (66.6-134.1)	34.30 (29.95-38.18)	2.24 (1.96-2.50)	15,318 (8,300-24,047)	716.1 (388.0-1,124.2)	4.40 (3.92-4.88)	2.06 (1.83-2.28)
Kenya	49,889 (32,343-73,459)	107.1 (69.4-157.7)	58.25 (54.77-62.09)	1.25 (1.18-1.33)	6,367 (5,002-8,082)	96.9 (76.1-122.9)	11.77 (9.97-14.07)	1.79 (1.52-2.14)	14,544 (9,171-23,468)	1,877.3 (1,183.8-3,029.0)	2.34 (2.11-2.59)	3.01 (2.72-3.34)
Madagascar	21,561 (14,900-29,563)	86.5 (59.7-118.5)	27.95 (25.74-30.20)	1.12 (1.03-1.21)	11,647 (6,969-17,980)	299.5 (179.2-462.4)	10.65 (8.84-12.58)	2.74 (2.27-3.24)	2,620 (1,424-5,013)	622.0 (338.1-1,190.1)	0.76 (0.67-0.85)	1.80 (1.59-2.01)
Malawi	11,233 (7,722-16,383)	62.6 (43.0-91.3)	20.51 (18.85-22.27)	1.14 (1.05-1.24)	3,779 (2,443-5,581)	118.0 (76.3-174.3)	8.01 (6.69-9.57)	2.50 (2.09-2.99)	2,949 (1,595-4,874)	803.2 (434.4-1,327.4)	0.78 (0.69-0.88)	2.12 (1.89-2.38)
Mozambique	10,115 (6,167-14,706)	35.1 (21.4-51.1)	23.76 (21.99-25.72)	0.83 (0.76-0.89)	4,092 (2,606-6,037)	82.4 (52.5-121.6)	7.97 (6.82-9.45)	1.60 (1.37-1.90)	2,168 (937-3,969)	375.5 (162.3-687.4)	0.92 (0.82-1.03)	1.60 (1.42-1.79)
Rwanda	4,475 (2,736-7,169)	37.1 (22.7-59.4)	11.58 (10.90-12.40)	0.96 (0.90-1.03)	1,562 (1,006-2,340)	83.4 (53.7-125.0)	3.60 (3.16-4.16)	1.93 (1.69-2.22)	968 (410-1,766)	488.7 (207.0-891.5)	0.37 (0.33-0.41)	1.87 (1.67-2.09)
Somalia	6,975 (4,239-12,336)	67.1 (40.8-118.8)	11.95 (11.21-12.71)	1.15 (1.08-1.22)	1,974 (1,058-3,479)	149.7 (80.2-263.8)	3.21 (2.92-3.55)	2.43 (2.21-2.69)	1,507 (718-3,026)	835.1 (397.5-1,676.5)	0.40 (0.35-0.45)	2.22 (1.96-2.51)
South Sudan	10,666 (6,904-16,706)	78.5 (50.8-123.0)	23.40 (21.51-25.51)	1.72 (1.58-1.88)	3,436 (1,687-6,144)	120.1 (59.0-214.8)	9.13 (7.47-11.09)	3.19 (2.61-3.88)	2,552 (1,486-4,320)	968.1 (563.5-1,638.6)	0.80 (0.71-0.89)	3.03 (2.71-3.38)
Tanzania	26,794 (16,945-40,274)	49.2 (31.1-73.9)	45.58 (42.32-49.25)	0.84 (0.78-0.90)	5,857 (3,763-8,602)	65.1 (41.8-95.6)	13.78 (11.42-16.75)	1.53 (1.27-1.86)	7,667 (4,065-12,675)	712.0 (377.5-1,177.0)	1.82 (1.62-2.03)	1.69 (1.51-1.88)

	All ages				Children under 5				Adults over 70			
**Location**	**Deaths (95% UI)**	**Deaths per 100,000 (95% UI)**	**Millions of episodes (95% UI)**	**Incidence per person (95% UI)**	**Deaths (95% UI)**	**Deaths per 100,000 (95% UI)**	**Millions of episodes (95% UI)**	**Incidence per person (95% UI)**	**Deaths (95% UI)**	**Deaths per 100,000 (95% UI)**	**Millions of episodes (95% UI)**	**Incidence per person (95% UI)**
Uganda	16,406 (11,369-23,242)	40.6 (28.1-57.5)	49.36 (45.18-53.91)	1.22 (1.12-1.33)	7,098 (4,859-10,051)	94.4 (64.6-133.7)	19.57 (16.07-23.70)	2.60 (2.14-3.15)	3,091 (1,474-5,447)	513.2 (244.7-904.3)	1.39 (1.24-1.55)	2.31 (2.05-2.57)
Zambia	9,581 (6,310-13,782)	57.6 (37.9-82.8)	18.83 (17.56-20.19)	1.13 (1.05-1.21)	3,014 (1,954-4,428)	106.0 (68.7-155.8)	6.64 (5.69-7.54)	2.34 (2.00-2.65)	2,232 (1,163-3,685)	749.1 (390.4-1,236.7)	0.65 (0.57-0.72)	2.17 (1.92-2.42)
**Central Sub-Saharan Africa**	**68,599 (50,148-93,556)**	**58.2 (42.6-79.4)**	**142.23 (131.31-154.05)**	**1.21 (1.11-1.31)**	**30,306 (20,536-43,767)**	**145.7 (98.8-210.5)**	**59.30 (50.05-69.41)**	**2.85 (2.41-3.34)**	**13,513 (8,388-22,326)**	**667.7 (414.5-1,103.2)**	**4.27 (3.81-4.76)**	**2.11 (1.88-2.35)**
Angola	15,211 (10,348-22,393)	58.7 (40.0-86.5)	33.46 (30.60-36.91)	1.29 (1.18-1.43)	7,874 (4,493-12,581)	160.5 (91.6-256.4)	14.49 (12.11-17.89)	2.95 (2.47-3.65)	2,322 (1,343-3,995)	673.6 (389.4-1,158.7)	0.78 (0.69-0.87)	2.26 (2.01-2.53)
Central African Republic	6,618 (4,310-9,659)	131.9 (85.9-192.5)	7.23 (6.65-7.77)	1.44 (1.33-1.55)	2,851 (1,755-4,419)	384.2 (236.5-595.5)	2.75 (2.26-3.18)	3.70 (3.05-4.29)	1,502 (797-2,475)	1,281.6 (680.3-2,111.8)	0.29 (0.26-0.33)	2.52 (2.21-2.81)
Congo	2,757 (1,612-4,127)	58.5 (34.2-87.6)	5.33 (4.93-5.76)	1.13 (1.05-1.22)	667 (378-1,109)	89.5 (50.7-148.8)	1.99 (1.66-2.35)	2.67 (2.23-3.15)	871 (431-1,456)	818.5 (404.9-1,368.0)	0.22 (0.20-0.25)	2.07 (1.84-2.33)
Democratic Republic of the Congo	43,372 (29,557-62,223)	54.5 (37.2-78.2)	93.48 (86.15-101.08)	1.18 (1.08-1.27)	18,788 (10,262-31,096)	133.7 (73.0-221.2)	39.22 (32.97-45.89)	2.79 (2.35-3.26)	8,555 (4,984-15,049)	619.2 (360.8-1,089.3)	2.83 (2.51-3.17)	2.05 (1.82-2.29)
Equatorial Guinea	87 (29-233)	10.4 (3.5-27.8)	0.96 (0.89-1.03)	1.15 (1.06-1.24)	12 (6-23)	12.3 (6.0-23.0)	0.26 (0.21-0.31)	2.65 (2.11-3.16)	29 (8-86)	192.9 (51.0-570.8)	0.04 (0.03-0.04)	2.32 (2.07-2.63)
Gabon	552 (290-963)	31.3 (16.4-54.5)	1.77 (1.63-1.89)	1.00 (0.93-1.07)	114 (62-186)	46.6 (25.4-76.0)	0.58 (0.48-0.68)	2.37 (1.94-2.76)	234 (106-435)	398.4 (179.7-740.2)	0.11 (0.10-0.12)	1.87 (1.67-2.10)
